# Regulation of hepatic lipid metabolism by intestine epithelium-derived exosomes

**DOI:** 10.1093/lifemeta/load044

**Published:** 2023-11-21

**Authors:** Tiange Feng, Yuan Liang, Lijun Sun, Lu Feng, Jiajie Min, Michael W Mulholland, Yue Yin, Weizhen Zhang

**Affiliations:** Department of Physiology and Pathophysiology, School of Basic Medical Sciences, and Key Laboratory of Molecular Cardiovascular Science, Ministry of Education, Peking University, Beijing 100191, China; Department of Physiology and Pathophysiology, School of Basic Medical Sciences, and Key Laboratory of Molecular Cardiovascular Science, Ministry of Education, Peking University, Beijing 100191, China; Department of Physiology and Pathophysiology, School of Basic Medical Sciences, and Key Laboratory of Molecular Cardiovascular Science, Ministry of Education, Peking University, Beijing 100191, China; Department of Physiology and Pathophysiology, School of Basic Medical Sciences, and Key Laboratory of Molecular Cardiovascular Science, Ministry of Education, Peking University, Beijing 100191, China; Department of Physiology and Pathophysiology, School of Basic Medical Sciences, and Key Laboratory of Molecular Cardiovascular Science, Ministry of Education, Peking University, Beijing 100191, China; Department of Surgery, University of Michigan Medical Center, Ann Arbor, MI 48109, United States; Department of Pharmacology, School of Basic Medical Sciences, and Key Laboratory of Molecular Cardiovascular Science, Ministry of Education, Peking University, Beijing 100191, China; Department of Physiology and Pathophysiology, School of Basic Medical Sciences, and Key Laboratory of Molecular Cardiovascular Science, Ministry of Education, Peking University, Beijing 100191, China; Department of Surgery, University of Michigan Medical Center, Ann Arbor, MI 48109, United States

**Keywords:** exosome, non-alcoholic fatty liver disease, miR-21a-5p, miR-145a-5p, lipid metabolism

## Abstract

The “gut-liver axis” is critical for the control of hepatic lipid homeostasis, where the intestine affects the liver through multiple pathways, such as nutrient uptake, gastrointestinal hormone release, and gut microbiota homeostasis. Whether intestine-originated exosomes mediate the gut’s influence on liver steatosis remains unknown. Here, we aimed to determine whether intestinal epithelium-derived exosomes (intExos) contribute to the regulation of hepatic lipid metabolism. We found that mouse intExos could be taken up by hepatic cells. Mice fed high-fat diet (HFD) received intExos showed strong resistance to liver steatosis. MicroRNA sequencing of intExos indicated the correlation between miR-21a-5p/miR-145a-5p and hepatic lipid metabolism. Both liver overexpression of miR-21a-5p and intExos containing miR-21a-5p alleviated hepatic steatosis in mice fed with HFD. Mechanistically, miR-21a-5p suppressed the expression of *Ccl1* (C-C motif chemokine ligand 1) in macrophages, as well as lipid transport genes *Cd36* (cluster of differentiation 36) and *Fabp7* (fatty acid binding protein 7) in hepatocytes. Liver-specific inhibition of miR-145a-5p significantly reduced hepatic lipid accumulation in mice fed with HFD through negatively regulating the expression of *Btg1* (BTG anti-proliferation factor 1), leading to an increase of stearoyl-CoA desaturase-1 and lipogenesis. Our study demonstrates that intExos regulate hepatic lipid metabolism and non-alcoholic fatty liver disease (NAFLD) progression via miR-21a-5p and miR-145a-5p pathways, providing novel mediators for the gut-liver crosstalk and potential targets for regulating hepatic lipid metabolism.

## Graphical Abstract

**Graphical Abstract:**
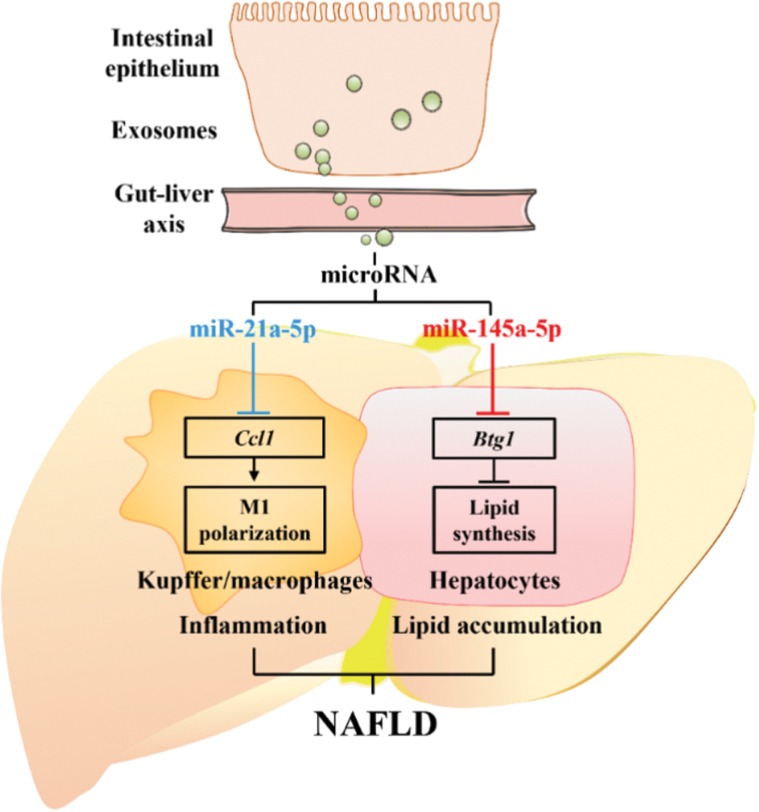

## Introduction

Non-alcoholic fatty liver disease (NAFLD) is one of the most common chronic liver diseases [1], characterized by excessive lipid accumulation in hepatocytes. Its pathogenesis has not yet been fully elucidated. The intestine directly communicates with the liver via the portal vein system [2], which enables its regulation on liver lipid metabolism, as well as the occurrence and development of NAFLD. Among the pathways by which the gut affects the liver, gastrointestinal hormones and the gut microbiota are the most well-studied. Intestinal endocrine cells synthesize and secrete peptide hormones after sensing the fluctuation of nutritional signals and finely regulate the whole body glucose and lipid metabolism homeostasis through the neuroendocrine network [3]. Dysregulation of gut microbiota increases the permeability of the intestinal mucosa, leading to elevated concentrations of microbial metabolites in portal blood, which induces the inflammatory response of liver cells and aggravates their lipid accumulation [4].

Alternative pathways for intestinal regulation of the liver may also exist. One of the promising mediators is exosomes. Exosomes are bilayer lipid membrane extracellular vesicles (EVs) with a diameter of 30−150 nm [5], released by exocytosis after the fusion of multivesicular bodies with the plasma membrane [6]. Exosomes can be taken up by various tissues [7] and, therefore, mediate transport and signaling between distant organs [8]. Nucleic acids [9], proteins, lipids [10], and other substances carried by exosomes widely participate in various biological processes in recipient cells. Emerging evidence has shown that exosomes participate in the regulation of lipid metabolism in different cell types [10]. For example, circulating exosomes deliver fatty acids to the myocardium [11]. Adipocyte-derived exosomes alter lipogenesis and lipid accumulation in other metabolic tissues including the liver [12–14]. It has been demonstrated that liver lipid homeostasis is modified by exosomes from the circulatory system as well [15], although the tissue source of these exosomes is not clear. No research has yet investigated whether liver lipid homeostasis and NAFLD progression are influenced by intestinal exosomes. Studies have suggested that exosome-like particles produced by mouse intestinal mucosa can be taken up by liver natural killer T cells (NKT cells). Prostaglandin E2 (PGE2) delivered by these exosomes induces NKT cell response, leading to suppression of antitumor activity and autoimmunity [16]. This suggests that exosomes may mediate the transmission of information from the intestine to the liver. Because of the mixed population of cell types in the intestinal mucosa, it is currently unclear whether these exosomes are derived from the epithelia, immune cells, or gut microbiota. Neither known are whether exosomes derived from the intestine can be up-taken by hepatocytes and its subsequent impact on lipid accumulation in the liver. To characterize the specific effect of exosomes derived from intestinal epithelia, a critical precondition is the establishment of transgenic mice in which a gene is specifically manipulated in the intestinal epithelia.

Our team has established a line of transgenic mice with intestinal epithelium-specific knockout of leucine-rich repeat G-protein-coupled receptor *Lgr4* (*Villin-Lgr4*^*-/-*^, VL). Relevant to the wild-type littermate controls (WT), VL mice demonstrated a strong resistance to high-fat diet (HFD)-induced lipid dysfunction, exhibited a substantial reduction of liver steatosis (Supplementary Fig. S1), and improved glucose metabolism (unpublished data). The phenotypic changes of VL mice are induced solely by intestinal epithelial cells, making the model suitable for our investigation of intestinal influence on hepatic energy homeostasis. Therefore, we isolated and purified the exosomes from the intestinal epithelia (intestinal epithelium-derived exosomes, intExos) of VL and WT mice, and compared the differences in exosome content between the two groups. Using small RNA sequencing analysis, we determined that the changes in miR-21a-5p and miR-145a-5p concentrations were the most significant. MiR-21a-5p and miR-145a-5p are enriched in both VL and WT intExos, with miR-21a-5p increased in VL intExos compared with that of WT intExos, while miR-145a-5p decreased.

Based on these findings, we hypothesized that miR-21a-5p and miR-145a-5p in intExos serve as the novel mediators for the gut-liver communication, whose function is critical for the regulation of liver lipid metabolism. Taking the approaches of gain or loss of function, we have provided a series of evidence to support this hypothesis.

## Results

### Intestine communicates with the liver via exosomes

**Figure 1:**
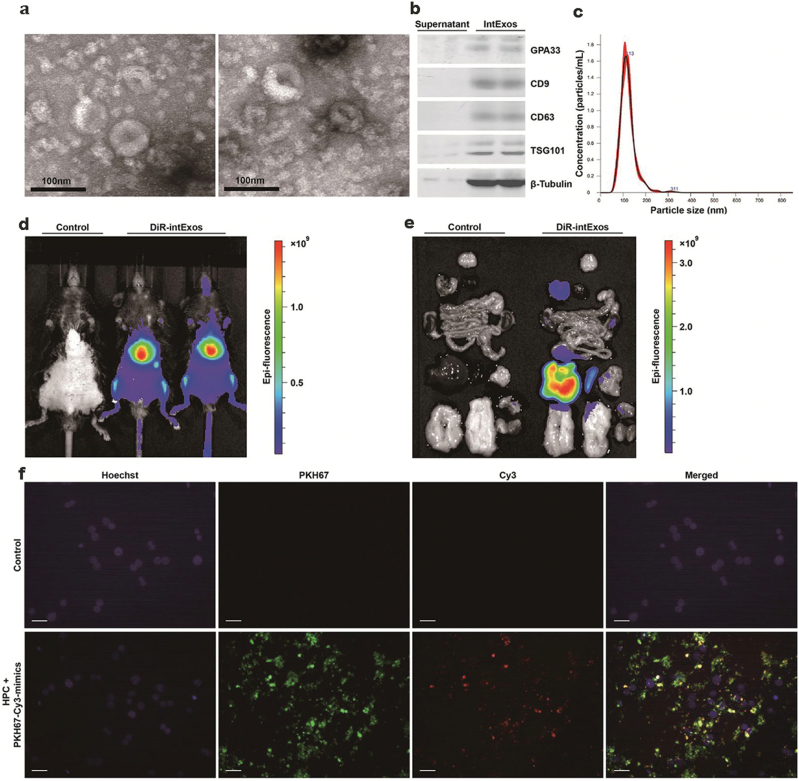
Uptake of mouse small intestinal epithelium-derived exosomes (intExos) by hepatocytes. Extracellular vesicles were isolated from mouse intestine as described in the section of Materials and methods. (a) Representative transmission electron microscopy images showing the exosomal “cup-shaped” structure of the vesicles. *n* = 5. (b) Representative results of western blotting detecting exosomal markers CD9, CD63, TSG101, and intestinal epithelial marker GPA33. *n* = 3. (c) Particle size of extracellular vesicles detected by Nanosight-nanoparticle tracking analysis. *n* = 10. (d and e) Fluorescence of DiR-labeled intExos detected by IVIS Spectrum *in vivo* imaging system in mice. *n* = 5. (f) Fluorescence of PKH67 and Cy3 detected in HPCs incubated with PKH67-labeled intExos containing Cy3-labeled microRNA mimics for 24 h. Nuclei were stained with Hoechst. *n *= 6. The scale bars are 100 μm. IntExos used in the experiments shown by this figure were isolated from 8-week-old NCD-fed C57BL/6J male mice..

Using the ultracentrifugation method described in the Materials and methods section, we isolated extracellular vesicles from mouse small intestine and validated their exosome characteristics. Extracellular vesicles visualized by transmission electron microscopy exhibited the typical “cup-shaped” structure of exosomes (Fig. 1a) and were enriched with intestinal epithelium marker glycoprotein A33 (GPA33) and exosome markers cluster of differentiation (CD) 9, CD63, tumor susceptibility gene 101 (TSG101) (Fig. 1b). NanoSight-nanoparticle tracking analysis demonstrated that the sizes of these vesicles were 50–200 nm in diameter with a peak at 113 nm (Fig. 1c), as conformed with the recognized size of exosomes. These results suggest that intExo extraction using the method mentioned above is feasible.

To test whether intExos can be taken up by metabolic organs, we labeled the isolated intExos with DiR dye and injected DiR-intExos solution into C57BL/6J mice through the tail vein. The fluorescence distribution patterns within mouse bodies were observed using Impact of Visual Impairment after Stroke (IVIS) Spectrum *in vivo* imaging system 8 h after administration of DiR-intExos (Fig. 1d). The strongest luminescence was detected in the liver (Fig. 1e). The capability of intExo uptake by hepatic primary cells (HPCs) was further confirmed *in vitro* (Fig. 1f). HPCs were incubated with PKH67-labeled intExos containing Cy3-labeled microRNA mimics. The fluorescence of PKH67 on the cell surface and Cy3 inside the cytosol of HPCs were observed. These results suggested that HPCs are able to take up intExos.

### Intestinal exosomes alleviate HFD-induced hepatic steatosis

In order to explore whether intExos are involved in the regulation of hepatic lipid metabolism, we used the intestinal epithelium-specific *Lgr4* gene knockout mice (*Villin-Lgr4*^-/-^, VL). The alleviation of hepatic steatosis in VL mice was caused by gene-edited intestinal cells alone, making these mice suitable to explore the communication mechanisms between the intestinal epithelia and the liver. VL mice demonstrated resistance to HFD-induced liver steatosis in our unpublished data. Body weight of VL mice was substantially lower than that of WT mice fed with either normal chow diet (NCD) or HFD, despite there was no difference in food intake between the two groups. Glucose tolerance and insulin sensitivity were also significantly improved in VL mice. Liver steatosis induced by HFD was significantly reduced in VL mice, evidenced by oil red O staining (Supplementary Fig. S1a) and triglyceride (TG) levels in both liver tissue (Supplementary Fig. S1b) and serum (Supplementary Fig. S1c).

**Figure 2:**
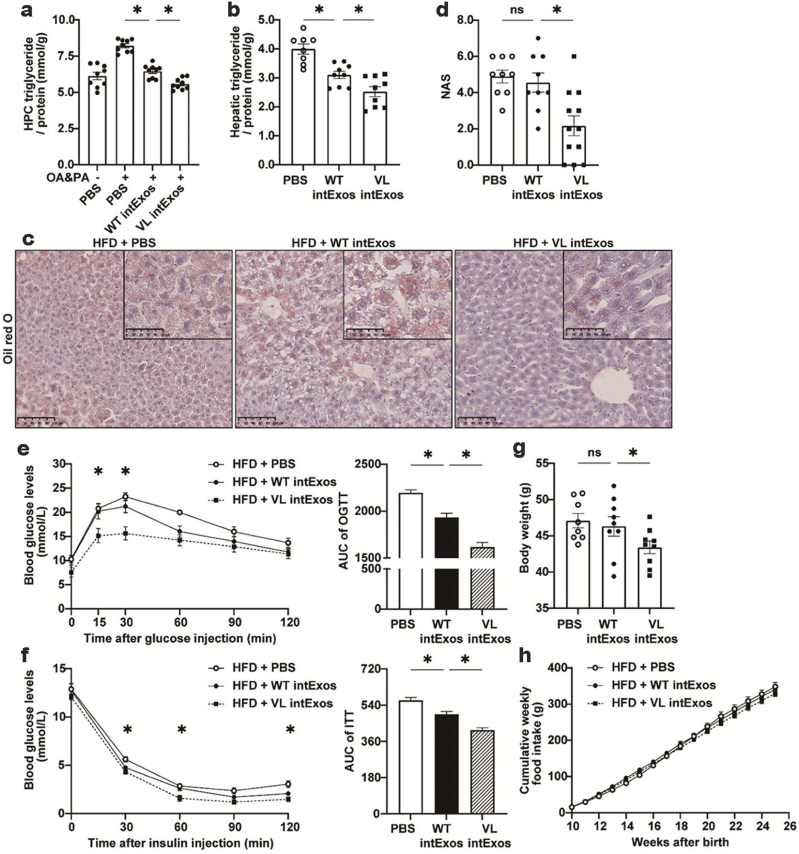
Attenuation of hepatic lipid accumulation by intestinal exosomes. (a) Reduction of TG contents in cultured HPCs. IntExos were collected from NCD-fed 8-week-old WT or VL mice. HPCs were isolated from NCD-fed male C57BL/6J mice, treated with 300 μmol/L OA and 100 μmol/L PA for 24 h, and then incubated with PBS, WT intExos, or VL intExos at a concentration of 200 ng/mL for 24 h. TG concentration was measured and normalized to protein content. Results were expressed as mean ± SEM and analyzed by one-way ANOVA. *n* = 9 for each group, * indicates *P* < 0.05. (b−h) Alleviation of liver steatosis in HFD-induced obese mice. IntExos were collected from NCD-fed 8-week-old WT or VL mice. Male C57BL/6J mice fed with HFD for 15 weeks were administered VL intExos or WT intExos at a concentration of 2 μg/g body weight through tail-vein injection, twice a week for 3 weeks before sacrifice. PBS was used as control. All values were expressed as mean ± SEM and analyzed by one-way ANOVA. * indicates *P* < 0.05, *n* = 8 mice for PBS group, 9 mice for WT intExo group, and 9 mice for VL intExo group. (b) TG concentration in the liver. (c) Oil red O staining. (d) NAS. (e) Oral glucose tolerance test (OGTT). (f) Insulin tolerance test (ITT). (g) Body weight. (h) Cumulative food intake.

We first examined the effect of VL intExos on the lipid contents of cultured HPCs. After treated with oleic acid (OA, 300 μmol/L) and palmitic acid (PA, 100 μmol/L) to induce steatosis, HPCs were incubated with intExos isolated from WT or VL mice. As shown in Fig. 2a, VL intExos significantly reduced HPC TG levels compared to WT intExos.

Next, the impact of intExos on hepatic lipid metabolism was examined in HFD-induced obese mouse model. C57BL/6J mice fed with HFD for 15 weeks from 8 weeks of age were randomized into 3 groups, and treated with PBS, WT intExos, or VL intExos through the tail vein. Compared with the PBS control group, VL intExos significantly reduced hepatic TG levels (Fig. 2b), the numbers and sizes of lipid droplets (Fig. 2c), and lowered the NAFLD activity score (NAS) (Fig. 2d), indicating the improvement in liver steatosis. Interestingly, a majority of genes related to lipid metabolism remained unchanged (Supplementary Fig. S2a–f) except for cluster of differentiation 36 (*Cd36*) and fatty acid transport protein 1 (*Fatp1*/*Slc27a1*) (Supplementary Fig. S2c). Associated with the improvement in hepatic lipid metabolism was the improvement of glucose tolerance (Fig. 2e) and insulin sensitivity (Fig. 2f), as well as reduction of body weight (Fig. 2g) in VL intExos-treated mice, whereas negligible changes of cumulative food intake between the groups were observed (Fig. 2h). WT intExos also reduced hepatic TG accumulation (Fig. 2b) and improved glucose tolerance (Fig. 2e), while demonstrating no significant effect on body weight (Fig. 2g).

### Differential regulation of hepatic lipid metabolism by miR-21a-5p and miR-145a-5p in intExos

#### Identification of miR-21a-5p and miR-145a-5p as the critical molecules mediating the effect of intExos on hepatic lipid metabolism

Subsequently, we aimed to uncover the functioning substances inside intExos that affect hepatic lipid homeostasis. Among the essential components of exosomal cargoes, we chose to examine microRNAs because there is increasing evidence indicating their participation in lipid metabolism and inflammatory signaling in NAFLD.

**Figure 3:**
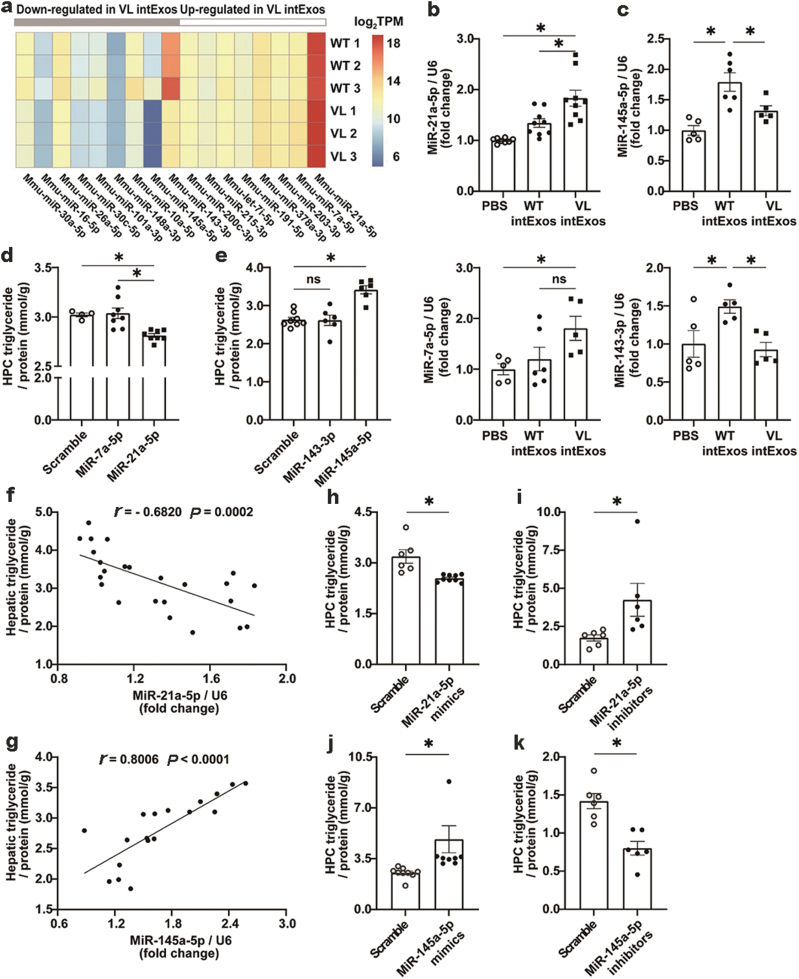
Differential regulation of hepatic lipid metabolism by miR-21a-5p and miR-145a-5p in intExos. (a) Differentially expressed microRNAs in WT and VL intExos were shown in a heatmap that reflected their transcripts per kilobase per million mapped reads (TPM) values in each sample. There were 3 samples in each group, and each intExo sample was collected from 2 NCD-fed 8-week-old mice. (b and c) IntExos were collected from NCD-fed 8-week-old WT or VL mice. Male C57BL/6J mice fed with HFD for 15 weeks from 8 weeks of age were randomly grouped and treated with PBS, WT intExos, or VL intExos. Levels of hepatic (b) miR-21a-5p (above) and miR-7a-5p (below), (c) miR-145a-5p (above) and miR-143-3p (below) were determined by RT-qPCR and normalized by the expressions of U6. Data were expressed as mean ± SEM analyzed by GraphPad Prism. *n *= 8 mice for the PBS group, 9 mice for WT intExo group, and 9 mice for VL intExo group. * denotes *P* < 0.05 by one-way ANOVA. (d and e) HPCs isolated from NCD-fed C57BL/6J mice were treated with 300 μmol/L OA and 100 μmol/L PA for 24 h. Then they were transfected with microRNA mimics of miR-21a-5p, miR-7a-5p, miR-145a-5p, or miR-143-3p. TG levels of HPCs were examined. (d) TG concentration of HPCs transfected with scramble sequence (as negative control), miR-21a-5p mimics, or miR-7a-5p mimics. (e) TG concentration of HPCs transfected with scramble sequence (as negative control), miR-145a-5p mimics, or miR-143-3p mimics. Data were expressed as mean ± SEM analyzed by GraphPad Prism. *n *= 4 − 8. * denotes *P* < 0.05 by one-way ANOVA. (f and g) IntExos were collected from NCD-fed 8-week-old WT or VL mice. Male C57BL/6J mice fed with HFD for 15 weeks from 8 weeks of age were randomly grouped and treated with PBS control, WT intExos, or VL intExos, and their liver microRNA and TG concentrations were detected. (f) The correlation between hepatic miR-21a-5p levels and TG concentration, and (g) the correlation between hepatic miR-145a-5p levels and TG concentration were calculated. Data were expressed as mean ± SEM analyzed by GraphPad Prism. *n* = 8 mice for PBS group, 9 mice for WT intExo group, and 9 mice for VL intExo group. * denotes *P* < 0.05 by one-way ANOVA. (h–k) HPCs isolated from NCD-fed C57BL/6J mice were treated with (h) miR-21a-5p mimics, (i) miR-21a-5p inhibitors, (j) miR-145a-5p mimics, or (k) miR-145a-5p inhibitors for 24 h, and harvested for TG and cholesterol detection. Data were expressed as mean ± SEM analyzed by GraphPad Prism. *n* = 6 − 9, * denotes *P* < 0.05 by Student’s *t*-test.

We performed small RNA sequencing on WT and VL intExos (Complete sequencing results are provided in Supplementary Table S1) and obtained a list of microRNAs that were differentially expressed in the two groups (Fig. 3a). First, we examined the hepatic expression levels of these microRNAs in HFD-fed mice treated with WT or VL intExos, to identify sequences whose levels altered upon intExo administration. MiR-21a-5p and miR-7a-5p were up-regulated in VL intExos-treated group compared with WT intExos-treated group (Fig. 3b). MiR-143-3p and miR-145a-5p were down-regulated in VL group compared with WT group (Fig. 3c). No statistical differences were detected between the expression levels of other microRNAs in WT and VL intExos-treated mice (Supplementary Fig. S2g–s). Therefore, only the four intExo microRNAs with significant changes in liver expression were subjected to further investigation.

We overexpressed these four microRNAs in HPCs respectively, to investigate whether they affected liver lipid metabolism. Compared with the control group, elevated expression of miR-7a-5p or miR-143-3p did not change cellular TG levels (Fig. 3d and e), while miR-21a-5p significantly reduced the lipid content of HPCs (Fig. 3d), and miR-145a-5p substantially increased cellular lipid levels (Fig. 3e). Thus, we focused our study on miR-21a-5p and miR-145a-5p in the subsequent experiments.

We found that hepatic miR-21a-5p concentrations were negatively associated with liver TG content in HFD-fed mice treated with WT or VL intExos (Fig. 3f), whereas the hepatic levels of miR-145a-5p were positively associated with liver TG concentration (Fig. 3g). These results indicate that intExos can affect the hepatic levels of miR-21a-5p and miR-145a-5p, which may lead to subsequent alteration in liver lipid metabolism.

Next, we tested whether miR-21a-5p and miR-145a-5p influence lipid accumulation in cultured HPCs via transfection of microRNA mimics or inhibitors. Relevant to the control group, HPC TG levels were significantly reduced by miR-21a-5p overexpression (Fig. 3h) but increased by miR-21a-5p inhibition (Fig. 3i). On the contrary, HPC TG concentrations were substantially increased by miR-145a-5p overexpression (Fig. 3j) whereas decreased by miR-145a-5p inhibition (Fig. 3k).

#### Amelioration of NAFLD by miR-21a-5p

To further determine whether miR-21a-5p improves hepatic lipid metabolism in HFD-induced obese mice, we overexpressed it in mouse liver using two carriers: adeno-associated virus serotype 9 (AAV9) and intExos.

##### AAV9-TBG-miR-21a-5p

First, AAV9 expressing a TBG promoter and miR-21a-5p was injected into HFD-fed mice through the tail vein and maintained for over 6 weeks to ensure adequate microRNA expression. The fluorescent protein ZsGreen was expressed alongside the microRNAs, allowing examination and validation of the liver-specific expression (Supplementary Fig. S3). ZsGreen was highly expressed only in the liver of mice treated with AAV9-carried genes. There was no obvious difference in the fluorescence intensity in subcutaneous white adipose tissue (sWAT) or epididymal white adipose tissue (eWAT) between mice treated with or without AAV9-carried genes. These observations demonstrated the liver-specific expression of AAV9-carried genes.

**Figure 4:**
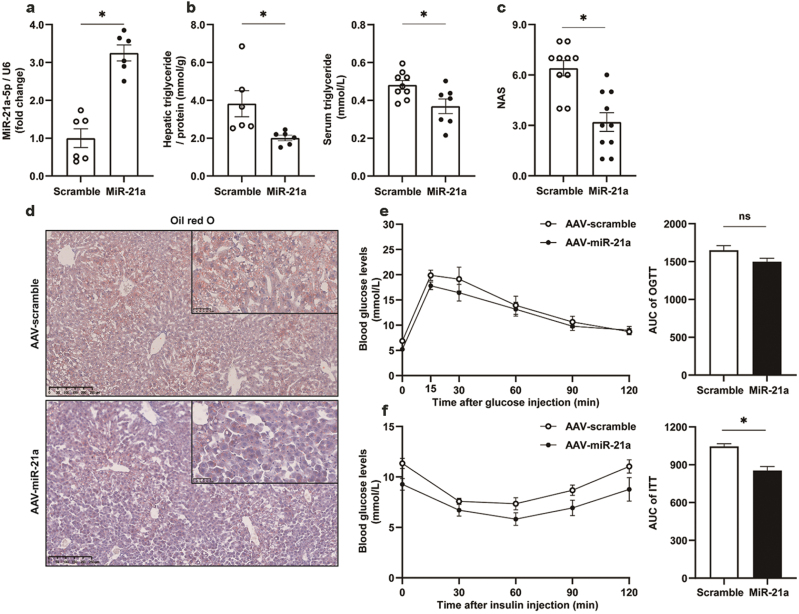
Improvement of liver lipid metabolism by AAV9-TBG-miR-21a-5p. Male C57BL/6J mice were fed with HFD for 15 weeks from 8 weeks of age, and then injected via tail vein with AAV9 virus expressing miR-21a-5p driven by the TBG promoter. AAV9-TBG expressing scramble sequences were used as control. The cohorts were maintained for at least 6 weeks before sacrifice to ensure adequate microRNA expression. All values are expressed as mean ± SEM. * indicates *P* < 0.05 analyzed by Student’s *t*-test, *n* = 7 and 9 mice for the AAV-scramble group and AAV-miR-21a group, respectively. (a) Liver concentration of miR-21a-5p. (b) Liver and serum concentrations of TG. (c) NAS. (d) Liver sections stained with Oil red O dye. (e) OGTT result and its area under the curve. (f) ITT result and its area under the curve.

Mice with liver-specific overexpression of miR-21a-5p (Fig. 4a) demonstrated a significant improvement in lipid metabolism. TG concentrations in the liver and circulation were significantly reduced (Fig. 4b). Consistently, NAS (Fig. 4d) and lipid deposition revealed by oil red O staining (Fig. 4c) were substantially decreased. No statistical difference was observed for body weight (Supplementary Fig. S4a), food intake (Supplementary Fig. S4b), or liver weight (Supplementary Fig. S4c). Glucose tolerance was slightly improved (Fig. 4e). Insulin sensitivity was significantly enhanced (Fig. 4f). mRNA levels of lipid metabolism-associated genes in the liver remained largely unaltered (Supplementary Fig. S4 d, e, g–i) except for *Cd36* and fatty acid binding protein 7 (*Fabp7*), which were substantially reduced (Supplementary Fig. S4f).

##### IntExo/miR-21a-5p

**Figure 5:**
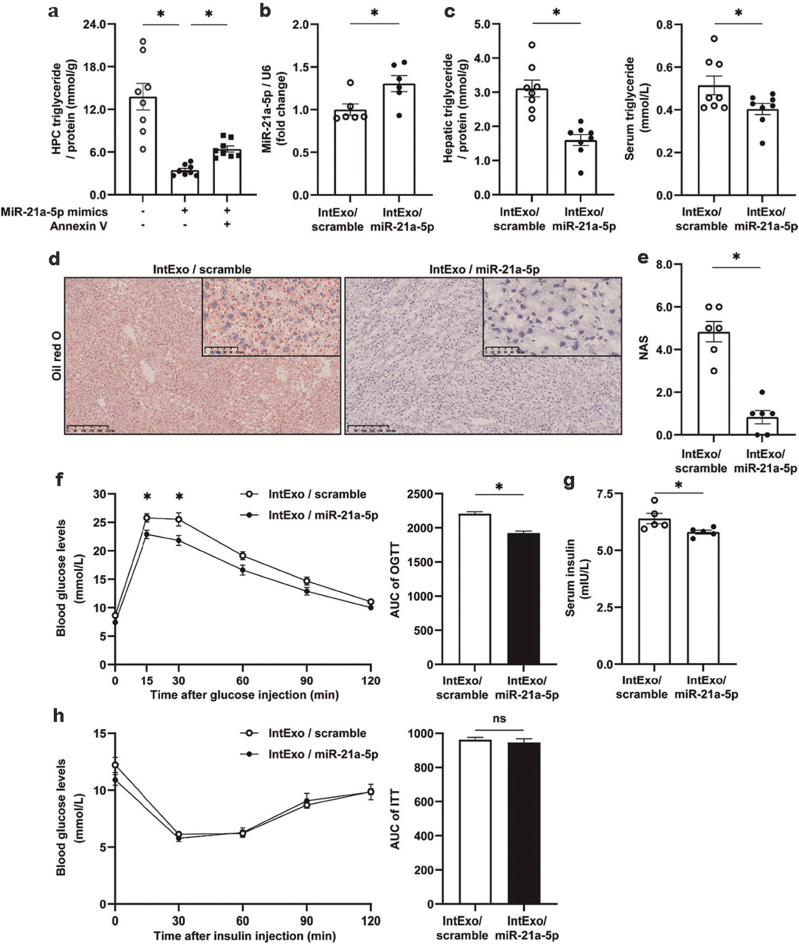
Improvement in hepatic lipid metabolism by intExo/miR-21a-5p. (a) IntExos were collected from NCD-fed 8-week-old male C57BL/6J mice, and transfected with scramble sequences (intExo/scramble) or miR-21a-5p mimics (intExo/miR-21a-5p). Some intExo/miR-21a-5p were incubated with Annexin V (2 μg/mL) for 2 h. HPCs were isolated from NCD-fed male C57BL/6J mice, and pretreated with 300 μmol/L OA and 100 μmol/L PA for 24 h. Then the HPCs were incubated with intExo/scramble, intExo/miR-21a-5p, or Anx-intExo/miR-21a-5p for 24 h. TG concentrations of HPCs were detected. (b–h) Male C57BL/6J mice were fed with HFD for 7 weeks from 8 weeks of age, and then administrated intExo/scramble or intExo/miR-21a-5p via tail-vein injection at a concentration of 2 μg/g body weight twice a week for 8 consecutive weeks. The cohorts were maintained for at least 8 weeks before sacrifice. All values are expressed as mean ± SEM and analyzed by Student’s *t*-test or two-way ANOVA. ^*^ indicates *P* < 0.05, *n* = 8 mice for the intExo/scramble group, *n* = 8 mice for the intExo/miR-21a-5p group. (b) Liver concentration of miR-21a-5p. (c) Liver and serum concentrations of TG. (d) Liver sections stained with Oil red O dye. (e) NAS. (f) OGTT result and its area under the curve. (g) Serum insulin. (h) ITT result and its area under the curve.

Second, intExos transfected with miR-21a-5p mimics (intExo/miR-21a-5p) were used to treat liver cell steatosis both *in vitro* and *in vivo*. HPCs pretreated by OA and PA were incubated with intExo/scramble, intExo/miR-21a-5p, or Anx-intExo/miR-21a-5p (Anx-intExo/ miR-21a-5p refers to intExo/miR-21a-5p that were treated with Annexin V, a protein that blocks exosome-cell membrane fusion). IntExo/miR-21a-5p substantially reduced the TG contents in cultured HPCs, while such effects were significantly attenuated by Anx-intExo/ miR-21a-5p (Fig. 5a). These results indicated the capacity of microRNA overexpression in liver cells by mimic-bearing intExos.

Then, we administrated intExo/miR-21a-5p via tail vein injection into HFD-induced obese mice and observed a substantial increase of miR-21a-5p levels in the liver (Fig. 5b). Increment of miR-21a-5p alleviated hepatic steatosis evidenced by decrement in liver and serum TG contents (Fig. 5c), lipid deposition measured by oil red O staining (Fig. 5d), as well as NAS (Fig. 5e). mRNA levels of lipid metabolism-associated genes remained largely unchanged (Supplementary Fig. S5d, e, g–i) with the exceptions of *Cd36* and *Fabp7* (Supplementary Fig. S5f). These alterations were associated with decreased body weight (Supplementary Fig. S5a), improvement in glucose tolerance (Fig. 5f), and reduction of circulating insulin (Fig. 5g). Insulin sensitivity (Fig. 5h), cumulative food intake (Supplementary Fig. S5b), and liver weight (Supplementary Fig. S5c) remained unchanged.

#### Exacerbation of NAFLD by miR-145a-5p

**Figure 6:**
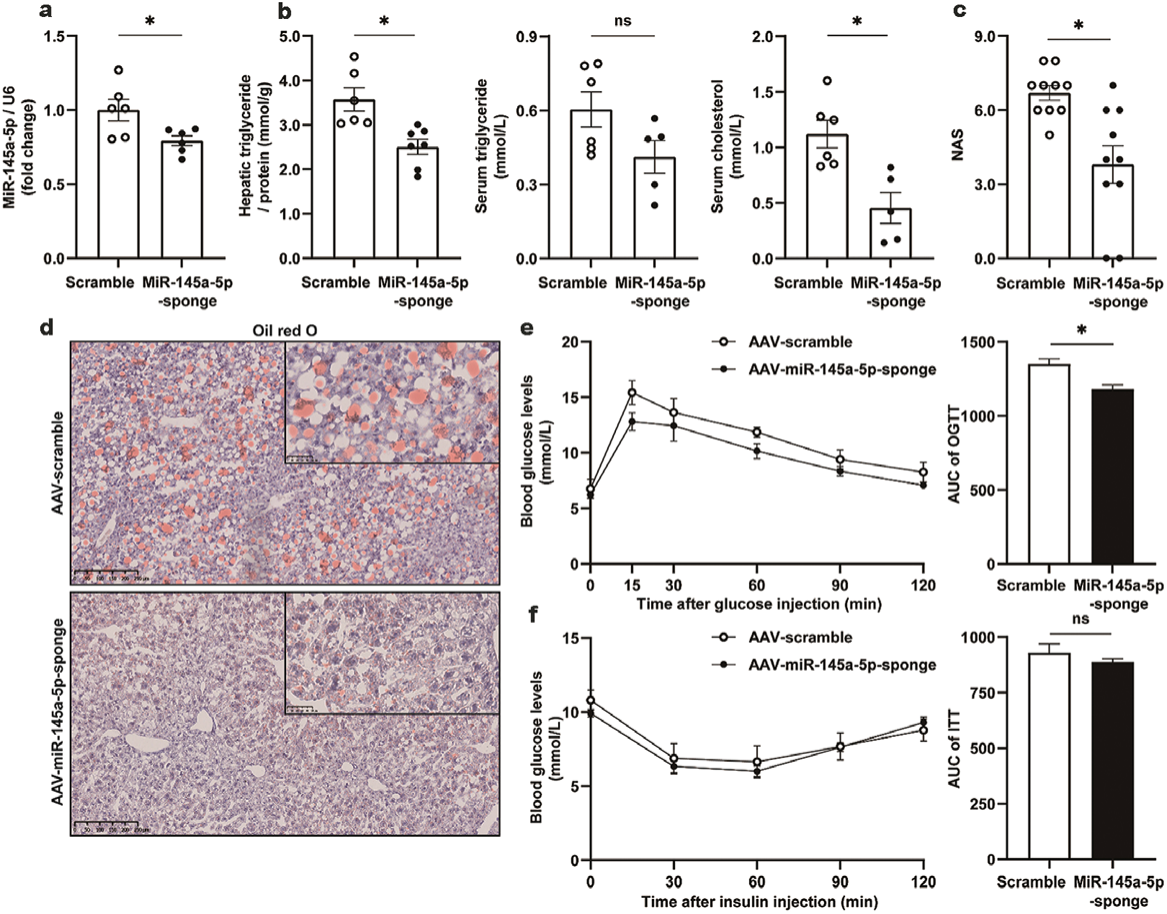
Amelioration of liver steatosis by suppression of hepatic miR-145a-5p. Male C57BL/6J mice were fed with HFD for 14 weeks from 8 weeks of age, and then injected via tail vein with AAV9 virus expressing miR-145a-5p-sponge driven by the TBG promoter. AAV9-TBG expressing scramble sequences were used as control. The cohorts were maintained for at least 6 weeks before sacrifice to ensure adequate microRNA-sponge expression. All values are expressed as mean ± SEM and analyzed by Student’s *t*-test. * indicates *P* < 0.05, *n* = 6 mice for the AAV9-scramble group, *n* = 7 mice for the AAV9-miR-145a-5p-sponge group. (a) Liver concentration of miR-145a-5p. (b) Liver concentrations of TG, and serum concentrations of TG and cholesterol. (c) NAS. (d) Liver sections stained with oil red O dye. (e) OGTT result and its area under the curve. (f) ITT result and its area under the curve.

To examine the effects of miR-145a-5p on hepatic lipid metabolism, we induced liver-specific miR-145a-5p inhibition in HFD-fed mice using AAV9-TBG-miR-145a-5p-sponge (Fig. 6a). Down-regulation of hepatic miR-145a-5p levels demonstrated a significant reduction in liver TG contents, circulating levels of cholesterol (Fig. 6b), NAS (Fig. 6c), and lipid deposition evidenced by oil red O staining (Fig. 6d). These observations indicate that suppression of hepatic miR-145a-5p renders the obese mice resistant to liver steatosis. The reduction in liver steatosis was associated with an improvement in glucose tolerance (Fig. 6e) and decreased liver weight (Supplementary Fig. S6c). No statistical difference was observed for body weight (Supplementary Fig. S6a), food intake (Supplementary Fig. S6b), and insulin sensitivity (Fig. 6f). Further, no distinguishable alteration in the mRNA levels of hepatic genes related to lipid metabolism (Supplementary Fig. S6d–i) and inflammation (Supplementary Fig. S6j) was detected.

### MicroRNA target genes mediating the regulation of liver lipid metabolism

To elucidate the mechanism by which miR-21a-5p and miR-145a-5p modulate liver lipid metabolism, we used three microRNA target gene prediction databases miRDB, TargetScan, and miRWalk to predict potential target genes of miR-21a-5p and miR-145a-5p. Then, we screened for genes that were commonly predicted by all three databases and function relevant to lipid metabolism.

*Ccl1* (C-C motif chemokine ligand 1) was chosen as the potential downstream target gene for miR-21a-5p because it was the sole result of the intersection of predicted gene lists from the three databases. On the other hand, *Btg1* (BTG anti-proliferation factor 1) and *Ext1* (Exostosin 1) were commonly predicted as the potential target gene of miR-145a-5p by all three databases. We focused our study on *Btg1* because it is the upstream regulator of stearoyl-coenzyme A (CoA) desaturase-1, a key enzyme in fatty acid metabolism.

#### CCL1-dependent mechanism by which miR-21a-5p regulates hepatic lipid metabolism

**Figure 7:**
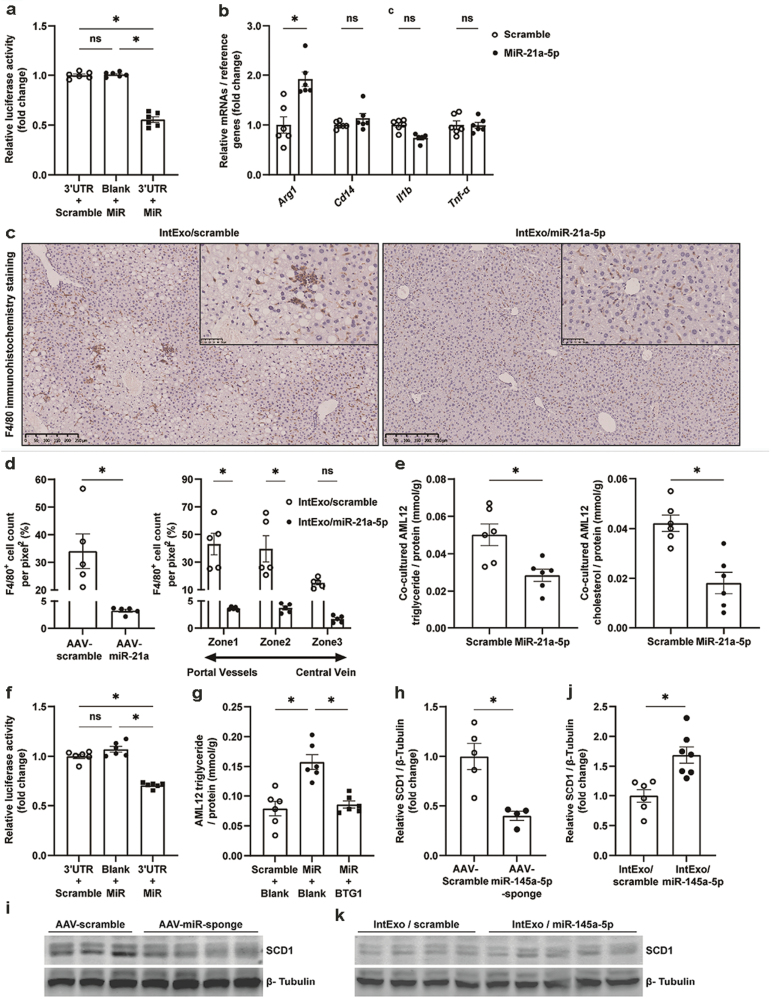
miR-21a-5p and miR-145a-5p regulate liver lipid metabolism through direct suppression of *Ccl1* and *Btg1*, respectively. (a–e) CCL1-dependent mechanism by which miR-21a-5p regulates liver lipid metabolism. (a) Dual-luciferase assay was performed to validate the direct interaction between miR-21a-5p and 3ʹ-UTR of *Ccl1* gene. (b) Raw264.7 cells were transfected with miR-21a-5p mimics or scramble sequences, and the mRNA levels of inflammation marker genes were analyzed by RT-qPCR. (c and d) Representative F4/80 staining of liver section and numbers of F4/80^+^ cells in HFD-induced obese mice injected with AAV9-miR-21a-5p or AAV-9 scramble. (e) Cellular TG and cholesterol in AML12 cells co-cultured with miR-21a-5p-treated Raw264.7 cells. Results were expressed as mean ± SEM and analyzed by one-way or two-way ANOVA, or Student’s *t*-test, *n* = 5 − 7, * denotes *P* < 0.05. (f–k) MiR-145a-5p regulates liver lipid metabolism through direct suppression of *Btg1*. (f) Dual-luciferase assay was performed to validate the direct interaction between miR-145a-5p and 3ʹ-UTR of *Btg1* gene. (g) TG concentration of AML12 cells transfected with miR-145a-5p mimics and a plasmid expressing *Btg1*-encoding sequences without 3ʹ-UTR. Protein expression of SCD1 in livers of mice treated with AAV9-miR-145a-5p-sponge (h and i) or intExo/miR-145a-5p (j and k). Results were expressed as mean ± SEM and analyzed by one-way ANOVA or Student’s *t*-test, *n* = 4 − 8, * denotes *P* < 0.05.

To validate whether *Ccl1* is a target gene of miR-21a-5p, a dual-luciferase reporter gene assay was performed to detect the presence of miR-21a-5p binding sites in the 3ʹ-UTR of the *Ccl1* gene (Fig. 7a). The fluorescence intensity was significantly reduced in 293T cells where miR-21a-5p and the 3ʹ-UTR of *Ccl1* were co-expressed, indicating that miR-21a-5p can bind with the 3ʹ-UTR of *Ccl1* to alter its expression levels. In line with this conclusion, hepatic mRNA levels of *Ccl1* were significantly reduced by VL intExos (Supplementary Fig. S7a), AAV-miR-21a (Supplementary Fig. S7b), or intExo/miR-21a-5p mimics (Supplementary Fig. S7c).

Because CCL1 is critical for chemotaxis of macrophages, we next examined whether miR-21a-5p influences hepatic lipid metabolism through regulation of macrophages. Raw264.7 cells were transfected with miR-21a-5p mimics, and the mRNA levels of *Ccl1* (Supplementary Fig. S7d) and inflammation marker genes (Fig. 7b) were analyzed by real-time quantitative PCR (RT-qPCR). MiR-21a-5p significantly suppressed the expression of *Ccl1* (Supplementary Fig. S7d). This alteration was associated with a significant increment in the expression of arginase 1 (*Arg1*) (Fig. 7b), an M2 polarization marker, indicating that miR-21a-5p promotes M2 anti-inflammatory polarization of macrophages. Consistently, F4/80 positive macrophages in the livers were substantially decreased by overexpression of miR-21-5p (Fig. 7c and d). Furthermore, co-culture with miR-21a-5p-treated Raw264.7 significantly decreased lipid accumulation in AML12 cells (Fig. 7e).

In addition, miR-21a-5p might also alleviate liver steatosis by directly suppressing hepatocyte *Fabp7*, one of its validated target genes [17]. As a key molecule in the fatty acid uptake process, *Fabp7* was down-regulated together with *Cd36* and *Fatp1* in AML12 cells transfected with miR-21a-5p mimics (Supplementary Fig. S7e).

#### BTG1-dependent mechanism by which miR-145a-5p regulates hepatic lipid metabolism

Likewise, a dual-luciferase reporter gene assay was performed to detect the presence of miR-145a-5p binding sites in the 3ʹ-UTR of the *Btg1* gene (Fig. 7f). The fluorescence intensity was significantly reduced in 293T cells where miR-145a-5p and the 3ʹ-UTR of *Btg1* were co-expressed, indicating that *Btg1* is the target gene directly bound by miR-145a-5p. The hepatic mRNA levels of *Btg1* were significantly increased by VL intExos relevant to WT intExos (Supplementary Fig. S7g). Suppression of miR-145a-5p substantially increased, whereas its overexpression decreased the hepatic levels of *Btg1* mRNA (Supplementary Fig. S7h and i).

To determine whether BTG1 mediates the effect of miR-145a-5p on hepatic lipid metabolism, AML2 cells were transfected with miR-145a-5p mimics and a plasmid expressing *Btg1*-encoding sequence without 3ʹ-UTR. As shown in Fig. 7g, overexpression of *Btg1* attenuated the increment of cellular TG concentration induced by miR-145a-5p. Further, up-regulation of hepatic *Btg1* induced by AAV9-miR-145a-5p-sponge substantially reduced levels of stearoyl-CoA desaturase-1 (SCD1), the downstream target of *Btg1* (Fig. 7h and i). On the other hand, down-regulation of hepatic *Btg1* induced by intExo/miR-145a-5p significantly increased levels of SCD1 (Fig. 7j and k).

## Discussion

### IntExos regulate hepatic lipid metabolism

Communications between metabolic organs are critical for the organismal energy balance [18]. In addition to the classical soluble secretory factors, exosomes also serve as a mediator of inter-organ communications [19]. For example, adipose-derived exosomes have been recognized as a novel communication mechanism between adipose tissue and the liver to control hepatic gene expression [14]. By regulating the hepatic expression of fibroblast growth factor 21 (FGF21), adipose-derived exosomes are proposed as the important regulators of organismal energy metabolism. This concept was further supported by studies showing that exosomes from adipose tissue macrophages modulate insulin sensitivity and glucose metabolism by transferring microRNAs to alter the gene expression of insulin-targeted cells such as hepatocytes and myocytes [20].

Our work identified the intExos as a novel pathway for the communication between the gut and liver, whose function is critical for the regulation of hepatic lipid metabolism. This concept is supported by the following observations: (i) IntExos together with cargo microRNAs are able to be taken up by hepatocytes. (ii) IntExos function to alter liver lipid homeostasis. Incubation of intExos from NCD-fed WT mice with cultured HPCs suppressed cellular lipid accumulation. Intravenous administration of intExos from NCD-fed WT mice reduced the susceptibility to liver steatosis caused by HFD. This effect was more substantial for intExos isolated from VL mice, a transgenic mouse strain resistant to HFD-induced hepatic steatosis. Since the phenotypic changes of lipid metabolism in VL mice were induced solely by intestinal gene editing, it is feasible to explore intestine epithelia-originated factors critical for controlling liver lipid homeostasis by analyzing the differential levels of exosome components between WT and VL mice. (iii) We identified microRNAs whose levels substantially differentiated between WT and VL intExos by small RNA sequencing. MiR-21a-5p and miR-145a-5p among them were enriched in both groups of exosomes and were able to alter the liver concentrations of TG in HFD-fed mice upon tail-vein intExo administration.

### IntExo miR-21a-5p ameliorates hepatic steatosis by suppressing macrophage CCL1

MiR-21a-5p levels in VL intExos were significantly higher than those of WT intExos. HFD mice receiving VL intExos via tail-vein injection demonstrated more hepatic miR-21a-5p contents compared with mice treated with WT intExos. MiR-21a-5p levels were negatively associated with TG contents in the liver of intExos-treated HFD mice. Increment of miR-21a-5p by overexpressing this microRNA carried by either AAV9 virus or intExos in the liver ameliorated HFD-induced liver steatosis. These observations indicate that miR-21a-5p functions to mediate the suppression of lipid accumulation in the liver induced by intExos.

Consistently, previous studies have demonstrated the mitigation effect of miR-21a-5p on lipid accumulation. Zhao *et al*. have found that liver-specific knockdown of miR-21a-5p using AAV9 encoding anti-miR-21a-5p increased body weight and serum lipid concentration in mice, whereas aerobic exercise substantially up-regulated serum miR-21a-5p levels. The molecular mechanism by which miR-21a-5p improves hepatic lipid metabolism may be attributed to its direct suppression of hepatic genes relevant to lipid metabolism. miR-21a-5p improved hyperlipidemia via the inhibition of its targets FABP7, 3-hydroxy-3-methylglutaryl CoA reductase (HMGCR), acetyl CoA acetyltransferase 1 (ACAT1), and oxidized low-density lipoprotein receptor 1 (OLR1) [21]. miR-21 also decreased TG, free and total cholesterol levels in HepG2 cells treated with OA and PA. These effects can be attenuated by overexpression of HMGCR, a direct target of miR-21 [22]. Consistently, our studies also observed a significant suppression of hepatic genes relevant to lipid transport, including *Fabp7* and* Cd36*.

In contrast to these observations, miR-21a-5p has also been reported to promote hepatic lipid accumulation. miR-21a-5p expression increased in the liver of NAFLD patients compared with healthy controls, whereas the change in its serum levels was contradictory in different researches [22, 23]. Transgenic mice with constitutive or liver-specific miR-21a-5p knockout demonstrated resistance to excessive steatosis and glucose intolerance when challenged with 4-week HFD [24]. HFD-fed mice injected with microRNA-21-anti-sense oligonucleotide (ASO) also exhibited decreased liver lipid storage compared with that of the control group [25]. Similarly, in a complementary fast food diet model that mimics most metabolic features of human non-alcoholic steatohepatitis (NASH) patients, miR-21a-5p knockout mice were protected from the metabolic syndrome [26]. Moreover, tail-vein injection of antagomir‑21 effectively reduced serum lipid and transaminase levels in C57BL/6J mice fed with methionine‑choline‑deficient diet (MCD) for 19 weeks [27]. The promotion of lipid accumulation in hepatocytes induced by miR-21a-5p occurs through the suppression of its downstream target, peroxisome proliferation-activator receptor α (PPARα). As the expression of miR-21 increased in the livers of both NASH/NAFLD patients and mouse models, hepatic PPARα levels decreased. By contrast, suppression of miR-21 by antagomir-21 treatment or gene knockout alleviated liver inflammation and fibrosis via restoring PPARα expression [28]. Discrepancies between the impact of miR-21 on lipid metabolism obtained from different studies remain unknown but may be attributed to the distinct effect in different target cell types.

Moreover, our studies suggest that miR-21a-5p may directly act on macrophage to inhibit the expression of *Ccl1*, a chemokine that governs immune cell infiltration upon liver injury, and therefore promotes hepatic inflammation and fibrosis [29]. Increment of miR-21a-5p by intExos reduced hepatic levels of *Ccl1*. As a result, the macrophage number in the liver was significantly decreased. Thus, this CCL1-dependent alteration in macrophage accumulation may account for the amelioration of liver steatosis induced by miR-21a-5p.

### IntExo miR-145a-5p exacerbates hepatocyte lipid accumulation via suppressing BTG1

Our work suggests that intExo miR-145a-5p worsens hepatic lipid metabolism. Deficiency of *Lgr4* in intestinal epithelia reduced levels of miR-145a-5p in intExos. Hepatocytes were able to uptake this microRNA via intExos. Levels of miR-145a-5p were positively associated with TG contents in hepatocytes. Suppression of hepatic miR-145a-5p by overexpressing its sponge decreased lipid accumulation in the liver, rendering the mice resistant to liver steatosis induced by HFD. Further, we identified *Btg1* as the downstream target of miR-145a-5p. This microRNA suppressed the expression of *Btg1* by binding to its 3ʹ-UTR, leading to an increment of SCD1 and subsequent lipogenesis. Thus, we have defined the miR-145-5p-Btg1-SCD1 pathway mediating the regulation of hepatic lipid metabolism by intExos.

Consistently, previous studies have suggested an association between miR-145 and metabolic syndromes. In type 2 diabetes mellitus (T2DM) patients, serum miR-145-5p levels were lower compared with those of the controls, and exhibited significant negative correlation with fasting blood sugar, as well as hemoglobin A1c (HbA1c) and cholesterol concentration [30]. The miR-145 levels of peripheral blood mononuclear cells from T2DM patients were also significantly lower compared with healthy controls. A constitutive suppression of miR-145 by ASO exacerbated monocyte infiltration in the liver, while systematic overexpression of miR-145 by lentivirus alleviated obesity, inflammation, and insulin resistance in *db/db* mice, and moderated atherosclerosis in *ApoE*^*−/−*^ mice [31].

Recently, two studies showed that miR-145a overexpression ameliorated liver inflammation and improved glucose metabolism in mice [31]. MiR-145a has been shown to inhibit lipid synthesis and accumulation in adipocytes (including SCD1 expression) and is negatively correlated with obesity [32]. Reasons underlying the discrepancy between previous findings and our current conclusion remain unclear, but they may be related to tissue-specific effects. Indeed, recent researches suggest that constitutive miR-145 overexpression tends to ameliorate steatosis and inflammation, whereas consequences of modifying miR-145 levels in specific tissues are heterogeneous [31, 32]. In the article, miR-145 levels were modified by intravenous injection of its ASO [31]. This global approach may not only affect miR-145 concentrations in the liver but could also impact its levels in various cell types, including macrophages. In contrast, our approach is hepatocyte-specific. In adipose tissue, miR-145 promotes adipogenesis despite that it is negatively associated with obesity [32].

Interestingly, we did not detect any effect of miR-145a-5p on *Ccl1* and inflammatory genes in macrophages (Supplementary Fig. S7j), indicating that macrophages may not mediate the effect of miR-145a-5p on lipid accumulation in the liver.

### Limitations and future directions

Limitations exist for this study. Firstly, this research focuses solely on exosomes originated from intestinal epithelium, rather than those derived from the gut microbiome, although the latter accounts for a large proportion of extracellular vesicles entering blood from the intestine [33]. Gut microbiome exosomes have been well recognized as the physiological and pathological mechanisms for the control of lipid metabolism in the liver and adipose tissue [34]. Relevant to microbial-derived exosomes, the role of intExos in controlling hepatic lipid metabolism remains unknown. This study has optimized the separation of gut contents including microbiome from intestinal epithelial cells through dithiothreitol (DTT) digestion, allowing for intestinal epithelial cell-specific exosome characterization. Together with the VL mice, this approach allows us to explore the specific effect of intestinal epithelia exosomes on hepatic lipid metabolism. However, it is worth noting that this experimental protocol still cannot guarantee the purity of exosome sources. Future investigation should focus on the study by using exosomes isolated from intestinal epithelium cell lines or germ-free mice to exclude the effect of gut microbiome exosomes.

Secondly, the VL model was adopted for research because its phenotypic changes are caused by intestinal epithelial cells, allowing us to search for the intestinal epithelia-derived factors that regulate liver lipid metabolism. Consistently, intExos of WT mice fed with NCD also alleviated hepatic steatosis, with a slightly lower efficacy than that of VL intExos. Further investigation should aim to fully characterize the effect of WT intExos on liver steatosis. In addition, in order to more accurately illustrate how exosomes and their contents influence the progression of NASH, we should consider employing the Amylin liver NASH (AMLN) diet or the Gubra-Amylin NASH diet in our future studies. These diets have been shown to induce NASH more consistently than HFD.

Thirdly, we focused our study on the microRNAs and identified miR-21a-5p and miR-145a-5p as the critical microRNAs that affect hepatic lipid metabolism by comparing the microRNA profiles and therapeutic effects of WT and VL intExos. Additional experiments were conducted to compare the microRNA expression levels in intExos from mice that were fed either NCD or HFD. The levels of miR-145-5p were up-regulated in intExos from mice fed with HFD, in comparison to those from mice fed with NCD. However, the levels of miR-21a-5p showed no significant difference between the two groups. This observation suggests that the composition of intExo microRNA profiles may vary depending on the specific experimental models employed. Moreover, exosomal cargoes other than microRNAs may also mediate the effect of intestinal epithelia exosomes on hepatic lipid metabolism. We conducted further experiments to profile the protein contents of intExos. During this investigation, we pinpointed a few protein candidates whose levels exhibited significant changes. We are currently validating the alteration of these candidate proteins in different models such as VL, diet-induced obesity, and intermittent fasting mice.

## Conclusion

Our study offers a glimpse into the regulatory process of intExos on the hepatic lipid metabolism and progress of NAFLD. By secreting exosomes containing miR-21a-5p and miR-145a-5p, intExos, especially in mice with deficiency of *Lgr4* in intestinal epithelial cells, function to alter the lipid homeostasis by concurrent suppressing *Ccl1* in macrophages and *Btg1* in hepatocytes, respectively. Therefore, targeting intestinal miR-21a-5p and miR-145a-5p may provide a new perspective for the intervention of NAFLD.

## Materials and methods

This manuscript follows the ARRIVE (Animal Research: Reporting of *In Vivo* Experiments) reporting guidelines [35].

### Animals

Six-week-old C57BL/6J male mice were purchased from the Department of Experimental Animal Science, Peking University Health Science Center. *Lgr4*^*iKO*^ mice (VL) were generated by crossing *Villin-cre* mice with *Lgr4*^*flox/flox*^ mice, wherein *Villin-cre* mice were purchased from the Jackson Laboratory and *Lgr4*^*flox/flox*^ mice were generated as described before [36]. Mice were maintained in a regulated environment (24°C, 12-h light/12-h dark cycle with lights on at 8:30 a.m.) that conforms with specific pathogen free standards. Experiments were carried out during the light cycle. NCD and water were available *ad libitum* unless specified otherwise. Eight-week-old male mice were fed with HFD (60% fat, D12492; Research Diets) or NCD for the times indicated.

Mice used in this study were all males. Because estrogen has the effect of resisting steatosis, NAFLD phenotypes such as lipid deposition are more difficult to induce in female mice than in males [37], which hinders the observation and detection of therapeutic effects.

### Isolation of intExos

C57BL/6J mice were euthanized to obtain small intestine. The peri-intestinal adipose tissue was removed, and the intestines were cut into small sections and then flushed with iced PBS. The intestinal lumen surface of each small segment was turned outward and placed in 10 mmol/L DTT/PBS to rid the mucus on the surface of the intestinal mucosa. The intestine segments were then transferred to 8 mmol/L EDTA/PBS and incubated for 30 min. After digestion, the intestine segments were carefully replaced in ice-bathed PBS and shaken vigorously for 5 min to obtain intestinal epithelial cells as described previously [38]. The dissociated intestinal epithelial cells were collected and maintained in ice-bathed PBS for 30 min to allow secretion of exosomes. The cell suspension was then centrifuged at 3000*g* for 10 min. The supernatant was aspirated, passed through a 220-μm filter, and centrifuged at 100,000*g* at 4°C for 1 h. Then the supernatant was discarded, and the extracellular vesicle-containing pellet was resuspended in ice-cold PBS and centrifuged again at 100,000*g* at 4°C for 20 min to yield the exosomes for further use.

### HPC isolation and culture

NCD-fed male C57BL/6J mice were anesthetized using 1% pentobarbital and subjected to laparotomy. After cannulation of the portal vein, the liver was perfused sequentially with 20 mL of 37°C D-Hank’s and 20 mL of 0.02% collagenase IV (Sigma-Aldrich) solution, at a flow rate of 2 mL/min. The liver was transferred into the DMEM medium after perfusion. The liver capsule was torn to disperse hepatic cells. The cell suspension was filtered through 70 μm mesh and centrifuged at 50 g twice to remove debris. Then the HPCs were suspended by DMEM complete medium supplemented with 10% fetal bovine serum (FBS) and cultured in a humidified incubator at 37°C in a 5% (vol/vol) CO_2_ atmosphere.

### Transfection of microRNA mimics or inhibitors into cells

Mmu-miR-21a-5p mimics/inhibitors, mmu-miR-145a-5p mimics/inhibitors, and negative control microRNA sequences were designed and synthesized by GenePharma (Shanghai, China), and transfected into HPCs or AML12 cells using Lipo8000 Reagent (Beyotime). Treated cells were harvested 48 or 60 h after transfection for RNA or protein detection, respectively.

### Transfection of microRNA mimics or inhibitors into intExos

MicroRNA mimics and scramble sequences as negative control were transfected into intExos using Exo-Fect™ Exosome Transfection Kit (System Biosciences).

### AAV9-TBG-microRNA/sponge-ZsGreen

AAV was purchased from Hanbio Biotechnology Co., Ltd. AAV9 expressing microRNA/sponge and ZsGreen fluorescent protein was driven by a TBG promoter for liver-specific microRNA manipulation. For liver overexpression or inhibition of certain microRNA, AAV9-TBG-microRNA-ZsGreen (2 × 10^11^ virus genome) or AAV9-TBG-miR-sponge-ZsGreen (2 × 10^11^ virus genome) was injected into HFD-fed mice through the tail vein. Mice in control groups were treated with AAV9-TBG-scramble-ZsGreen (expressing scrambled microRNA sequence, 2 × 10^11^ virus genome).

### Oral glucose tolerance test (OGTT)

After fasting for 16 h, mice were orally gavaged with glucose at a dose of 3 g/kg body weight, and glucose concentrations in blood collected from the tail tip at 15, 30, 60, 90, and 120 min after gavage were determined.

### Insulin tolerance test (ITT)

After fasted for 6 h, mice were then intraperitoneally injected with insulin at a dose of 1 U/kg body weight, and glucose concentrations in blood collected from the tip of the tail at 30, 60, 90, and 120 min after insulin administration were determined.

### Reverse transcription-quantitative real-time polymerase chain reaction

Total RNA was extracted by RNATrip from Applygen according to the provided protocol. RNA samples were reverse-transcribed using Reverse Transcription Kit (Vazyme) as previously described [39]. The quantifications of gene transcripts were performed by RT-qPCR using Agilent AriaMx Real-Time PCR System (Agilent Technologies). *Hprt*, *Tbp*, and *Rpl32* served as internal controls. A list of the PCR primers used to amplify the target genes is provided in Table 1.

**Table 1.** qPCR primers.

Genes	Forward primer（5ʹ-3ʹ）	Reverse primer（5ʹ−3ʹ）
*Hprt*	TCAGTCAACGGGGGACATAAA	GGGGCTGTACTGCTTAACCAG
*Tbp*	ACCTTATGCTCAGGGCTTGG	GCCGTAAGGCATCATTGGAC
*Rpl32*	GAGCAACAAGAAAACCAAGCA	TGCACACAAGCCATCTACTCA
*Srebf1*	GGAGCCATGGATTGCACATT	GGAAGTCACTGTCTTGGTTGTTGA
*Pparg*	TCAGCTCTGTGGACCTCTCC	ACCCTTGCATCCTTCACAAG
*Pparg2*	CTCTGGGAGATTCTCCTGTTGA	GGTGGGCCAGAATGGCATCT
*Acaca*	TGGTCGTGACTGCTCTGTGC	GTAGCCGAGGGTTCAGTTCC
*Fasn*	TGGGTTCTAGCCAGCAGAGT	ACCACCAGAGACCGTTATGC
*Scd1*	GCGATACACTCTGGTGCTCA	CCCAGGGAAACCAGGATATT
*Dgat1*	TTCCGCCTCTGGGCATT	AGAATCGGCCCACAATCCA
*Dgat2*	CGTGACGTGCATTGGCTTC	TGGAGGGCTGAGAGGATGC
*Gpam*	CACACGAGCAGGAAAGATGA	GGACTGCATAGATGCTGCAA
*Cd36*	TGGTCAAGCCAGCTAGAAA	CCCAGTCTCATTTAGCCAC
*Fabp7*	CCGAAGCTTGCACTGGTCACTAAT	GGACTAGTCCGAAGACAAAC
*Ppara*	GAGAAGTTGCAGGAGGGGATTGTG	AAGACTACCTGCTACCGAAATGGG
*Cpt1a*	ATCGTGGTGGTGGGTGTGATAT	ACGCCACTCACGATGTTCTTC
*Acadm*	TTACCGAAGAGTTGGCGTATGG	TGCGGAGGGCTCTGTCAC
*Acadl*	CTCCCTGCGCGTCCTGAG	AAAATGTCATGCTCCGAGGAAAAG
*Acadvl*	GCCCAGACACACAACCTTTG	CCGAGCCGACTGCATCTC
*Srebf2*	CCGCTCTCGAATCCTCTTAT	CAGCACCTGACTCCAGTGAC
*Hmgcr*	TGACCTTTCTAGAGCGAGTGCAT	CACGAGCTATATTTTCCCTTACTTCA
*Hmgcs2*	ACCTGCGGGCCTTGGAT	GGTGAAAGGCTGGTTGTTTCC
*Cyp7a1*	TGGGCATCTCAAGCAAACAC	TCATTGCTTCAGGGCTCCTG
*Cyp8b1*	GCCTTCAAGTATGATCGGTTCCT	GATCTTCTTGCCCGACTTGTAGA
*Abcg5*	CCAGCAGAAGTGGGACA	GCAGCCATTCACAAACA
*Abcg8*	CGCCGACGAGTGAGCAT	AGGTCAAATAGCCTGAAGAT
*Abca1*	GAGGAGCAAGGTATCGG	AAGCCATCCACAGCAAC
*Arg1*	CTCCAAGCCAAAGTCCTTAGAG	AGGAGCTGTCATTAGGGACATC
*Il10*	CTTACTGACTGGCATGAGGATCA	GCAGCTCTAGGAGCATGTGG
*Il6*	CTGCAAGAGACTTCCATCCAG	AGTGGTATAGACAGGTCTGTTGG
*Tnfa*	CATCTTCTCAAAATTCGAGTGACAA	TGGGAGTAGACAAGGTACAACCC
*Il1b*	CCGTGGACCTTCCAGGATGA	GGGAACGTCACACACCAGCA
*Cd14*	CATTTGCATCCTCCTGGTTTCTGA	GAGTGAGTTTTCCCCTTCCGTGTG

We used Mir-X^TM^ miRNA qRT-PCR TB Green® kit for microRNA qPCR. The kit includes a non-specific primer and a microRNA-specific primer with the same nucleotide sequence as the target microRNA (Mmu-miR-21a-5p: UAGCUUAUCAGACUGAUGUUGA; miR-145a-5p: GUCCAGUUUUCCCAGGAAUCCCU).

### Tissue sample preparation and immunohistochemical staining

Mice were anesthetized using pentobarbital (0.07 g/kg body weight). Tissues were quickly harvested and rinsed thoroughly with PBS, then fixed in 4% paraformaldehyde (wt/vol), dehydrated, embedded in paraffin or tissue OCT compound, and sectioned at 5 μm. The sections were used for hematoxylin and eosin (H&E), Oil Red O, or immunohistochemistry staining following general protocols.

### Cell lines

The 293T and AML12 cell lines were purchased from the American Type Culture Collection. The 293T cells grown in DMEM complete medium. The cells were cultured in a humidified incubator at 37°C in a 5% (vol/vol) CO_2_ atmosphere. The AML12 cells were planted in DMEM/F-12 medium containing 10% (vol/vol) FBS, 1% (vol/vol) ITS (Insulin-transferrin-sodium selenite media supplement), 40 ng/mL dexamethasone, 100 U/mL penicillin, 100 μg/mL streptomycin, and 2 mmol/L glutamine.

### Dual-luciferase assay

The putative binding sites in 3ʹ-UTR of *Ccl1* or *Btg1* mRNA were characterized using TargetScan database. A fragment containing the potential binding site was amplified and inserted into the pmirGLO3 vector (Synbio Technologies. Its sequence is available in Supplementary information), where it was at the downstream of the luciferase reporter gene. The pmirGLO3 vector alone (Empty) without sequence insertion was included as a positive control. 293T cells seeded in 24-well plates were con-transfected with binding site-containing vector, pRL-TK Renilla Luciferase Reporter Vector (Promega), and microRNA mimics, facilitated by Lipo8000 Reagent. Forty-eight hours after transfection, luciferase activities were measured using the Dual-Luciferase® Reporter Assay system (Promega). Firefly luciferase activity was normalized to that of renilla for each sample.

### Western blotting

Freshly isolated tissues were homogenized in the radioimmunoprecipitation assay (RIPA) buffer. Proteins were extracted, then separated by SDS-PAGE, and transferred to the polyvinylidene fluoride (PVDF) membrane, where immunoblot was performed using the specific antibodies listed below.

**Table:** 

Antibodies	Vendor	Item number
β-Tubulin mAb	Abmart	M20005
GPA33 Rabbit pAb	Abcam	Ab203286
CD9 Rabbit pAb	Abcam	Ab223052
CD63 Rabbit mAb	Abcam	Ab217345
TSG101 Rabbit pAb	ABclonal	A2216
SCD1 Rabbit pAb	ABclonal	A16429
F4/80 Rabbit mAb	Cell Signaling Technology	70076T
Goat Anti-Mouse IgG H&L	Bioss	bs-40296G-IRDye8
Goat Anti-Rabbit IgG H&L	Bioss	bs-40295G-IRDye8

### Statistical analysis

Mice were randomly divided into experimental and control groups before the beginning of AAV or exosome treatment.

All *in vitro* experiments were repeated at least twice and reproduced. RT-qPCR was performed once but at least three independent samples were analyzed. Western blotting data were confirmed by at least three independent samples. Statistical analyses were performed using GraphPad Prism 9 (GraphPad). All data are represented as Mean ± SEM. Unpaired Student’s *t*-tests were used for two-group comparisons. One-way analysis of variance (ANOVA) followed by Tukey’s test was used for multiple-group comparisons. Two-way ANOVA was used to analyze data of OGTT, ITT, body weight, cumulative food intake, and RT-qPCR. Data points identified as outliers by GraphPad Prism were excluded. The statistical parameters and mouse numbers used per experiment are specified in the figure legends. No statistical methods were used to predetermine the sample size. *P* < 0.05 denotes statistical significance.

## Supplementary Material

load044_suppl_Supplementary_Figures

load044_suppl_Supplementary_Tables

load044_suppl_Supplementary_Data

## Data Availability

All study data are included in the article and/or supplementary information. Materials and reagents are available upon request.
