# Supplementary material for: Regulation of hepatic lipid metabolism by intestine epithelium-derived exosomes
Source: Life Metab. 2023 Nov 21;2(6):load044. doi: 10.1093/lifemeta/load044 (PMC11749469; doi:10.1093/lifemeta/load044)
Supplement: load044_suppl_Supplementary_Figures [file load044_suppl_Supplementary_Figures.pdf]

Supplementary Figure S1 (Related to Figure 2) Alleviation of HFD-induced hepatic steatosis by intestinal epithelium-specific knockout of *Lgr4* mice (*Lgr4*^{iKO}, VL).

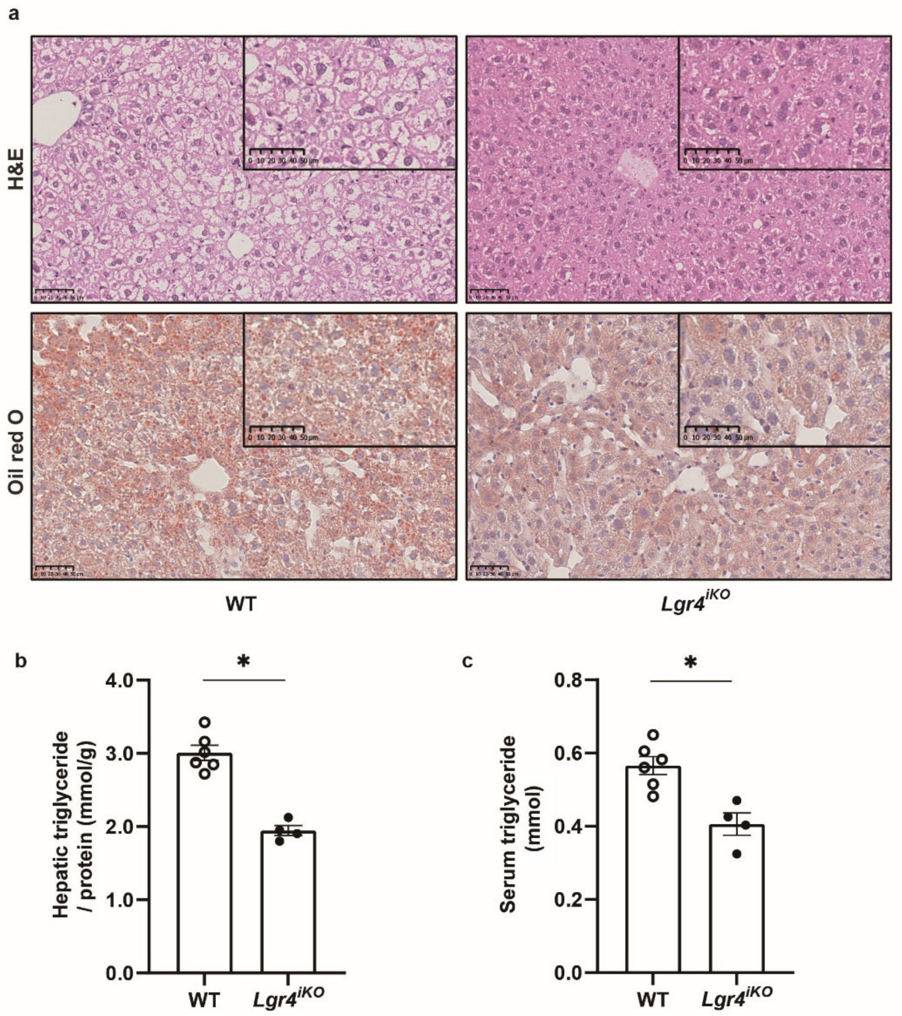

VL and the *Lgr4*$^{flox/flox}$ littermates were fed with HFD for 12 weeks from 6 weeks old. Results were expressed as mean ±SEM and analyzed by Student's *t*-test. n = 6 and 4 mice for the WT group and VL group, respectively. * denotes P <0.05. **(a)** H&E and oil red O staining of liver sections. Levels of triglyceride in liver **(b)** and serum **(c)**.

Supplementary Figure S2 (Related to Figure 2 and 3) Effects of VL intExos on the expression of genes relevant to liver lipid metabolism and on hepatic microRNA concentrations in HFD-induced obese mice.

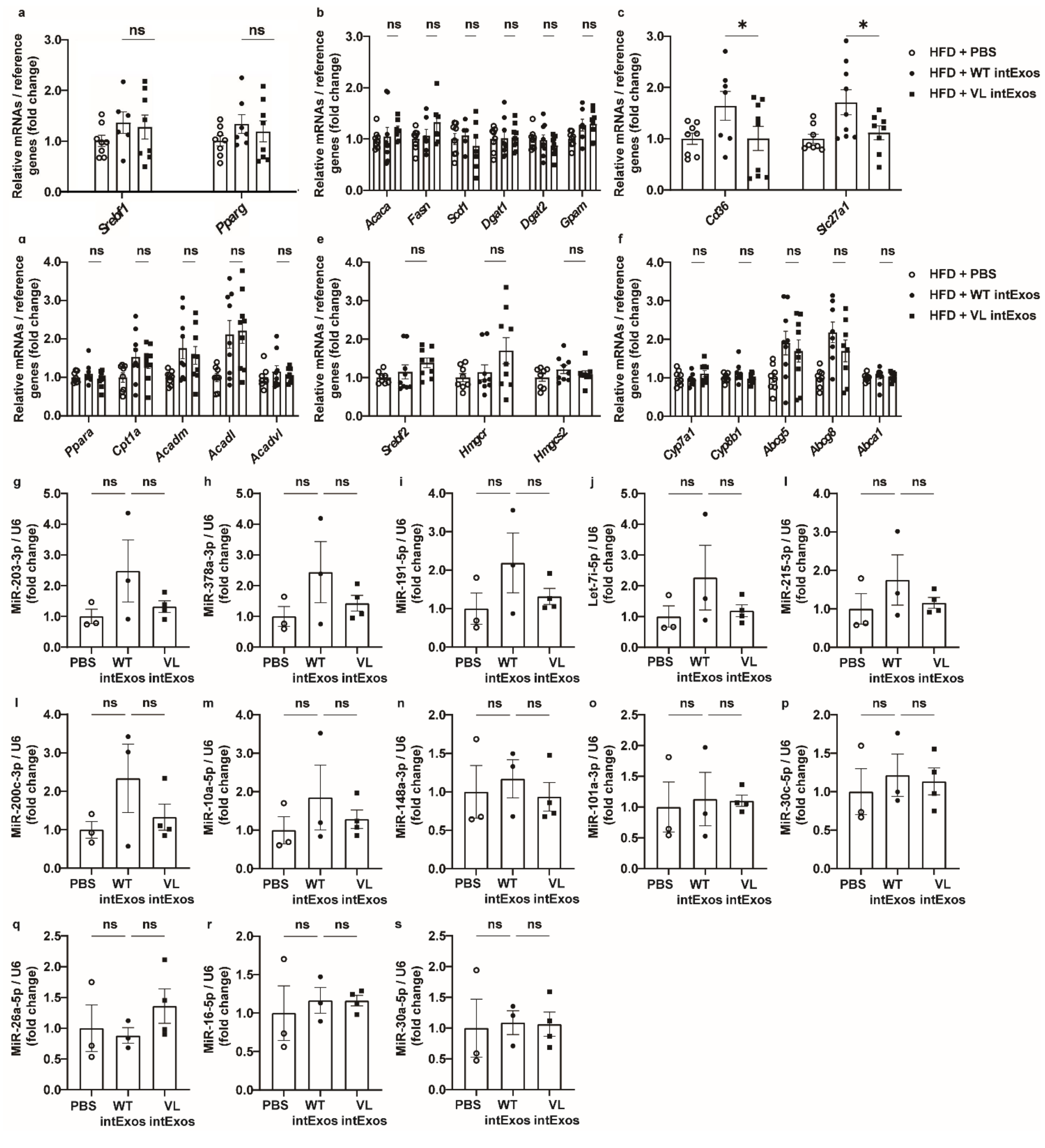

IntExos were collected from NCD-fed 8-week-old WT or VL mice. Male C57BL/6J mice fed with HFD for 15 weeks were administered VL intExos or WT intExos at a concentration of 2 μg/g body weight through tail vein injection, twice a week for 3 weeks before sacrifice. PBS was used as control. **(a−f)** are related to Figure 2. Effects of VL intExos on the expression of genes relevant to liver lipid metabolism in HFD-fed mice. Hepatic mRNA expressions of **(a)** lipogenesis-associated transcription factors and **(b)** genes, **(c)** lipid transport, **(d)** β-oxidation, **(e)** cholesterol synthesis, and **(f)** cholesterol transport-associated genes were determined by real-time quantitative PCR (RT-qPCR) and normalized by the geomeans

of *Hprt*, *Tbp*, and *Rpl32*. Results were expressed as mean±SEM and analyzed by two-way ANOVA. n = 8, 9, 9 for the PBS control, WT intExos, and VL intExos groups, respectively. * indicates $P < 0.05$. **(g–s)** are related to Figure 3. Effects of VL intExos on hepatic microRNA levels in HFD-fed mice. Hepatic microRNA levels of **(g)** miR-203-3p, **(h)** miR-378a-3p, **(i)** miR-191-5p, **(j)** let-7i-5p, **(k)** miR-215-3p, **(l)** miR-200c-3p, **(m)** miR-10a-5p, **(n)** miR-148a-3p, **(o)** miR-101a-3p, **(p)** miR-30c-5p, **(q)** miR-26a-5p, **(r)** miR-16-5p, **(s)** miR-30a-5p were determined by RT-qPCR and normalized by the expressions of U6. Results were expressed as mean±SEM and analyzed by One-way ANOVA. n = 3–4. * indicates $P < 0.05$.

Supplementary Figure S3 (Related to Figure 4 and Figure 6) Liver-specific expression of zsGreen in AAV9-TBG treated mice .

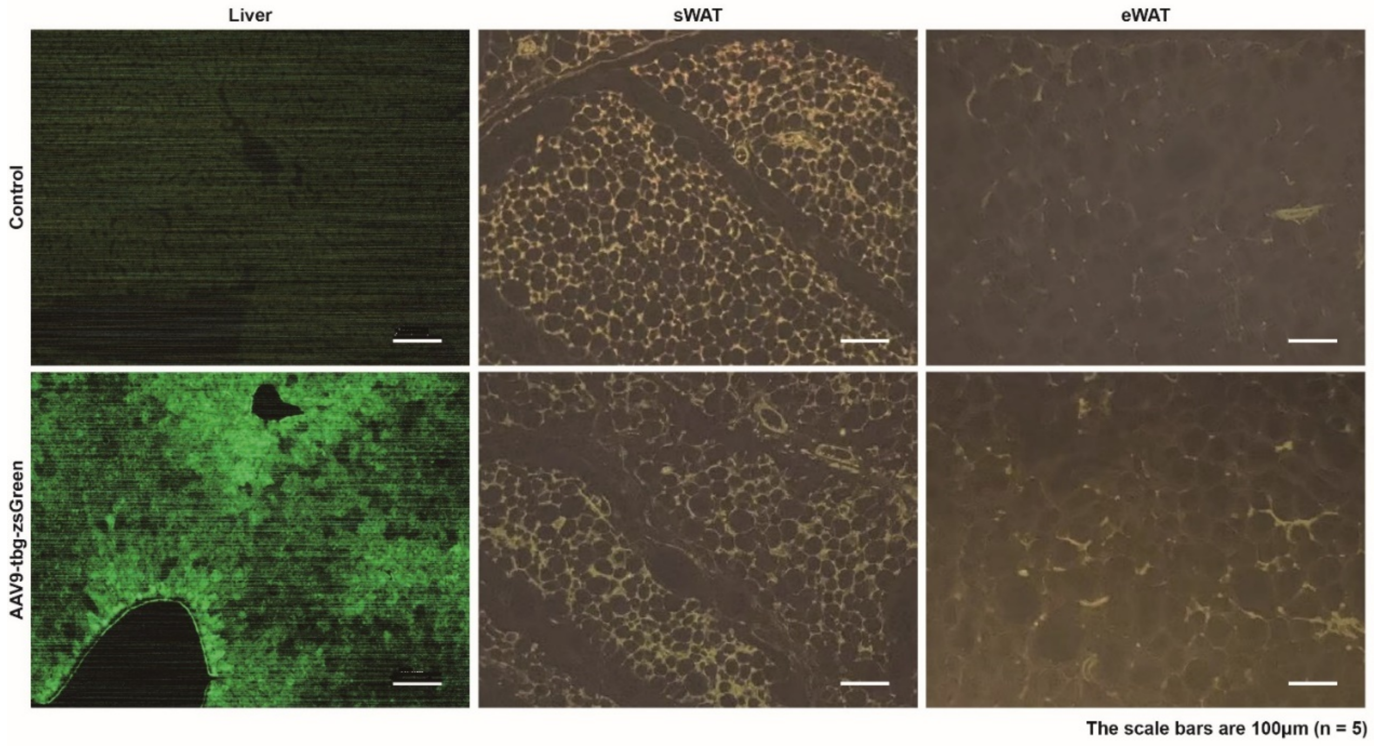

zsGreen fluorescence of tissue sections from mice injected AAV9-TBG-carried genes compared with mice without treatment. The scale bars are 100 μm. *n* = 5.

Supplementary Figure S4 (Related to Figure 4) Other metabolic effects of AAV9-TBG-miR-21a-5p.

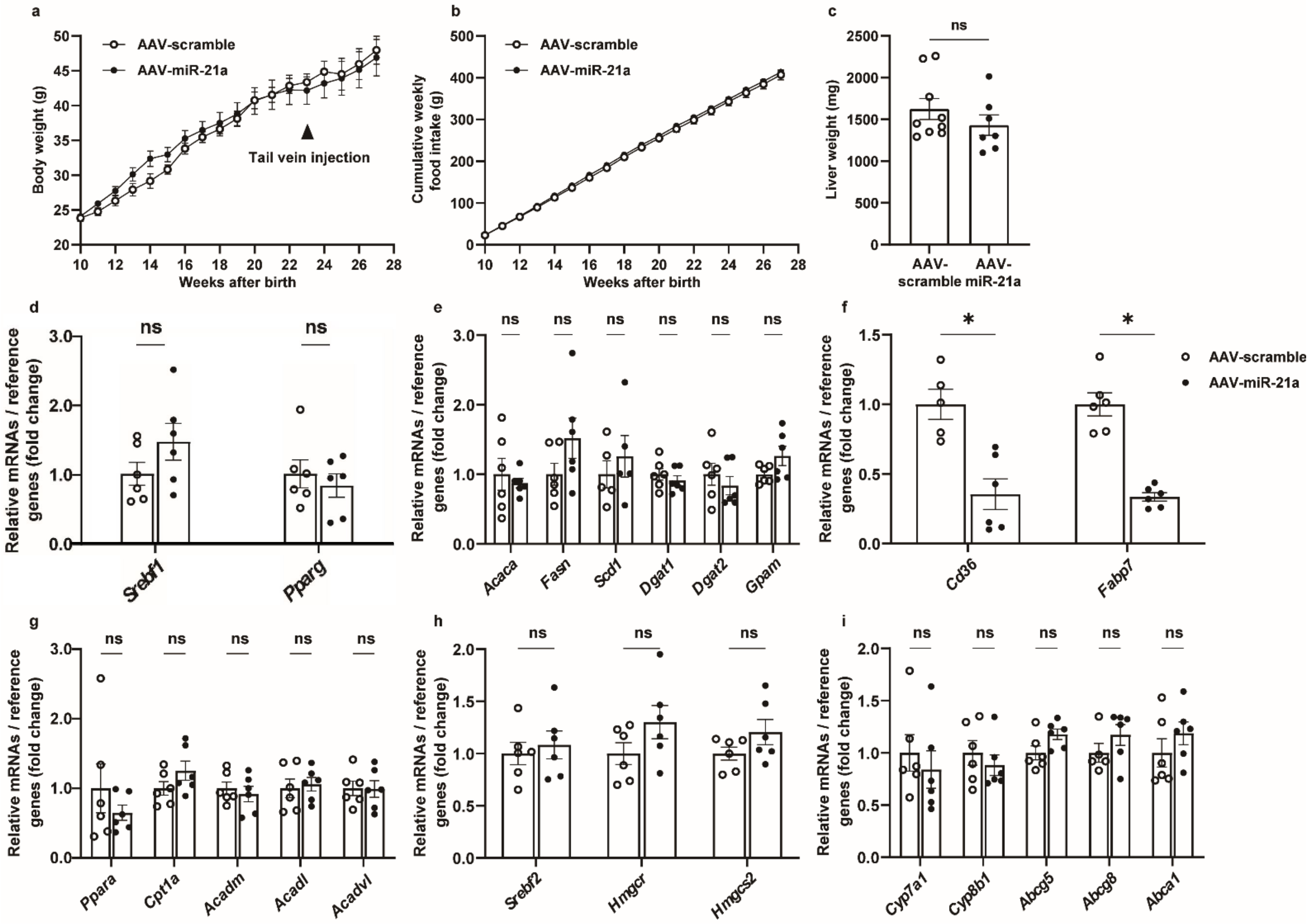

Male C57BL/6J mice were fed with HFD for 15 weeks from 8 weeks of age, and then injected via tail vein with AAV9 virus expressing miR-21a-5p driven by the TBG promoter. AAV9-TBG expressing scramble sequences were used as control. The cohorts were maintained for at least 6 weeks before sacrifice to ensure adequate microRNA expression. **(a)** Body weight. **(b)** Food intake. **(c)** Liver weight, eWAT weight, and sWAT weight. mRNA expression of (**d**) lipogenesis-associated transcription factors and **(e)** genes, **(f)** lipid transport, **(g)** β-oxidation, **(h)** cholesterol synthesis, and **(i)** cholesterol transport-associated genes were determined by RT-qPCR and normalized by the geomeans of *Hprt*, *Tbp*, and *Rpl32*. All values are expressed as mean±SEM. * denotes p<0.05 by two-way ANOVA or Student's t-test, n = 6 for each group.

Supplementary Figure S5 (Related to Figure 5) Other metabolic effects of intExo/miR-21a-5p.

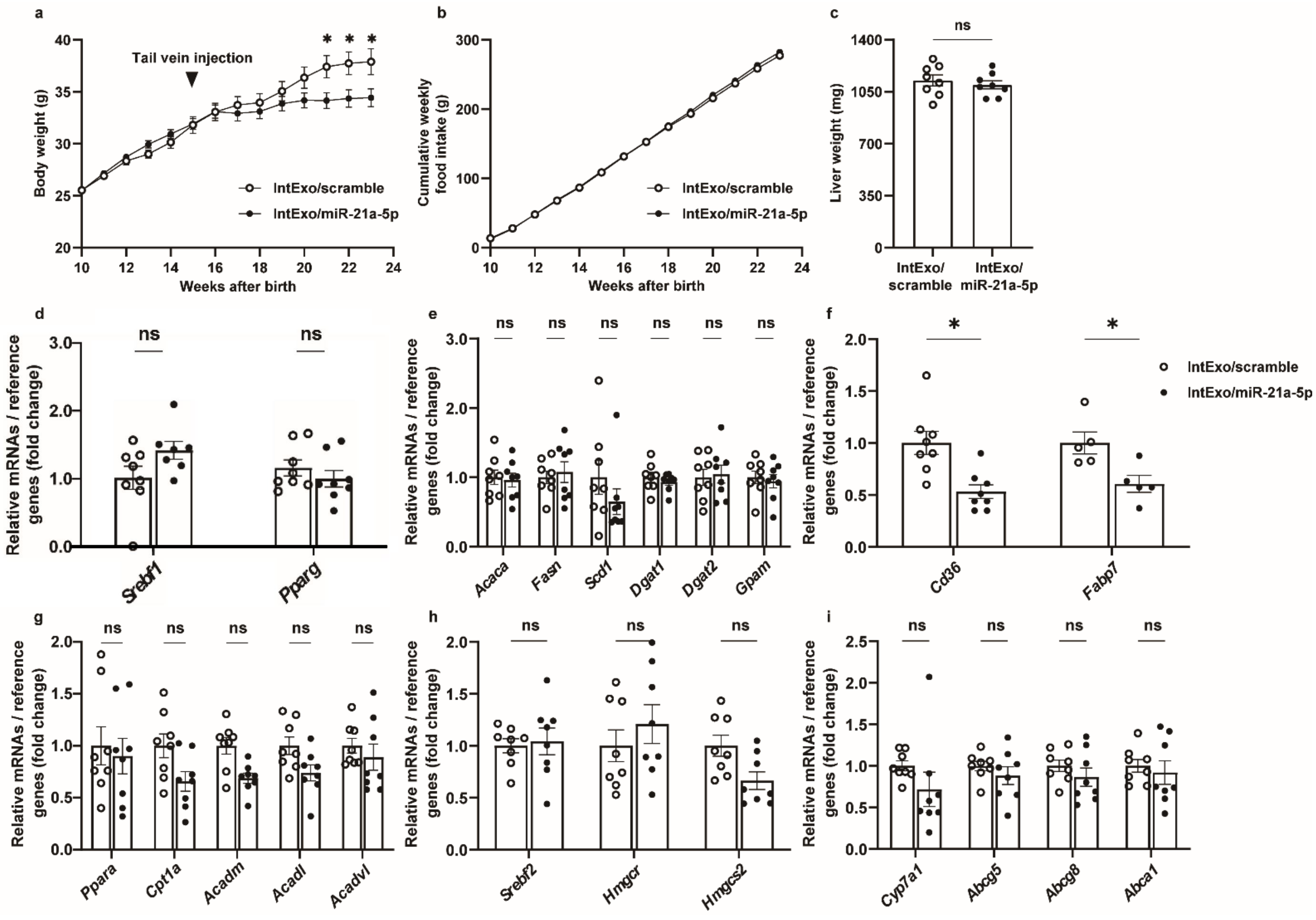

Male C57BL/6J mice were fed with HFD for 7 weeks from 8 weeks age, abd then administrated intExo/scramble or intExo/miR-21a-5p via tail vein injection at a concentration of 2 μg/g body weight twice a week for 8 consecutive weeks. The cohorts were maintained for at least 8 weeks before sacrifice. (**a**) Body weight. (**b**) Food intake. (**c**) Liver weight. mRNA expression of (**d**) lipogenesis-associated transcription factors and (**e**) genes, (**f**) lipid transport, (**g**) β-oxidation, (**h**) cholesterol synthesis, and (**i**) cholesterol transport-associated genes were determined by RT-qPCR and normalized by the geomeans of *Hprt*, *Tbp*, and *Rpl32*. All values are expressed as mean±SEM. * denotes $P < 0.05$ by two-way ANOVA or Student's *t*-test, $n = 5−8$.

Supplementary Figure S6 (Related to Figure 6) Other metabolic effects of mice treated with AAV9-miR-145a-5p-sponge.

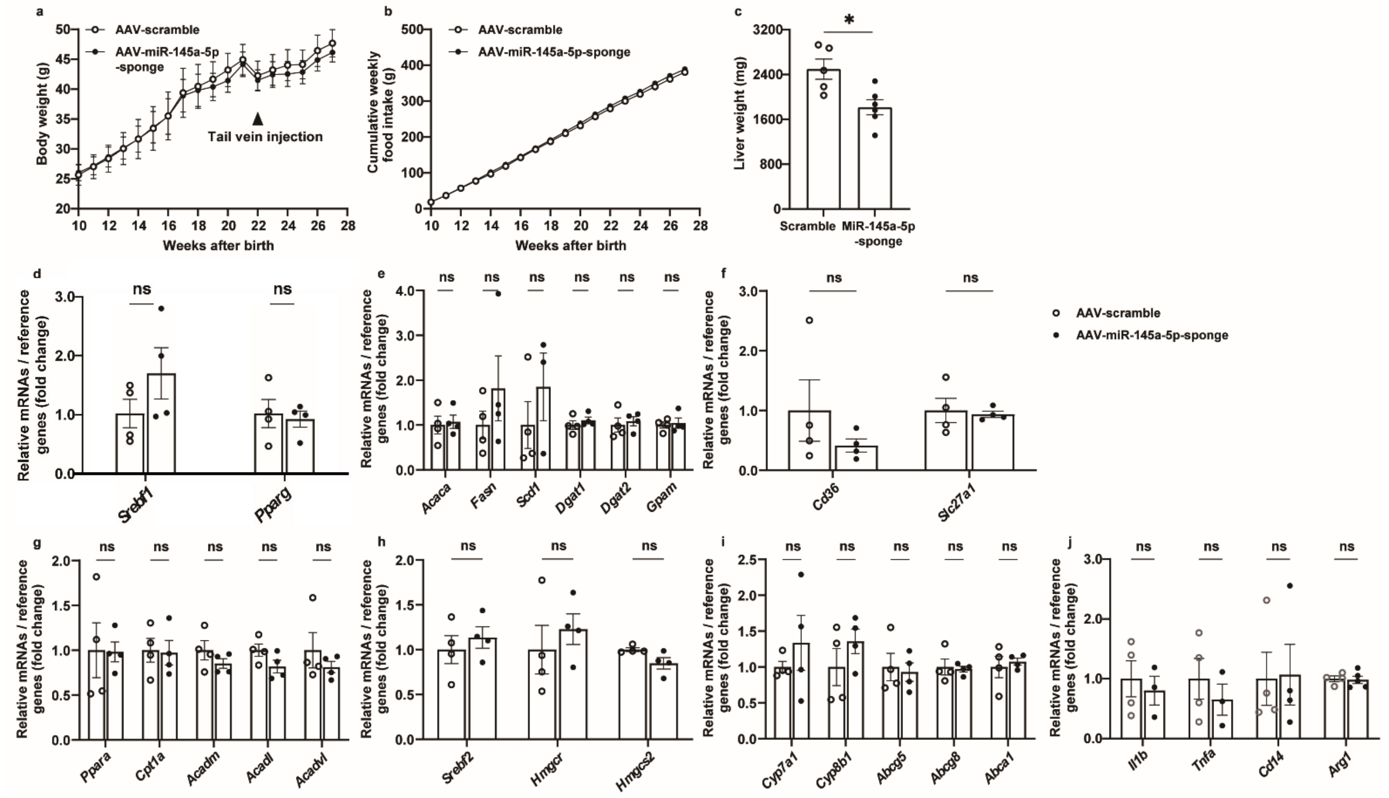

Male C57BL/6J mice were fed with HFD for 14 weeks from 8 weeks age, and then injected via tail vein with AAV9 virus expressing miR-145a-5p-sponge driven by the TBG promoter. AAV9-TBG expressing scramble sequences were used as control. The cohorts were maintained for at least 6 weeks before sacrifice to ensure adequate microRNA-sponge expression. **(a)** Body weight. **(b)** Food intake. **(c)** Liver weight. eWAT weight. sWAT weight. mRNA expression of **(d)** lipogenesis-associated transcription factors and **(e)** genes, **(f)** lipid transport, **(g)** β-oxidation, **(h)** cholesterol synthesis, **(i)** cholesterol transport, and **(j)** inflammation-associated genes were determined by RT-qPCR and normalized by the geomeans of *Hprt*, *Tbp*, and *Rpl32*. All values are expressed as mean±SEM. * denotes $P < 0.05$ by two-way ANOVA, $n = 3-5$.

Supplementary Figure S7 (Related to Figure 7) Alteration of mRNA expression by miR-21a-5p mimics in AML12 cells and miR-145a-5p mimics in Raw264.7 cells.

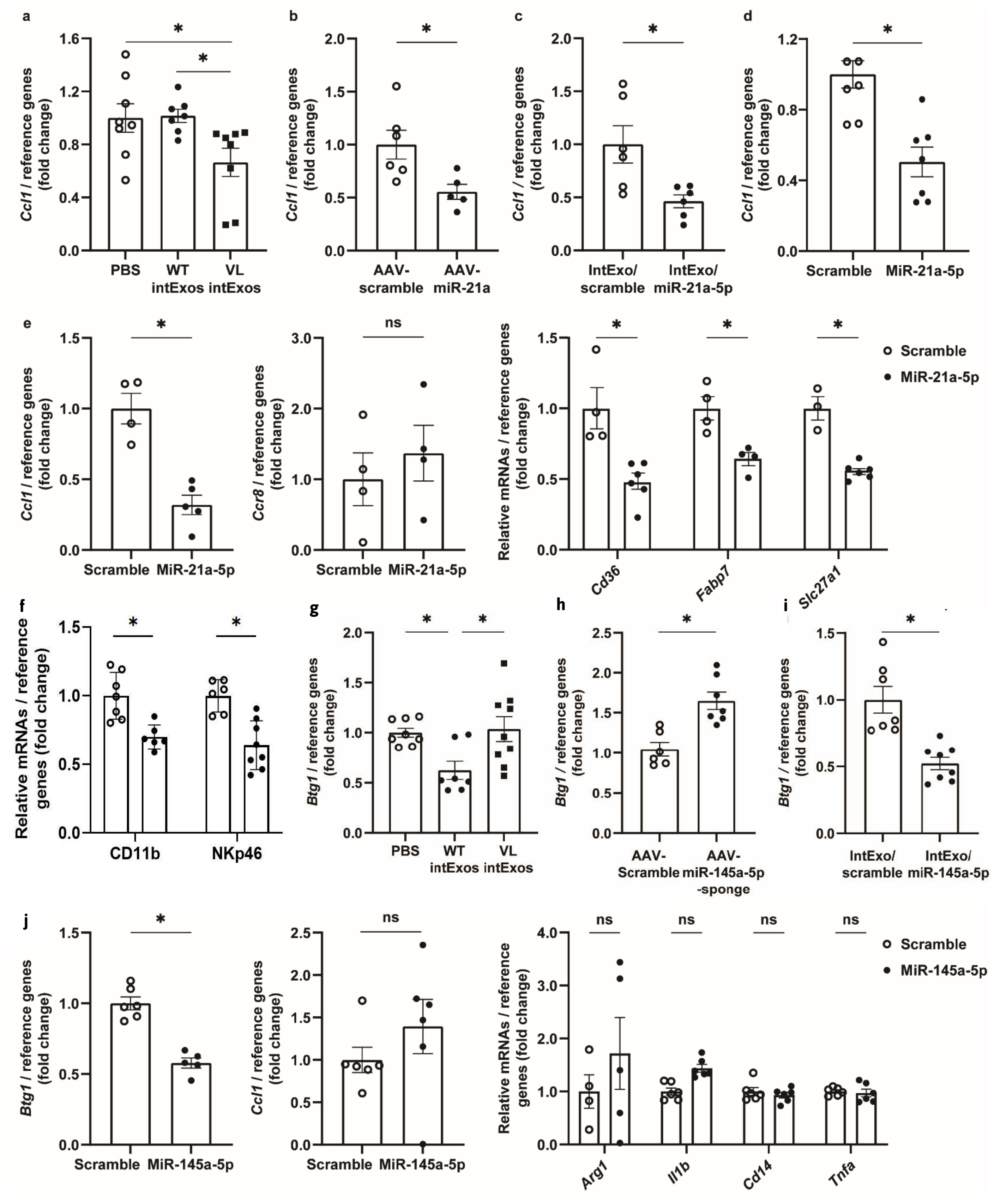

(a−d) The mRNA expression of *Ccl1* was detected in the liver of **(a)** HFD-induced obese mice treated with VL intExos, and HFD-fed mice injected with **(b)** AAV9-miR-21a or **(c)** intExo/miR-21a-5p. **(d)** Raw264.7 cells were transfected with miR-

21a-5p mimics or scramble sequences. The cellular *Ccl1* mRNA expressions were determined by real-time quantitative PCR and normalized by the geomeans of *Hprt*, *Tbp*, and *Rpl32*. All values are expressed as mean±SEM. * denotes $P<0.05$ by one-way ANOVA or Student's *t*-test, $n = 6$ for each group. **(e)** AML12 cells were treated with 300 μmol/L OA and 100 μmol/L PA before transfected with miR-21a-5p mimics or scramble sequences as negative control. The mRNA levels of *Ccl1*, *Ccr8*, and fatty acid transport-related genes were detected by RT-qPCR and normalized by the geomeans of *Hprt*, *Tbp*, and *Rpl32*. All values are expressed as mean±SEM. * denotes $P < 0.05$ by two-way ANOVA or Student's *t*-test, $n = 6$ for each group. **(f)** The mRNA expression of macrophage marker CD11b and natural killer cell marker NKp46 in the liver of HFD-fed male C57BL/6J mice injected with AAV9 virus expressing scramble sequence or miR-21a-5p driven by the TBG promoter. The mRNA expressions were determined by rRT-qPCR and normalized by β-actin expression. All values are expressed as mean±SEM. * denotes $P < 0.05$ by two-way ANOVA, $n = 6$−8 for each group. **(g–i)** The mRNA expressions of *Btg1* were detected in the liver of HFD-induced obese mice treated with **(g)** VL intExos, **(h)** AAV9-miR145a-5p sponge or **(i)** intExo-miR145a-5p mimics by RT-qPCR and normalized by the geomeans of *Hprt*, *Tbp*, and *Rpl32*. All values are expressed as mean±SEM. * denotes $P < 0.05$ by one-way ANOVA or Student's *t*-test, $n = 6$ for each group. **(j)** Raw264.7 cells were transfected with miR-145a-5p mimics or scramble sequences as negative control, and the mRNA levels of *Btg1*, *Ccl1*, and inflammation marker genes were analyzed by RT-qPCR and normalized by the geomeans of *Hprt*, *Tbp*, and *Rpl32*. All values are expressed as mean±SEM. * denotes $P < 0.05$ by two-way ANOVA or Student's *t*-test, $n = 6$ for each group.
