# Supplementary material for: Regulation of hepatic lipid metabolism by intestine epithelium-derived exosomes
Source: Life Metab. 2023 Nov 21;2(6):load044. doi: 10.1093/lifemeta/load044 (PMC11749469; doi:10.1093/lifemeta/load044)
Supplement: load044_suppl_Supplementary_Tables [file load044_suppl_Supplementary_Tables.docx]

**Supplemental Table: microRNA readcounts from small RNA sequencing data of WT and VL intExos**

| sRNA.readcount | VL_1.readcount | VL_2.readcount | VL_3.readcount | WT_1.readcount | WT_2.readcount | WT_3.readcount | VL_1.tpm | VL_2.tpm | VL_3.tpm | WT_1.tpm | WT_2.tpm | WT_3.tpm |
| --- | --- | --- | --- | --- | --- | --- | --- | --- | --- | --- | --- | --- |
| mmu-let-7a-1-3p | 127 | 89 | 133 | 119 | 112 | 186 | 14.45409 | 11.14509 | 15.45034 | 13.44153 | 12.48174 | 20.64593 |
| mmu-let-7a-2-3p | 1 | 0 | 0 | 0 | 2 | 2 | 0.113812 | 0 | 0 | 0 | 0.222888 | 0.221999 |
| mmu-let-7a-5p | 5919 | 5569 | 5735 | 8516 | 10563 | 14553 | 673.6515 | 697.382 | 666.2233 | 961.9166 | 1177.184 | 1615.377 |
| mmu-let-7b-3p | 21 | 17 | 24 | 18 | 10 | 31 | 2.390046 | 2.128837 | 2.788031 | 2.033173 | 1.114441 | 3.440988 |
| mmu-let-7b-5p | 2857 | 2479 | 2731 | 3739 | 4233 | 6072 | 325.1601 | 310.4346 | 317.2547 | 422.3352 | 471.743 | 673.9896 |
| mmu-let-7c-5p | 7994 | 7480 | 7789 | 10720 | 12604 | 15995 | 909.8108 | 936.6884 | 904.8323 | 1210.867 | 1404.642 | 1775.439 |
| mmu-let-7d-3p | 347 | 313 | 363 | 383 | 399 | 827 | 39.49266 | 39.19565 | 42.16897 | 43.2614 | 44.46621 | 91.79667 |
| mmu-let-7d-5p | 1273 | 1249 | 1366 | 1594 | 1877 | 2649 | 144.8823 | 156.4069 | 158.6854 | 180.0487 | 209.1806 | 294.038 |
| mmu-let-7e-3p | 0 | 1 | 3 | 4 | 7 | 13 | 0 | 0.125226 | 0.348504 | 0.451816 | 0.780109 | 1.442995 |
| mmu-let-7e-5p | 52 | 47 | 57 | 274 | 351 | 1206 | 5.918209 | 5.885609 | 6.621574 | 30.94941 | 39.11689 | 133.8655 |
| mmu-let-7f-1-3p | 7 | 6 | 3 | 9 | 12 | 8 | 0.796682 | 0.751354 | 0.348504 | 1.016586 | 1.33733 | 0.887997 |
| mmu-let-7f-2-3p | 8 | 3 | 7 | 6 | 10 | 16 | 0.910494 | 0.375677 | 0.813176 | 0.677724 | 1.114441 | 1.775994 |
| mmu-let-7f-5p | 42296 | 40371 | 39434 | 56692 | 66478 | 86447 | 4813.78 | 5055.488 | 4580.968 | 6403.591 | 7408.583 | 9595.583 |
| mmu-let-7g-3p | 5 | 4 | 1 | 4 | 9 | 6 | 0.569059 | 0.500903 | 0.116168 | 0.451816 | 1.002997 | 0.665998 |
| mmu-let-7g-5p | 45891 | 43467 | 45670 | 59187 | 66149 | 75014 | 5222.933 | 5443.186 | 5305.391 | 6685.411 | 7371.918 | 8326.524 |
| mmu-let-7i-3p | 26 | 30 | 31 | 42 | 50 | 62 | 2.959104 | 3.756772 | 3.601207 | 4.74407 | 5.572207 | 6.881976 |
| mmu-let-7i-5p | 39031 | 35192 | 38097 | 36178 | 40985 | 55085 | 4442.185 | 4406.944 | 4425.651 | 4086.451 | 4567.538 | 6114.413 |
| mmu-let-7j | 7 | 9 | 7 | 5 | 4 | 7 | 0.796682 | 1.127031 | 0.813176 | 0.56477 | 0.445777 | 0.776997 |
| mmu-miR-100-3p | 0 | 0 | 0 | 0 | 1 | 3 | 0 | 0 | 0 | 0 | 0.111444 | 0.332999 |
| mmu-miR-100-5p | 517 | 409 | 490 | 2895 | 3159 | 9974 | 58.84065 | 51.21732 | 56.9223 | 327.002 | 352.052 | 1107.11 |
| mmu-miR-101a-3p | 6255 | 5828 | 6315 | 11472 | 13025 | 26631 | 711.8923 | 729.8155 | 733.6007 | 1295.809 | 1451.56 | 2956.031 |
| mmu-miR-101a-5p | 2 | 2 | 0 | 3 | 1 | 3 | 0.227623 | 0.250451 | 0 | 0.338862 | 0.111444 | 0.332999 |
| mmu-miR-101b-3p | 7107 | 6511 | 7280 | 9042 | 9685 | 12250 | 808.8598 | 815.3447 | 845.7028 | 1021.33 | 1079.336 | 1359.745 |
| mmu-miR-101b-5p | 0 | 0 | 2 | 2 | 0 | 0 | 0 | 0 | 0.232336 | 0.225908 | 0 | 0 |
| mmu-miR-101c | 3 | 0 | 1 | 2 | 5 | 1 | 0.341435 | 0 | 0.116168 | 0.225908 | 0.557221 | 0.111 |
| mmu-miR-103-1-5p | 1 | 0 | 0 | 0 | 0 | 0 | 0.113812 | 0 | 0 | 0 | 0 | 0 |
| mmu-miR-103-2-5p | 2 | 0 | 0 | 1 | 0 | 1 | 0.227623 | 0 | 0 | 0.112954 | 0 | 0.111 |
| mmu-miR-103-3p | 9046 | 8539 | 8764 | 11087 | 11112 | 11183 | 1029.541 | 1069.302 | 1018.096 | 1252.321 | 1238.367 | 1241.309 |
| mmu-miR-106a-5p | 27 | 25 | 42 | 94 | 78 | 72 | 3.072916 | 3.130643 | 4.879055 | 10.61768 | 8.692642 | 7.991972 |
| mmu-miR-106b-3p | 544 | 518 | 568 | 416 | 415 | 662 | 61.91357 | 64.86692 | 65.98341 | 46.98888 | 46.24931 | 73.48174 |
| mmu-miR-106b-5p | 637 | 588 | 723 | 955 | 855 | 1183 | 72.49806 | 73.63272 | 83.98944 | 107.8711 | 95.28473 | 131.3125 |
| mmu-miR-107-3p | 396 | 399 | 439 | 562 | 527 | 600 | 45.06944 | 49.96506 | 50.99774 | 63.48017 | 58.73106 | 66.59976 |
| mmu-miR-107-5p | 0 | 0 | 0 | 0 | 1 | 0 | 0 | 0 | 0 | 0 | 0.111444 | 0 |
| mmu-miR-10a-3p | 39 | 59 | 32 | 75 | 120 | 286 | 4.438657 | 7.388317 | 3.717375 | 8.471553 | 13.3733 | 31.74589 |
| mmu-miR-10a-5p | 15575 | 13806 | 14473 | 35368 | 35969 | 99957 | 1772.617 | 1728.866 | 1681.299 | 3994.959 | 4008.534 | 11095.19 |
| mmu-miR-10b-3p | 14 | 13 | 6 | 11 | 10 | 17 | 1.593364 | 1.627934 | 0.697008 | 1.242494 | 1.114441 | 1.886993 |
| mmu-miR-10b-5p | 4352 | 3957 | 3866 | 6757 | 6779 | 17674 | 495.3086 | 495.5182 | 449.1054 | 763.2305 | 755.4798 | 1961.807 |
| mmu-miR-1187 | 0 | 0 | 0 | 1 | 0 | 0 | 0 | 0 | 0 | 0.112954 | 0 | 0 |
| mmu-miR-1188-5p | 0 | 0 | 0 | 0 | 1 | 0 | 0 | 0 | 0 | 0 | 0.111444 | 0 |
| mmu-miR-1191a | 90 | 84 | 78 | 66 | 68 | 43 | 10.24305 | 10.51896 | 9.061101 | 7.454967 | 7.578201 | 4.772983 |
| mmu-miR-1191b-5p | 2 | 2 | 2 | 1 | 1 | 7 | 0.227623 | 0.250451 | 0.232336 | 0.112954 | 0.111444 | 0.776997 |
| mmu-miR-1193-3p | 0 | 0 | 0 | 0 | 0 | 1 | 0 | 0 | 0 | 0 | 0 | 0.111 |
| mmu-miR-1195 | 1 | 0 | 0 | 0 | 0 | 1 | 0.113812 | 0 | 0 | 0 | 0 | 0.111 |
| mmu-miR-1198-3p | 5 | 2 | 5 | 1 | 2 | 3 | 0.569059 | 0.250451 | 0.58084 | 0.112954 | 0.222888 | 0.332999 |
| mmu-miR-1198-5p | 717 | 674 | 706 | 500 | 540 | 631 | 81.603 | 84.40213 | 82.01458 | 56.47702 | 60.17983 | 70.04075 |
| mmu-miR-1199-3p | 0 | 0 | 0 | 0 | 0 | 1 | 0 | 0 | 0 | 0 | 0 | 0.111 |
| mmu-miR-1199-5p | 0 | 2 | 0 | 0 | 0 | 1 | 0 | 0.250451 | 0 | 0 | 0 | 0.111 |
| mmu-miR-12178-3p | 0 | 0 | 0 | 0 | 1 | 0 | 0 | 0 | 0 | 0 | 0.111444 | 0 |
| mmu-miR-12182-3p | 15 | 13 | 9 | 18 | 10 | 5 | 1.707176 | 1.627934 | 1.045512 | 2.033173 | 1.114441 | 0.554998 |
| mmu-miR-12184-3p | 6 | 6 | 4 | 1 | 5 | 3 | 0.68287 | 0.751354 | 0.464672 | 0.112954 | 0.557221 | 0.332999 |
| mmu-miR-12184-5p | 3 | 7 | 6 | 2 | 3 | 4 | 0.341435 | 0.87658 | 0.697008 | 0.225908 | 0.334332 | 0.443998 |
| mmu-miR-12185-3p | 0 | 0 | 1 | 0 | 0 | 1 | 0 | 0 | 0.116168 | 0 | 0 | 0.111 |
| mmu-miR-12187-5p | 0 | 0 | 0 | 0 | 0 | 1 | 0 | 0 | 0 | 0 | 0 | 0.111 |
| mmu-miR-12191-3p | 0 | 0 | 0 | 1 | 2 | 2 | 0 | 0 | 0 | 0.112954 | 0.222888 | 0.221999 |
| mmu-miR-12193-5p | 0 | 1 | 0 | 0 | 2 | 0 | 0 | 0.125226 | 0 | 0 | 0.222888 | 0 |
| mmu-miR-12194-3p | 1 | 0 | 4 | 1 | 1 | 3 | 0.113812 | 0 | 0.464672 | 0.112954 | 0.111444 | 0.332999 |
| mmu-miR-12199-3p | 1 | 0 | 0 | 1 | 0 | 0 | 0.113812 | 0 | 0 | 0.112954 | 0 | 0 |
| mmu-miR-12201-3p | 0 | 1 | 0 | 0 | 0 | 2 | 0 | 0.125226 | 0 | 0 | 0 | 0.221999 |
| mmu-miR-12201-5p | 4 | 0 | 0 | 0 | 0 | 0 | 0.455247 | 0 | 0 | 0 | 0 | 0 |
| mmu-miR-12202-3p | 0 | 2 | 1 | 0 | 0 | 0 | 0 | 0.250451 | 0.116168 | 0 | 0 | 0 |
| mmu-miR-12206-5p | 0 | 0 | 1 | 1 | 2 | 2 | 0 | 0 | 0.116168 | 0.112954 | 0.222888 | 0.221999 |
| mmu-miR-122-3p | 1 | 2 | 0 | 0 | 2 | 0 | 0.113812 | 0.250451 | 0 | 0 | 0.222888 | 0 |
| mmu-miR-1224-5p | 2 | 4 | 0 | 0 | 4 | 2 | 0.227623 | 0.500903 | 0 | 0 | 0.445777 | 0.221999 |
| mmu-miR-122-5p | 499 | 421 | 547 | 402 | 361 | 344 | 56.79204 | 52.72003 | 63.54388 | 45.40753 | 40.23133 | 38.18386 |
| mmu-miR-122b-3p | 499 | 421 | 547 | 402 | 361 | 344 | 56.79204 | 52.72003 | 63.54388 | 45.40753 | 40.23133 | 38.18386 |
| mmu-miR-122b-5p | 0 | 1 | 0 | 0 | 0 | 0 | 0 | 0.125226 | 0 | 0 | 0 | 0 |
| mmu-miR-1231-5p | 0 | 0 | 1 | 0 | 0 | 1 | 0 | 0 | 0.116168 | 0 | 0 | 0.111 |
| mmu-miR-124-3p | 5 | 4 | 2 | 4 | 5 | 24 | 0.569059 | 0.500903 | 0.232336 | 0.451816 | 0.557221 | 2.663991 |
| mmu-miR-124-5p | 0 | 2 | 0 | 0 | 1 | 0 | 0 | 0.250451 | 0 | 0 | 0.111444 | 0 |
| mmu-miR-1247-3p | 0 | 0 | 0 | 0 | 0 | 1 | 0 | 0 | 0 | 0 | 0 | 0.111 |
| mmu-miR-1249-5p | 0 | 0 | 0 | 0 | 1 | 1 | 0 | 0 | 0 | 0 | 0.111444 | 0.111 |
| mmu-miR-124b-3p | 0 | 2 | 0 | 0 | 1 | 0 | 0 | 0.250451 | 0 | 0 | 0.111444 | 0 |
| mmu-miR-125a-3p | 0 | 1 | 0 | 1 | 7 | 23 | 0 | 0.125226 | 0 | 0.112954 | 0.780109 | 2.552991 |
| mmu-miR-125a-5p | 82 | 48 | 51 | 312 | 425 | 1733 | 9.33256 | 6.010835 | 5.924566 | 35.24166 | 47.36376 | 192.3623 |
| mmu-miR-125b-1-3p | 2 | 3 | 2 | 8 | 13 | 46 | 0.227623 | 0.375677 | 0.232336 | 0.903632 | 1.448774 | 5.105982 |
| mmu-miR-125b-2-3p | 2 | 1 | 3 | 8 | 14 | 53 | 0.227623 | 0.125226 | 0.348504 | 0.903632 | 1.560218 | 5.882979 |
| mmu-miR-125b-5p | 115 | 98 | 114 | 621 | 822 | 2595 | 13.08835 | 12.27212 | 13.24315 | 70.14446 | 91.60708 | 288.044 |
| mmu-miR-1264-3p | 0 | 0 | 0 | 0 | 0 | 4 | 0 | 0 | 0 | 0 | 0 | 0.443998 |
| mmu-miR-1264-5p | 0 | 0 | 0 | 0 | 1 | 1 | 0 | 0 | 0 | 0 | 0.111444 | 0.111 |
| mmu-miR-126a-3p | 453 | 258 | 333 | 9126 | 8721 | 37005 | 51.55671 | 32.30824 | 38.68393 | 1030.819 | 971.9043 | 4107.54 |
| mmu-miR-126a-5p | 42 | 19 | 31 | 734 | 760 | 4054 | 4.780092 | 2.379289 | 3.601207 | 82.90827 | 84.69754 | 449.9924 |
| mmu-miR-126b-3p | 42 | 19 | 31 | 734 | 760 | 4054 | 4.780092 | 2.379289 | 3.601207 | 82.90827 | 84.69754 | 449.9924 |
| mmu-miR-126b-5p | 2 | 0 | 2 | 31 | 35 | 161 | 0.227623 | 0 | 0.232336 | 3.501575 | 3.900545 | 17.87094 |
| mmu-miR-127-3p | 39 | 48 | 59 | 267 | 303 | 1282 | 4.438657 | 6.010835 | 6.85391 | 30.15873 | 33.76757 | 142.3015 |
| mmu-miR-127-5p | 2 | 3 | 6 | 29 | 33 | 135 | 0.227623 | 0.375677 | 0.697008 | 3.275667 | 3.677656 | 14.98495 |
| mmu-miR-128-1-5p | 13 | 3 | 5 | 5 | 2 | 3 | 1.479552 | 0.375677 | 0.58084 | 0.56477 | 0.222888 | 0.332999 |
| mmu-miR-128-3p | 877 | 720 | 819 | 679 | 985 | 899 | 99.81287 | 90.16252 | 95.14157 | 76.6958 | 109.7725 | 99.78865 |
| mmu-miR-1291 | 35 | 35 | 36 | 75 | 45 | 62 | 3.98341 | 4.3829 | 4.182047 | 8.471553 | 5.014986 | 6.881976 |
| mmu-miR-129-1-3p | 2 | 0 | 0 | 2 | 4 | 10 | 0.227623 | 0 | 0 | 0.225908 | 0.445777 | 1.109996 |
| mmu-miR-129-2-3p | 1 | 0 | 0 | 9 | 12 | 34 | 0.113812 | 0 | 0 | 1.016586 | 1.33733 | 3.773987 |
| mmu-miR-129-5p | 9 | 24 | 8 | 87 | 107 | 355 | 1.024305 | 3.005417 | 0.929344 | 9.827002 | 11.92452 | 39.40486 |
| mmu-miR-1298-3p | 0 | 0 | 0 | 0 | 1 | 1 | 0 | 0 | 0 | 0 | 0.111444 | 0.111 |
| mmu-miR-1298-5p | 0 | 3 | 0 | 9 | 3 | 19 | 0 | 0.375677 | 0 | 1.016586 | 0.334332 | 2.108993 |
| mmu-miR-129b-3p | 9 | 24 | 8 | 87 | 107 | 353 | 1.024305 | 3.005417 | 0.929344 | 9.827002 | 11.92452 | 39.18286 |
| mmu-miR-129b-5p | 0 | 0 | 0 | 1 | 7 | 4 | 0 | 0 | 0 | 0.112954 | 0.780109 | 0.443998 |
| mmu-miR-1306-3p | 1 | 1 | 2 | 0 | 1 | 1 | 0.113812 | 0.125226 | 0.232336 | 0 | 0.111444 | 0.111 |
| mmu-miR-1306-5p | 10 | 8 | 10 | 12 | 5 | 11 | 1.138117 | 1.001806 | 1.16168 | 1.355449 | 0.557221 | 1.220996 |
| mmu-miR-130a-3p | 3 | 7 | 2 | 18 | 23 | 73 | 0.341435 | 0.87658 | 0.232336 | 2.033173 | 2.563215 | 8.102971 |
| mmu-miR-130a-5p | 0 | 0 | 1 | 0 | 0 | 4 | 0 | 0 | 0.116168 | 0 | 0 | 0.443998 |
| mmu-miR-130b-3p | 23 | 21 | 23 | 18 | 34 | 29 | 2.617669 | 2.62974 | 2.671863 | 2.033173 | 3.7891 | 3.218989 |
| mmu-miR-130b-5p | 235 | 217 | 241 | 165 | 200 | 160 | 26.74575 | 27.17398 | 27.99648 | 18.63742 | 22.28883 | 17.75994 |
| mmu-miR-132-3p | 94 | 86 | 84 | 100 | 106 | 443 | 10.6983 | 10.76941 | 9.758109 | 11.2954 | 11.81308 | 49.17283 |
| mmu-miR-132-5p | 83 | 70 | 87 | 69 | 76 | 167 | 9.446372 | 8.7658 | 10.10661 | 7.793829 | 8.469754 | 18.53693 |
| mmu-miR-133a-3p | 8 | 12 | 2 | 134 | 128 | 805 | 0.910494 | 1.502709 | 0.232336 | 15.13584 | 14.26485 | 89.35468 |
| mmu-miR-133a-5p | 0 | 3 | 0 | 5 | 2 | 12 | 0 | 0.375677 | 0 | 0.56477 | 0.222888 | 1.331995 |
| mmu-miR-133b-3p | 2 | 0 | 1 | 5 | 9 | 57 | 0.227623 | 0 | 0.116168 | 0.56477 | 1.002997 | 6.326978 |
| mmu-miR-134-3p | 0 | 0 | 0 | 0 | 0 | 3 | 0 | 0 | 0 | 0 | 0 | 0.332999 |
| mmu-miR-134-5p | 0 | 2 | 3 | 16 | 21 | 67 | 0 | 0.250451 | 0.348504 | 1.807265 | 2.340327 | 7.436974 |
| mmu-miR-135a-1-3p | 0 | 0 | 0 | 0 | 3 | 1 | 0 | 0 | 0 | 0 | 0.334332 | 0.111 |
| mmu-miR-135a-2-3p | 0 | 0 | 0 | 2 | 0 | 3 | 0 | 0 | 0 | 0.225908 | 0 | 0.332999 |
| mmu-miR-135a-5p | 1 | 1 | 1 | 12 | 21 | 62 | 0.113812 | 0.125226 | 0.116168 | 1.355449 | 2.340327 | 6.881976 |
| mmu-miR-135b-5p | 3 | 2 | 1 | 6 | 0 | 9 | 0.341435 | 0.250451 | 0.116168 | 0.677724 | 0 | 0.998996 |
| mmu-miR-136-3p | 14 | 8 | 6 | 109 | 135 | 427 | 1.593364 | 1.001806 | 0.697008 | 12.31199 | 15.04496 | 47.39683 |
| mmu-miR-136-5p | 0 | 0 | 4 | 7 | 19 | 31 | 0 | 0 | 0.464672 | 0.790678 | 2.117438 | 3.440988 |
| mmu-miR-137-3p | 0 | 1 | 2 | 15 | 7 | 52 | 0 | 0.125226 | 0.232336 | 1.694311 | 0.780109 | 5.77198 |
| mmu-miR-138-5p | 3 | 4 | 13 | 22 | 34 | 139 | 0.341435 | 0.500903 | 1.510184 | 2.484989 | 3.7891 | 15.42895 |
| mmu-miR-139-3p | 3 | 5 | 5 | 8 | 9 | 15 | 0.341435 | 0.626129 | 0.58084 | 0.903632 | 1.002997 | 1.664994 |
| mmu-miR-139-5p | 285 | 246 | 274 | 665 | 642 | 1444 | 32.43634 | 30.80553 | 31.83002 | 75.11444 | 71.54713 | 160.2834 |
| mmu-miR-140-3p | 5483 | 5291 | 4916 | 6967 | 7785 | 10756 | 624.0296 | 662.5693 | 571.0817 | 786.9508 | 867.5926 | 1193.912 |
| mmu-miR-140-5p | 786 | 752 | 718 | 866 | 905 | 1084 | 89.45601 | 94.16974 | 83.4086 | 97.8182 | 100.8569 | 120.3236 |
| mmu-miR-141-3p | 1221 | 1027 | 1292 | 2178 | 2086 | 1463 | 138.9641 | 128.6068 | 150.089 | 246.0139 | 232.4725 | 162.3924 |
| mmu-miR-141-5p | 158 | 154 | 142 | 216 | 160 | 151 | 17.98225 | 19.28476 | 16.49585 | 24.39807 | 17.83106 | 16.76094 |
| mmu-miR-142a-3p | 1653 | 1482 | 1561 | 1608 | 1530 | 5034 | 188.1308 | 185.5845 | 181.3382 | 181.6301 | 170.5095 | 558.772 |
| mmu-miR-142a-5p | 3094 | 2845 | 3340 | 2871 | 3185 | 8118 | 352.1334 | 356.2672 | 388.001 | 324.2911 | 354.9496 | 901.0948 |
| mmu-miR-142b | 1653 | 1482 | 1561 | 1608 | 1530 | 5034 | 188.1308 | 185.5845 | 181.3382 | 181.6301 | 170.5095 | 558.772 |
| mmu-miR-143-3p | 57547 | 56883 | 67601 | 570471 | 611955 | 1941313 | 6549.523 | 7123.215 | 7853.071 | 64437.01 | 68198.79 | 215485 |
| mmu-miR-143-5p | 30 | 13 | 22 | 380 | 378 | 1836 | 3.414351 | 1.627934 | 2.555695 | 42.92254 | 42.12588 | 203.7953 |
| mmu-miR-144-3p | 11 | 7 | 14 | 15 | 17 | 33 | 1.251929 | 0.87658 | 1.626352 | 1.694311 | 1.89455 | 3.662987 |
| mmu-miR-144-5p | 7 | 6 | 2 | 5 | 12 | 18 | 0.796682 | 0.751354 | 0.232336 | 0.56477 | 1.33733 | 1.997993 |
| mmu-miR-145a-3p | 95 | 95 | 125 | 732 | 904 | 2882 | 10.81211 | 11.89644 | 14.521 | 82.68236 | 100.7455 | 319.9009 |
| mmu-miR-145a-5p | 382 | 335 | 384 | 6690 | 6463 | 22314 | 43.47607 | 41.95062 | 44.6085 | 755.6626 | 720.2634 | 2476.845 |
| mmu-miR-145b | 0 | 0 | 0 | 0 | 0 | 1 | 0 | 0 | 0 | 0 | 0 | 0.111 |
| mmu-miR-146a-3p | 0 | 0 | 3 | 0 | 0 | 2 | 0 | 0 | 0.348504 | 0 | 0 | 0.221999 |
| mmu-miR-146a-5p | 3084 | 3017 | 2936 | 2531 | 2245 | 4467 | 350.9953 | 377.806 | 341.0692 | 285.8867 | 250.1921 | 495.8352 |
| mmu-miR-146b-3p | 1 | 0 | 1 | 3 | 2 | 1 | 0.113812 | 0 | 0.116168 | 0.338862 | 0.222888 | 0.111 |
| mmu-miR-146b-5p | 1371 | 1255 | 1262 | 2260 | 2163 | 2452 | 156.0359 | 157.1583 | 146.604 | 255.2761 | 241.0537 | 272.171 |
| mmu-miR-147-3p | 619 | 612 | 675 | 439 | 484 | 392 | 70.44945 | 76.63814 | 78.41338 | 49.58683 | 53.93896 | 43.51185 |
| mmu-miR-147-5p | 38 | 34 | 43 | 29 | 21 | 14 | 4.324845 | 4.257674 | 4.995223 | 3.275667 | 2.340327 | 1.553994 |
| mmu-miR-148a-3p | 71667 | 66700 | 70257 | 181469 | 184403 | 175142 | 8156.544 | 8352.555 | 8161.613 | 20497.66 | 20550.63 | 19440.69 |
| mmu-miR-148a-5p | 330 | 365 | 378 | 689 | 734 | 728 | 37.55786 | 45.70739 | 43.91149 | 77.82534 | 81.79999 | 80.80771 |
| mmu-miR-148b-3p | 2301 | 2133 | 2256 | 1737 | 2235 | 2264 | 261.8807 | 267.1065 | 262.0749 | 196.2012 | 249.0776 | 251.3031 |
| mmu-miR-148b-5p | 205 | 186 | 173 | 186 | 253 | 207 | 23.3314 | 23.29198 | 20.09706 | 21.00945 | 28.19537 | 22.97692 |
| mmu-miR-149-5p | 26 | 22 | 13 | 39 | 61 | 128 | 2.959104 | 2.754966 | 1.510184 | 4.405208 | 6.798092 | 14.20795 |
| mmu-miR-150-3p | 10 | 9 | 9 | 6 | 4 | 21 | 1.138117 | 1.127031 | 1.045512 | 0.677724 | 0.445777 | 2.330992 |
| mmu-miR-150-5p | 560 | 434 | 495 | 473 | 499 | 2796 | 63.73456 | 54.34796 | 57.50314 | 53.42726 | 55.61062 | 310.3549 |
| mmu-miR-151-3p | 4185 | 3766 | 4326 | 2977 | 3388 | 3480 | 476.302 | 471.6001 | 502.5426 | 336.2642 | 377.5727 | 386.2786 |
| mmu-miR-151-5p | 198 | 144 | 164 | 243 | 262 | 433 | 22.53472 | 18.0325 | 19.05155 | 27.44783 | 29.19836 | 48.06283 |
| mmu-miR-152-3p | 189 | 151 | 180 | 1172 | 1261 | 2744 | 21.51041 | 18.90908 | 20.91023 | 132.3821 | 140.531 | 304.5829 |
| mmu-miR-152-5p | 6 | 7 | 15 | 65 | 63 | 98 | 0.68287 | 0.87658 | 1.74252 | 7.342013 | 7.02098 | 10.87796 |
| mmu-miR-153-3p | 3 | 4 | 1 | 15 | 10 | 9 | 0.341435 | 0.500903 | 0.116168 | 1.694311 | 1.114441 | 0.998996 |
| mmu-miR-153-5p | 0 | 0 | 1 | 0 | 1 | 1 | 0 | 0 | 0.116168 | 0 | 0.111444 | 0.111 |
| mmu-miR-154-3p | 0 | 0 | 0 | 0 | 0 | 7 | 0 | 0 | 0 | 0 | 0 | 0.776997 |
| mmu-miR-154-5p | 1 | 3 | 1 | 2 | 0 | 22 | 0.113812 | 0.375677 | 0.116168 | 0.225908 | 0 | 2.441991 |
| mmu-miR-155-3p | 4 | 1 | 4 | 4 | 4 | 3 | 0.455247 | 0.125226 | 0.464672 | 0.451816 | 0.445777 | 0.332999 |
| mmu-miR-155-5p | 111 | 111 | 113 | 125 | 129 | 489 | 12.6331 | 13.90005 | 13.12698 | 14.11926 | 14.37629 | 54.27881 |
| mmu-miR-15a-3p | 5 | 3 | 7 | 5 | 4 | 2 | 0.569059 | 0.375677 | 0.813176 | 0.56477 | 0.445777 | 0.221999 |
| mmu-miR-15a-5p | 120 | 100 | 108 | 141 | 119 | 191 | 13.65741 | 12.52257 | 12.54614 | 15.92652 | 13.26185 | 21.20092 |
| mmu-miR-15b-3p | 186 | 99 | 192 | 132 | 149 | 214 | 21.16898 | 12.39735 | 22.30425 | 14.90993 | 16.60518 | 23.75392 |
| mmu-miR-15b-5p | 446 | 372 | 420 | 565 | 558 | 659 | 50.76002 | 46.58397 | 48.79055 | 63.81903 | 62.18583 | 73.14874 |
| mmu-miR-16-1-3p | 954 | 904 | 998 | 1107 | 1075 | 1187 | 108.5764 | 113.2041 | 115.9356 | 125.0401 | 119.8024 | 131.7565 |
| mmu-miR-16-2-3p | 8 | 13 | 16 | 28 | 20 | 29 | 0.910494 | 1.627934 | 1.858687 | 3.162713 | 2.228883 | 3.218989 |
| mmu-miR-16-5p | 2727 | 2562 | 2689 | 5222 | 4616 | 8849 | 310.3645 | 320.8283 | 312.3757 | 589.846 | 514.4261 | 982.2355 |
| mmu-miR-17-3p | 88 | 103 | 116 | 95 | 96 | 92 | 10.01543 | 12.89825 | 13.47548 | 10.73063 | 10.69864 | 10.21196 |
| mmu-miR-17-5p | 1604 | 1475 | 1636 | 1761 | 1613 | 1818 | 182.554 | 184.7079 | 190.0508 | 198.9121 | 179.7594 | 201.7973 |
| mmu-miR-181a-1-3p | 36 | 27 | 20 | 123 | 124 | 180 | 4.097222 | 3.381094 | 2.323359 | 13.89335 | 13.81907 | 19.97993 |
| mmu-miR-181a-2-3p | 0 | 3 | 4 | 24 | 16 | 28 | 0 | 0.375677 | 0.464672 | 2.710897 | 1.783106 | 3.107989 |
| mmu-miR-181a-5p | 424 | 413 | 430 | 2530 | 2515 | 5462 | 48.25617 | 51.71822 | 49.95223 | 285.7737 | 280.282 | 606.2798 |
| mmu-miR-181b-1-3p | 0 | 1 | 0 | 6 | 2 | 7 | 0 | 0.125226 | 0 | 0.677724 | 0.222888 | 0.776997 |
| mmu-miR-181b-2-3p | 0 | 0 | 0 | 0 | 0 | 1 | 0 | 0 | 0 | 0 | 0 | 0.111 |
| mmu-miR-181b-5p | 71 | 59 | 64 | 402 | 466 | 999 | 8.080632 | 7.388317 | 7.43475 | 45.40753 | 51.93297 | 110.8886 |
| mmu-miR-181c-3p | 50 | 31 | 57 | 127 | 142 | 154 | 5.690586 | 3.881997 | 6.621574 | 14.34516 | 15.82507 | 17.09394 |
| mmu-miR-181c-5p | 27 | 23 | 16 | 87 | 83 | 119 | 3.072916 | 2.880192 | 1.858687 | 9.827002 | 9.249863 | 13.20895 |
| mmu-miR-181d-5p | 102 | 91 | 101 | 375 | 371 | 485 | 11.60879 | 11.39554 | 11.73296 | 42.35777 | 41.34577 | 53.83481 |
| mmu-miR-182-3p | 8 | 14 | 18 | 9 | 12 | 1 | 0.910494 | 1.75316 | 2.091023 | 1.016586 | 1.33733 | 0.111 |
| mmu-miR-182-5p | 13698 | 12733 | 12166 | 17255 | 16693 | 13165 | 1558.993 | 1594.499 | 1413.299 | 1949.022 | 1860.337 | 1461.31 |
| mmu-miR-183-3p | 7 | 14 | 9 | 26 | 25 | 5 | 0.796682 | 1.75316 | 1.045512 | 2.936805 | 2.786103 | 0.554998 |
| mmu-miR-183-5p | 8594 | 7591 | 7963 | 8186 | 9167 | 6284 | 978.0978 | 950.5884 | 925.0455 | 924.6418 | 1021.608 | 697.5215 |
| mmu-miR-1839-3p | 12 | 15 | 5 | 23 | 23 | 46 | 1.365741 | 1.878386 | 0.58084 | 2.597943 | 2.563215 | 5.105982 |
| mmu-miR-1839-5p | 737 | 657 | 729 | 1061 | 1267 | 1998 | 83.87923 | 82.2733 | 84.68645 | 119.8442 | 141.1997 | 221.7772 |
| mmu-miR-1843a-3p | 6 | 3 | 5 | 9 | 11 | 32 | 0.68287 | 0.375677 | 0.58084 | 1.016586 | 1.225885 | 3.551987 |
| mmu-miR-1843a-5p | 288 | 258 | 287 | 402 | 490 | 801 | 32.77777 | 32.30824 | 33.34021 | 45.40753 | 54.60762 | 88.91068 |
| mmu-miR-1843b-3p | 36 | 24 | 25 | 40 | 30 | 50 | 4.097222 | 3.005417 | 2.904199 | 4.518162 | 3.343324 | 5.54998 |
| mmu-miR-1843b-5p | 146 | 109 | 134 | 181 | 227 | 480 | 16.61651 | 13.6496 | 15.56651 | 20.44468 | 25.29782 | 53.27981 |
| mmu-miR-184-3p | 15 | 8 | 16 | 21 | 19 | 64 | 1.707176 | 1.001806 | 1.858687 | 2.372035 | 2.117438 | 7.103975 |
| mmu-miR-185-3p | 29 | 19 | 14 | 5 | 9 | 18 | 3.30054 | 2.379289 | 1.626352 | 0.56477 | 1.002997 | 1.997993 |
| mmu-miR-185-5p | 2171 | 2072 | 2075 | 2602 | 2607 | 1530 | 247.0852 | 259.4677 | 241.0485 | 293.9064 | 290.5348 | 169.8294 |
| mmu-miR-186-3p | 3 | 1 | 3 | 1 | 5 | 9 | 0.341435 | 0.125226 | 0.348504 | 0.112954 | 0.557221 | 0.998996 |
| mmu-miR-186-5p | 819 | 732 | 766 | 770 | 882 | 1135 | 93.21179 | 91.66523 | 88.98466 | 86.97461 | 98.29372 | 125.9846 |
| mmu-miR-187-3p | 5 | 5 | 2 | 6 | 6 | 14 | 0.569059 | 0.626129 | 0.232336 | 0.677724 | 0.668665 | 1.553994 |
| mmu-miR-188-5p | 9 | 8 | 16 | 2 | 4 | 9 | 1.024305 | 1.001806 | 1.858687 | 0.225908 | 0.445777 | 0.998996 |
| mmu-miR-18a-3p | 18 | 12 | 15 | 10 | 18 | 24 | 2.048611 | 1.502709 | 1.74252 | 1.12954 | 2.005994 | 2.663991 |
| mmu-miR-18a-5p | 484 | 389 | 458 | 450 | 408 | 513 | 55.08487 | 48.7128 | 53.20493 | 50.82932 | 45.46921 | 56.9428 |
| mmu-miR-18b-3p | 0 | 2 | 0 | 0 | 0 | 0 | 0 | 0.250451 | 0 | 0 | 0 | 0 |
| mmu-miR-18b-5p | 86 | 67 | 70 | 99 | 116 | 91 | 9.787807 | 8.390123 | 8.131758 | 11.18245 | 12.92752 | 10.10096 |
| mmu-miR-190a-3p | 0 | 2 | 1 | 2 | 2 | 3 | 0 | 0.250451 | 0.116168 | 0.225908 | 0.222888 | 0.332999 |
| mmu-miR-190a-5p | 24 | 22 | 22 | 37 | 25 | 42 | 2.731481 | 2.754966 | 2.555695 | 4.1793 | 2.786103 | 4.661983 |
| mmu-miR-190b-5p | 5 | 6 | 8 | 5 | 3 | 8 | 0.569059 | 0.751354 | 0.929344 | 0.56477 | 0.334332 | 0.887997 |
| mmu-miR-191-3p | 70 | 55 | 39 | 49 | 49 | 44 | 7.96682 | 6.887415 | 4.530551 | 5.534748 | 5.460762 | 4.883983 |
| mmu-miR-191-5p | 23860 | 21873 | 22962 | 20506 | 23981 | 22013 | 2715.547 | 2739.062 | 2667.449 | 2316.236 | 2672.542 | 2443.434 |
| mmu-miR-192-3p | 190 | 146 | 140 | 214 | 175 | 119 | 21.62423 | 18.28296 | 16.26352 | 24.17217 | 19.50272 | 13.20895 |
| mmu-miR-192-5p | 957714 | 861453 | 988220 | 1065266 | 1163423 | 820505 | 108999.1 | 107876.1 | 114799.5 | 120326.1 | 129656.7 | 91075.73 |
| mmu-miR-1927 | 2 | 7 | 3 | 6 | 4 | 1 | 0.227623 | 0.87658 | 0.348504 | 0.677724 | 0.445777 | 0.111 |
| mmu-miR-1929-5p | 1 | 0 | 3 | 0 | 1 | 3 | 0.113812 | 0 | 0.348504 | 0 | 0.111444 | 0.332999 |
| mmu-miR-1930-5p | 1 | 0 | 4 | 3 | 0 | 1 | 0.113812 | 0 | 0.464672 | 0.338862 | 0 | 0.111 |
| mmu-miR-1932 | 0 | 0 | 2 | 0 | 0 | 2 | 0 | 0 | 0.232336 | 0 | 0 | 0.221999 |
| mmu-miR-1933-3p | 8 | 4 | 14 | 1 | 3 | 0 | 0.910494 | 0.500903 | 1.626352 | 0.112954 | 0.334332 | 0 |
| mmu-miR-1933-5p | 0 | 1 | 0 | 0 | 0 | 0 | 0 | 0.125226 | 0 | 0 | 0 | 0 |
| mmu-miR-1934-3p | 0 | 1 | 0 | 1 | 0 | 0 | 0 | 0.125226 | 0 | 0.112954 | 0 | 0 |
| mmu-miR-1934-5p | 1 | 2 | 1 | 1 | 1 | 4 | 0.113812 | 0.250451 | 0.116168 | 0.112954 | 0.111444 | 0.443998 |
| mmu-miR-1936 | 26 | 24 | 32 | 14 | 17 | 4 | 2.959104 | 3.005417 | 3.717375 | 1.581357 | 1.89455 | 0.443998 |
| mmu-miR-1938 | 1 | 0 | 0 | 0 | 0 | 0 | 0.113812 | 0 | 0 | 0 | 0 | 0 |
| mmu-miR-193a-3p | 0 | 3 | 0 | 3 | 5 | 13 | 0 | 0.375677 | 0 | 0.338862 | 0.557221 | 1.442995 |
| mmu-miR-193a-5p | 0 | 0 | 1 | 3 | 4 | 17 | 0 | 0 | 0.116168 | 0.338862 | 0.445777 | 1.886993 |
| mmu-miR-193b-3p | 3 | 3 | 2 | 3 | 10 | 21 | 0.341435 | 0.375677 | 0.232336 | 0.338862 | 1.114441 | 2.330992 |
| mmu-miR-193b-5p | 0 | 0 | 1 | 0 | 1 | 1 | 0 | 0 | 0.116168 | 0 | 0.111444 | 0.111 |
| mmu-miR-194-1-3p | 14394 | 12945 | 15076 | 14446 | 17155 | 14747 | 1638.206 | 1621.047 | 1751.348 | 1631.734 | 1911.824 | 1636.911 |
| mmu-miR-1941-3p | 0 | 0 | 0 | 0 | 0 | 1 | 0 | 0 | 0 | 0 | 0 | 0.111 |
| mmu-miR-194-2-3p | 1611 | 1404 | 1649 | 1283 | 1566 | 1276 | 183.3507 | 175.8169 | 191.561 | 144.92 | 174.5215 | 141.6355 |
| mmu-miR-1943-3p | 1 | 1 | 0 | 1 | 2 | 0 | 0.113812 | 0.125226 | 0 | 0.112954 | 0.222888 | 0 |
| mmu-miR-1943-5p | 9 | 8 | 15 | 10 | 10 | 15 | 1.024305 | 1.001806 | 1.74252 | 1.12954 | 1.114441 | 1.664994 |
| mmu-miR-1945 | 3 | 5 | 4 | 1 | 0 | 1 | 0.341435 | 0.626129 | 0.464672 | 0.112954 | 0 | 0.111 |
| mmu-miR-194-5p | 344613 | 332772 | 354856 | 489332 | 473752 | 378651 | 39221 | 41671.61 | 41222.9 | 55272.03 | 52796.88 | 42030.11 |
| mmu-miR-1946a | 0 | 1 | 0 | 0 | 0 | 1 | 0 | 0.125226 | 0 | 0 | 0 | 0.111 |
| mmu-miR-1946b | 0 | 0 | 0 | 0 | 0 | 1 | 0 | 0 | 0 | 0 | 0 | 0.111 |
| mmu-miR-1947-5p | 45 | 35 | 27 | 26 | 40 | 14 | 5.121527 | 4.3829 | 3.136535 | 2.936805 | 4.457765 | 1.553994 |
| mmu-miR-1948-3p | 0 | 0 | 0 | 0 | 0 | 1 | 0 | 0 | 0 | 0 | 0 | 0.111 |
| mmu-miR-1948-5p | 5 | 4 | 7 | 8 | 7 | 4 | 0.569059 | 0.500903 | 0.813176 | 0.903632 | 0.780109 | 0.443998 |
| mmu-miR-1949 | 7 | 2 | 9 | 4 | 5 | 1 | 0.796682 | 0.250451 | 1.045512 | 0.451816 | 0.557221 | 0.111 |
| mmu-miR-1950 | 4 | 1 | 4 | 1 | 2 | 0 | 0.455247 | 0.125226 | 0.464672 | 0.112954 | 0.222888 | 0 |
| mmu-miR-1953 | 5 | 3 | 5 | 0 | 8 | 3 | 0.569059 | 0.375677 | 0.58084 | 0 | 0.891553 | 0.332999 |
| mmu-miR-1955-3p | 1 | 4 | 6 | 4 | 4 | 0 | 0.113812 | 0.500903 | 0.697008 | 0.451816 | 0.445777 | 0 |
| mmu-miR-1955-5p | 3 | 1 | 1 | 4 | 1 | 2 | 0.341435 | 0.125226 | 0.116168 | 0.451816 | 0.111444 | 0.221999 |
| mmu-miR-1956 | 1 | 0 | 2 | 1 | 0 | 0 | 0.113812 | 0 | 0.232336 | 0.112954 | 0 | 0 |
| mmu-miR-1958 | 0 | 0 | 5 | 1 | 0 | 0 | 0 | 0 | 0.58084 | 0.112954 | 0 | 0 |
| mmu-miR-195a-3p | 8 | 5 | 16 | 43 | 49 | 117 | 0.910494 | 0.626129 | 1.858687 | 4.857024 | 5.460762 | 12.98695 |
| mmu-miR-195a-5p | 35 | 32 | 37 | 271 | 270 | 971 | 3.98341 | 4.007223 | 4.298215 | 30.61055 | 30.08992 | 107.7806 |
| mmu-miR-1960 | 1 | 2 | 4 | 1 | 0 | 3 | 0.113812 | 0.250451 | 0.464672 | 0.112954 | 0 | 0.332999 |
| mmu-miR-1964-3p | 156 | 133 | 148 | 94 | 113 | 87 | 17.75463 | 16.65502 | 17.19286 | 10.61768 | 12.59319 | 9.656966 |
| mmu-miR-1966-3p | 0 | 0 | 0 | 0 | 0 | 1 | 0 | 0 | 0 | 0 | 0 | 0.111 |
| mmu-miR-1968-3p | 0 | 0 | 0 | 0 | 1 | 0 | 0 | 0 | 0 | 0 | 0.111444 | 0 |
| mmu-miR-1968-5p | 78 | 59 | 73 | 49 | 63 | 47 | 8.877313 | 7.388317 | 8.480262 | 5.534748 | 7.02098 | 5.216981 |
| mmu-miR-1969 | 0 | 2 | 1 | 0 | 0 | 0 | 0 | 0.250451 | 0.116168 | 0 | 0 | 0 |
| mmu-miR-196a-1-3p | 154 | 139 | 140 | 47 | 45 | 92 | 17.527 | 17.40637 | 16.26352 | 5.30884 | 5.014986 | 10.21196 |
| mmu-miR-196a-2-3p | 0 | 0 | 1 | 0 | 0 | 0 | 0 | 0 | 0.116168 | 0 | 0 | 0 |
| mmu-miR-196a-5p | 80 | 67 | 102 | 28 | 45 | 92 | 9.104937 | 8.390123 | 11.84913 | 3.162713 | 5.014986 | 10.21196 |
| mmu-miR-196b-5p | 2 | 0 | 1 | 1 | 0 | 2 | 0.227623 | 0 | 0.116168 | 0.112954 | 0 | 0.221999 |
| mmu-miR-1970 | 0 | 0 | 0 | 0 | 0 | 1 | 0 | 0 | 0 | 0 | 0 | 0.111 |
| mmu-miR-1970b-5p | 0 | 1 | 0 | 0 | 0 | 0 | 0 | 0.125226 | 0 | 0 | 0 | 0 |
| mmu-miR-1981-3p | 66 | 42 | 42 | 36 | 51 | 52 | 7.511573 | 5.25948 | 4.879055 | 4.066346 | 5.683651 | 5.77198 |
| mmu-miR-1981-5p | 985 | 1081 | 1060 | 721 | 764 | 645 | 112.1045 | 135.369 | 123.138 | 81.43987 | 85.14332 | 71.59475 |
| mmu-miR-1982-3p | 1 | 0 | 3 | 0 | 0 | 0 | 0.113812 | 0 | 0.348504 | 0 | 0 | 0 |
| mmu-miR-1983 | 97 | 82 | 104 | 53 | 54 | 52 | 11.03974 | 10.26851 | 12.08147 | 5.986564 | 6.017983 | 5.77198 |
| mmu-miR-199a-3p | 465 | 383 | 428 | 1600 | 1672 | 6760 | 52.92245 | 47.96145 | 49.71989 | 180.7265 | 186.3346 | 750.3573 |
| mmu-miR-199a-5p | 394 | 319 | 375 | 1754 | 1907 | 5706 | 44.84181 | 39.947 | 43.56299 | 198.1214 | 212.524 | 633.3638 |
| mmu-miR-199b-5p | 48 | 62 | 51 | 279 | 262 | 955 | 5.462962 | 7.763995 | 5.924566 | 31.51418 | 29.19836 | 106.0046 |
| mmu-miR-19a-3p | 397 | 370 | 336 | 554 | 471 | 382 | 45.18325 | 46.33352 | 39.03244 | 62.57654 | 52.49019 | 42.40185 |
| mmu-miR-19a-5p | 0 | 0 | 1 | 0 | 1 | 0 | 0 | 0 | 0.116168 | 0 | 0.111444 | 0 |
| mmu-miR-19b-1-5p | 0 | 0 | 0 | 2 | 3 | 3 | 0 | 0 | 0 | 0.225908 | 0.334332 | 0.332999 |
| mmu-miR-19b-3p | 2460 | 2137 | 2047 | 3647 | 3163 | 3103 | 279.9768 | 267.6074 | 237.7958 | 411.9434 | 352.4978 | 344.4318 |
| mmu-miR-1a-1-5p | 0 | 0 | 0 | 0 | 0 | 2 | 0 | 0 | 0 | 0 | 0 | 0.221999 |
| mmu-miR-1a-2-5p | 0 | 1 | 0 | 2 | 0 | 5 | 0 | 0.125226 | 0 | 0.225908 | 0 | 0.554998 |
| mmu-miR-1a-3p | 255 | 292 | 240 | 5035 | 5657 | 19181 | 29.02199 | 36.56591 | 27.88031 | 568.7236 | 630.4394 | 2129.083 |
| mmu-miR-1b-3p | 0 | 1 | 0 | 2 | 0 | 5 | 0 | 0.125226 | 0 | 0.225908 | 0 | 0.554998 |
| mmu-miR-1b-5p | 255 | 292 | 240 | 5031 | 5654 | 19163 | 29.02199 | 36.56591 | 27.88031 | 568.2718 | 630.1051 | 2127.085 |
| mmu-miR-200a-3p | 81696 | 73311 | 79985 | 133230 | 126150 | 89737 | 9297.962 | 9180.423 | 9291.695 | 15048.87 | 14058.68 | 9960.772 |
| mmu-miR-200a-5p | 8967 | 8285 | 8838 | 9557 | 10132 | 6869 | 1020.55 | 1037.495 | 1026.692 | 1079.502 | 1129.152 | 762.4563 |
| mmu-miR-200b-3p | 181460 | 168127 | 169233 | 208755 | 204928 | 131869 | 20652.27 | 21053.82 | 19659.45 | 23579.72 | 22838.02 | 14637.41 |
| mmu-miR-200b-5p | 684 | 667 | 638 | 661 | 776 | 626 | 77.84721 | 83.52555 | 74.11516 | 74.66262 | 86.48065 | 69.48575 |
| mmu-miR-200c-3p | 36686 | 34273 | 34454 | 35382 | 37412 | 33513 | 4175.296 | 4291.861 | 4002.451 | 3996.54 | 4169.348 | 3719.93 |
| mmu-miR-200c-5p | 41 | 34 | 47 | 34 | 22 | 34 | 4.66628 | 4.257674 | 5.459894 | 3.840438 | 2.451771 | 3.773987 |
| mmu-miR-203-3p | 58678 | 56439 | 59353 | 41798 | 44141 | 30561 | 6678.244 | 7067.614 | 6894.917 | 4721.253 | 4919.255 | 3392.259 |
| mmu-miR-203-5p | 312 | 329 | 304 | 161 | 168 | 187 | 35.50925 | 41.19926 | 35.31506 | 18.1856 | 18.72261 | 20.75693 |
| mmu-miR-203b-3p | 301 | 324 | 297 | 158 | 158 | 180 | 34.25733 | 40.57313 | 34.50189 | 17.84674 | 17.60817 | 19.97993 |
| mmu-miR-203b-5p | 4434 | 4154 | 5315 | 3778 | 4509 | 3380 | 504.6411 | 520.1876 | 617.4327 | 426.7404 | 502.5016 | 375.1787 |
| mmu-miR-204-5p | 1 | 2 | 2 | 25 | 13 | 63 | 0.113812 | 0.250451 | 0.232336 | 2.823851 | 1.448774 | 6.992975 |
| mmu-miR-205-5p | 161 | 0 | 7 | 7 | 15 | 16 | 18.32369 | 0 | 0.813176 | 0.790678 | 1.671662 | 1.775994 |
| mmu-miR-206-3p | 7 | 0 | 2 | 0 | 3 | 9 | 0.796682 | 0 | 0.232336 | 0 | 0.334332 | 0.998996 |
| mmu-miR-208b-3p | 0 | 0 | 1 | 0 | 0 | 1 | 0 | 0 | 0.116168 | 0 | 0 | 0.111 |
| mmu-miR-20a-3p | 4 | 4 | 3 | 7 | 3 | 3 | 0.455247 | 0.500903 | 0.348504 | 0.790678 | 0.334332 | 0.332999 |
| mmu-miR-20a-5p | 6078 | 5481 | 5787 | 6154 | 5963 | 5773 | 691.7476 | 686.3622 | 672.264 | 695.1192 | 664.5414 | 640.8007 |
| mmu-miR-20b-3p | 0 | 0 | 0 | 0 | 1 | 0 | 0 | 0 | 0 | 0 | 0.111444 | 0 |
| mmu-miR-20b-5p | 91 | 76 | 102 | 230 | 250 | 265 | 10.35687 | 9.517155 | 11.84913 | 25.97943 | 27.86103 | 29.4149 |
| mmu-miR-210-3p | 380 | 363 | 395 | 104 | 80 | 142 | 43.24845 | 45.45694 | 45.88635 | 11.74722 | 8.91553 | 15.76194 |
| mmu-miR-210-5p | 11 | 11 | 17 | 2 | 2 | 6 | 1.251929 | 1.377483 | 1.974855 | 0.225908 | 0.222888 | 0.665998 |
| mmu-miR-211-5p | 1 | 0 | 0 | 3 | 2 | 2 | 0.113812 | 0 | 0 | 0.338862 | 0.222888 | 0.221999 |
| mmu-miR-212-3p | 9 | 8 | 8 | 5 | 5 | 40 | 1.024305 | 1.001806 | 0.929344 | 0.56477 | 0.557221 | 4.439984 |
| mmu-miR-212-5p | 36 | 35 | 36 | 33 | 34 | 76 | 4.097222 | 4.3829 | 4.182047 | 3.727483 | 3.7891 | 8.43597 |
| mmu-miR-214-3p | 24 | 10 | 10 | 54 | 85 | 288 | 2.731481 | 1.252257 | 1.16168 | 6.099518 | 9.472751 | 31.96789 |
| mmu-miR-214-5p | 8 | 3 | 16 | 39 | 49 | 106 | 0.910494 | 0.375677 | 1.858687 | 4.405208 | 5.460762 | 11.76596 |
| mmu-miR-215-3p | 24215 | 21616 | 24195 | 21211 | 23983 | 17078 | 2755.951 | 2706.879 | 2810.684 | 2395.868 | 2672.765 | 1895.651 |
| mmu-miR-215-5p | 2046530 | 1847353 | 2109211 | 2277094 | 2345793 | 1439534 | 232919.1 | 231336.1 | 245022.8 | 257207 | 261424.9 | 159787.7 |
| mmu-miR-216b-5p | 2 | 2 | 0 | 0 | 0 | 0 | 0.227623 | 0.250451 | 0 | 0 | 0 | 0 |
| mmu-miR-217-5p | 1 | 2 | 1 | 0 | 1 | 4 | 0.113812 | 0.250451 | 0.116168 | 0 | 0.111444 | 0.443998 |
| mmu-miR-218-5p | 376 | 343 | 402 | 605 | 590 | 1235 | 42.7932 | 42.95242 | 46.69952 | 68.3372 | 65.75204 | 137.0845 |
| mmu-miR-219a-1-3p | 43 | 29 | 45 | 34 | 15 | 34 | 4.893904 | 3.631546 | 5.227559 | 3.840438 | 1.671662 | 3.773987 |
| mmu-miR-219a-2-3p | 4 | 3 | 4 | 1 | 3 | 3 | 0.455247 | 0.375677 | 0.464672 | 0.112954 | 0.334332 | 0.332999 |
| mmu-miR-219a-5p | 25 | 17 | 38 | 31 | 28 | 26 | 2.845293 | 2.128837 | 4.414383 | 3.501575 | 3.120436 | 2.88599 |
| mmu-miR-219b-3p | 4 | 0 | 0 | 1 | 3 | 2 | 0.455247 | 0 | 0 | 0.112954 | 0.334332 | 0.221999 |
| mmu-miR-219b-5p | 0 | 1 | 0 | 1 | 1 | 0 | 0 | 0.125226 | 0 | 0.112954 | 0.111444 | 0 |
| mmu-miR-219c-3p | 0 | 0 | 2 | 0 | 0 | 1 | 0 | 0 | 0.232336 | 0 | 0 | 0.111 |
| mmu-miR-219c-5p | 0 | 4 | 1 | 2 | 2 | 2 | 0 | 0.500903 | 0.116168 | 0.225908 | 0.222888 | 0.221999 |
| mmu-miR-21a-3p | 420 | 375 | 424 | 198 | 208 | 230 | 47.80092 | 46.95964 | 49.25522 | 22.3649 | 23.18038 | 25.52991 |
| mmu-miR-21a-5p | 4127224 | 3739162 | 3878096 | 2872322 | 2731036 | 2554973 | 469726.4 | 468239.3 | 450510.5 | 324440.4 | 304357.9 | 283601 |
| mmu-miR-221-3p | 206 | 185 | 168 | 203 | 235 | 416 | 23.44521 | 23.16676 | 19.51622 | 22.92967 | 26.18937 | 46.17584 |
| mmu-miR-221-5p | 32 | 29 | 30 | 42 | 43 | 74 | 3.641975 | 3.631546 | 3.485039 | 4.74407 | 4.792098 | 8.213971 |
| mmu-miR-222-3p | 141 | 82 | 110 | 110 | 96 | 169 | 16.04745 | 10.26851 | 12.77848 | 12.42494 | 10.69864 | 18.75893 |
| mmu-miR-222-5p | 5 | 7 | 7 | 3 | 6 | 6 | 0.569059 | 0.87658 | 0.813176 | 0.338862 | 0.668665 | 0.665998 |
| mmu-miR-223-3p | 15 | 12 | 8 | 42 | 33 | 154 | 1.707176 | 1.502709 | 0.929344 | 4.74407 | 3.677656 | 17.09394 |
| mmu-miR-223-5p | 7 | 6 | 9 | 23 | 19 | 71 | 0.796682 | 0.751354 | 1.045512 | 2.597943 | 2.117438 | 7.880972 |
| mmu-miR-22-3p | 17345 | 15153 | 17596 | 19749 | 20302 | 23984 | 1974.064 | 1897.545 | 2044.092 | 2230.729 | 2262.539 | 2662.215 |
| mmu-miR-224-3p | 0 | 1 | 0 | 0 | 2 | 1 | 0 | 0.125226 | 0 | 0 | 0.222888 | 0.111 |
| mmu-miR-224-5p | 195 | 150 | 227 | 379 | 422 | 391 | 22.19328 | 18.78386 | 26.37013 | 42.80958 | 47.02942 | 43.40085 |
| mmu-miR-22-5p | 208 | 154 | 209 | 165 | 185 | 213 | 23.67284 | 19.28476 | 24.27911 | 18.63742 | 20.61716 | 23.64292 |
| mmu-miR-23a-3p | 547 | 416 | 464 | 1206 | 1276 | 4428 | 62.25501 | 52.0939 | 53.90194 | 136.2226 | 142.2027 | 491.5063 |
| mmu-miR-23a-5p | 0 | 0 | 0 | 2 | 3 | 0 | 0 | 0 | 0 | 0.225908 | 0.334332 | 0 |
| mmu-miR-23b-3p | 696 | 633 | 674 | 1427 | 1371 | 2865 | 79.21295 | 79.26788 | 78.29721 | 161.1854 | 152.7899 | 318.0139 |
| mmu-miR-23b-5p | 0 | 3 | 4 | 5 | 3 | 2 | 0 | 0.375677 | 0.464672 | 0.56477 | 0.334332 | 0.221999 |
| mmu-miR-24-1-5p | 13 | 17 | 17 | 20 | 23 | 31 | 1.479552 | 2.128837 | 1.974855 | 2.259081 | 2.563215 | 3.440988 |
| mmu-miR-24-2-5p | 281 | 250 | 300 | 446 | 483 | 973 | 31.98109 | 31.30643 | 34.85039 | 50.3775 | 53.82752 | 108.0026 |
| mmu-miR-24-3p | 3962 | 3596 | 3924 | 5950 | 5618 | 11474 | 450.922 | 450.3117 | 455.8431 | 672.0766 | 626.0931 | 1273.609 |
| mmu-miR-25-3p | 3806 | 3567 | 4229 | 4499 | 5031 | 5688 | 433.1674 | 446.6801 | 491.2743 | 508.1802 | 560.6754 | 631.3658 |
| mmu-miR-25-5p | 24 | 7 | 16 | 20 | 14 | 7 | 2.731481 | 0.87658 | 1.858687 | 2.259081 | 1.560218 | 0.776997 |
| mmu-miR-26a-1-3p | 0 | 3 | 3 | 4 | 1 | 9 | 0 | 0.375677 | 0.348504 | 0.451816 | 0.111444 | 0.998996 |
| mmu-miR-26a-2-3p | 69 | 59 | 47 | 86 | 105 | 122 | 7.853008 | 7.388317 | 5.459894 | 9.714048 | 11.70163 | 13.54195 |
| mmu-miR-26a-5p | 35814 | 32413 | 33817 | 57759 | 63338 | 86235 | 4076.053 | 4058.941 | 3928.452 | 6524.113 | 7058.648 | 9572.051 |
| mmu-miR-26b-3p | 13 | 13 | 11 | 13 | 21 | 17 | 1.479552 | 1.627934 | 1.277848 | 1.468403 | 2.340327 | 1.886993 |
| mmu-miR-26b-5p | 2520 | 2474 | 2615 | 4069 | 4258 | 6875 | 286.8055 | 309.8084 | 303.7792 | 459.61 | 474.5291 | 763.1223 |
| mmu-miR-27a-3p | 1333 | 1030 | 1326 | 2048 | 2622 | 7005 | 151.711 | 128.9825 | 154.0387 | 231.3299 | 292.2065 | 777.5522 |
| mmu-miR-27a-5p | 734 | 741 | 779 | 455 | 502 | 598 | 83.5378 | 92.79226 | 90.49485 | 51.39409 | 55.94495 | 66.37776 |
| mmu-miR-27b-3p | 32533 | 29816 | 36820 | 38702 | 45396 | 61529 | 3702.636 | 3733.73 | 4277.305 | 4371.547 | 5059.118 | 6829.695 |
| mmu-miR-27b-5p | 78 | 79 | 88 | 76 | 72 | 90 | 8.877313 | 9.892832 | 10.22278 | 8.584507 | 8.023977 | 9.989965 |
| mmu-miR-28a-3p | 525 | 455 | 447 | 407 | 511 | 595 | 59.75115 | 56.9777 | 51.92708 | 45.9723 | 56.94795 | 66.04477 |
| mmu-miR-28a-5p | 628 | 591 | 691 | 586 | 644 | 922 | 71.47375 | 74.0084 | 80.27207 | 66.19107 | 71.77002 | 102.3416 |
| mmu-miR-292a-5p | 0 | 0 | 1 | 0 | 0 | 0 | 0 | 0 | 0.116168 | 0 | 0 | 0 |
| mmu-miR-293-5p | 2 | 0 | 0 | 0 | 0 | 0 | 0.227623 | 0 | 0 | 0 | 0 | 0 |
| mmu-miR-295-3p | 0 | 1 | 0 | 0 | 0 | 0 | 0 | 0.125226 | 0 | 0 | 0 | 0 |
| mmu-miR-296-3p | 75 | 57 | 92 | 29 | 51 | 46 | 8.535878 | 7.137866 | 10.68745 | 3.275667 | 5.683651 | 5.105982 |
| mmu-miR-297a-5p | 0 | 0 | 0 | 0 | 1 | 1 | 0 | 0 | 0 | 0 | 0.111444 | 0.111 |
| mmu-miR-297b-3p | 1 | 0 | 1 | 1 | 2 | 3 | 0.113812 | 0 | 0.116168 | 0.112954 | 0.222888 | 0.332999 |
| mmu-miR-297b-5p | 0 | 0 | 1 | 0 | 2 | 4 | 0 | 0 | 0.116168 | 0 | 0.222888 | 0.443998 |
| mmu-miR-298-5p | 66 | 79 | 102 | 48 | 29 | 54 | 7.511573 | 9.892832 | 11.84913 | 5.421794 | 3.23188 | 5.993979 |
| mmu-miR-299a-3p | 0 | 1 | 1 | 20 | 13 | 59 | 0 | 0.125226 | 0.116168 | 2.259081 | 1.448774 | 6.548977 |
| mmu-miR-299a-5p | 1 | 0 | 0 | 0 | 3 | 5 | 0.113812 | 0 | 0 | 0 | 0.334332 | 0.554998 |
| mmu-miR-29a-3p | 3153 | 2834 | 3146 | 4174 | 4215 | 8714 | 358.8483 | 354.8897 | 365.4644 | 471.4702 | 469.737 | 967.2506 |
| mmu-miR-29a-5p | 5 | 8 | 9 | 20 | 22 | 27 | 0.569059 | 1.001806 | 1.045512 | 2.259081 | 2.451771 | 2.996989 |
| mmu-miR-29b-1-5p | 2 | 6 | 2 | 6 | 3 | 6 | 0.227623 | 0.751354 | 0.232336 | 0.677724 | 0.334332 | 0.665998 |
| mmu-miR-29b-2-5p | 16 | 8 | 14 | 14 | 10 | 20 | 1.820987 | 1.001806 | 1.626352 | 1.581357 | 1.114441 | 2.219992 |
| mmu-miR-29b-3p | 186 | 156 | 153 | 284 | 216 | 471 | 21.16898 | 19.53521 | 17.7737 | 32.07895 | 24.07193 | 52.28081 |
| mmu-miR-29c-3p | 57 | 54 | 50 | 100 | 97 | 227 | 6.487268 | 6.762189 | 5.808398 | 11.2954 | 10.81008 | 25.19691 |
| mmu-miR-29c-5p | 116 | 123 | 107 | 175 | 175 | 204 | 13.20216 | 15.40276 | 12.42997 | 19.76696 | 19.50272 | 22.64392 |
| mmu-miR-300-3p | 5 | 6 | 9 | 71 | 73 | 282 | 0.569059 | 0.751354 | 1.045512 | 8.019737 | 8.135422 | 31.30189 |
| mmu-miR-300-5p | 0 | 0 | 0 | 0 | 1 | 8 | 0 | 0 | 0 | 0 | 0.111444 | 0.887997 |
| mmu-miR-301a-3p | 6 | 8 | 3 | 6 | 8 | 13 | 0.68287 | 1.001806 | 0.348504 | 0.677724 | 0.891553 | 1.442995 |
| mmu-miR-301a-5p | 29 | 32 | 31 | 19 | 23 | 21 | 3.30054 | 4.007223 | 3.601207 | 2.146127 | 2.563215 | 2.330992 |
| mmu-miR-301b-3p | 0 | 2 | 5 | 8 | 1 | 1 | 0 | 0.250451 | 0.58084 | 0.903632 | 0.111444 | 0.111 |
| mmu-miR-301b-5p | 1 | 6 | 6 | 1 | 3 | 1 | 0.113812 | 0.751354 | 0.697008 | 0.112954 | 0.334332 | 0.111 |
| mmu-miR-3057-3p | 2 | 0 | 1 | 0 | 0 | 0 | 0.227623 | 0 | 0.116168 | 0 | 0 | 0 |
| mmu-miR-3057-5p | 4 | 10 | 10 | 2 | 3 | 4 | 0.455247 | 1.252257 | 1.16168 | 0.225908 | 0.334332 | 0.443998 |
| mmu-miR-3058-3p | 0 | 0 | 2 | 0 | 0 | 0 | 0 | 0 | 0.232336 | 0 | 0 | 0 |
| mmu-miR-3058-5p | 3 | 0 | 0 | 0 | 0 | 0 | 0.341435 | 0 | 0 | 0 | 0 | 0 |
| mmu-miR-3060-3p | 0 | 1 | 0 | 0 | 0 | 0 | 0 | 0.125226 | 0 | 0 | 0 | 0 |
| mmu-miR-3061-3p | 2 | 1 | 0 | 0 | 0 | 1 | 0.227623 | 0.125226 | 0 | 0 | 0 | 0.111 |
| mmu-miR-3061-5p | 6 | 2 | 2 | 5 | 2 | 4 | 0.68287 | 0.250451 | 0.232336 | 0.56477 | 0.222888 | 0.443998 |
| mmu-miR-3062-5p | 0 | 1 | 1 | 0 | 1 | 0 | 0 | 0.125226 | 0.116168 | 0 | 0.111444 | 0 |
| mmu-miR-3064-3p | 0 | 0 | 0 | 1 | 1 | 2 | 0 | 0 | 0 | 0.112954 | 0.111444 | 0.221999 |
| mmu-miR-3064-5p | 7 | 3 | 4 | 3 | 4 | 7 | 0.796682 | 0.375677 | 0.464672 | 0.338862 | 0.445777 | 0.776997 |
| mmu-miR-3065-3p | 1 | 0 | 0 | 2 | 7 | 27 | 0.113812 | 0 | 0 | 0.225908 | 0.780109 | 2.996989 |
| mmu-miR-3065-5p | 0 | 0 | 0 | 5 | 8 | 30 | 0 | 0 | 0 | 0.56477 | 0.891553 | 3.329988 |
| mmu-miR-3066-3p | 0 | 0 | 2 | 0 | 3 | 0 | 0 | 0 | 0.232336 | 0 | 0.334332 | 0 |
| mmu-miR-3066-5p | 25 | 22 | 20 | 14 | 13 | 25 | 2.845293 | 2.754966 | 2.323359 | 1.581357 | 1.448774 | 2.77499 |
| mmu-miR-3068-3p | 52 | 31 | 52 | 66 | 62 | 107 | 5.918209 | 3.881997 | 6.040734 | 7.454967 | 6.909536 | 11.87696 |
| mmu-miR-3068-5p | 57 | 40 | 51 | 114 | 111 | 183 | 6.487268 | 5.009029 | 5.924566 | 12.87676 | 12.3703 | 20.31293 |
| mmu-miR-3069-3p | 0 | 0 | 0 | 1 | 0 | 2 | 0 | 0 | 0 | 0.112954 | 0 | 0.221999 |
| mmu-miR-3071-3p | 0 | 0 | 4 | 15 | 30 | 64 | 0 | 0 | 0.464672 | 1.694311 | 3.343324 | 7.103975 |
| mmu-miR-3071-5p | 13 | 7 | 6 | 108 | 135 | 420 | 1.479552 | 0.87658 | 0.697008 | 12.19904 | 15.04496 | 46.61983 |
| mmu-miR-3072-3p | 1 | 0 | 0 | 0 | 0 | 1 | 0.113812 | 0 | 0 | 0 | 0 | 0.111 |
| mmu-miR-3073a-3p | 6 | 3 | 6 | 9 | 1 | 1 | 0.68287 | 0.375677 | 0.697008 | 1.016586 | 0.111444 | 0.111 |
| mmu-miR-3073a-5p | 1 | 3 | 4 | 2 | 1 | 1 | 0.113812 | 0.375677 | 0.464672 | 0.225908 | 0.111444 | 0.111 |
| mmu-miR-3073b-3p | 1 | 3 | 4 | 2 | 1 | 1 | 0.113812 | 0.375677 | 0.464672 | 0.225908 | 0.111444 | 0.111 |
| mmu-miR-3073b-5p | 2 | 2 | 5 | 7 | 0 | 1 | 0.227623 | 0.250451 | 0.58084 | 0.790678 | 0 | 0.111 |
| mmu-miR-3074-1-3p | 12 | 10 | 12 | 17 | 19 | 26 | 1.365741 | 1.252257 | 1.394016 | 1.920219 | 2.117438 | 2.88599 |
| mmu-miR-3074-2-3p | 276 | 248 | 297 | 436 | 481 | 962 | 31.41203 | 31.05598 | 34.50189 | 49.24796 | 53.60463 | 106.7816 |
| mmu-miR-3074-5p | 3950 | 3587 | 3920 | 5940 | 5613 | 11459 | 449.5563 | 449.1847 | 455.3784 | 670.947 | 625.5359 | 1271.944 |
| mmu-miR-3075-5p | 0 | 0 | 1 | 0 | 0 | 0 | 0 | 0 | 0.116168 | 0 | 0 | 0 |
| mmu-miR-3076-3p | 1 | 2 | 6 | 1 | 1 | 2 | 0.113812 | 0.250451 | 0.697008 | 0.112954 | 0.111444 | 0.221999 |
| mmu-miR-3076-5p | 0 | 0 | 1 | 0 | 0 | 0 | 0 | 0 | 0.116168 | 0 | 0 | 0 |
| mmu-miR-3079-3p | 0 | 0 | 1 | 2 | 0 | 0 | 0 | 0 | 0.116168 | 0.225908 | 0 | 0 |
| mmu-miR-3079-5p | 1 | 2 | 2 | 2 | 1 | 3 | 0.113812 | 0.250451 | 0.232336 | 0.225908 | 0.111444 | 0.332999 |
| mmu-miR-3081-3p | 14 | 12 | 16 | 50 | 37 | 50 | 1.593364 | 1.502709 | 1.858687 | 5.647702 | 4.123433 | 5.54998 |
| mmu-miR-3082-3p | 0 | 0 | 1 | 2 | 2 | 0 | 0 | 0 | 0.116168 | 0.225908 | 0.222888 | 0 |
| mmu-miR-3082-5p | 0 | 0 | 0 | 1 | 0 | 0 | 0 | 0 | 0 | 0.112954 | 0 | 0 |
| mmu-miR-3084-3p | 3 | 5 | 0 | 0 | 4 | 4 | 0.341435 | 0.626129 | 0 | 0 | 0.445777 | 0.443998 |
| mmu-miR-3084-5p | 2 | 3 | 1 | 1 | 2 | 5 | 0.227623 | 0.375677 | 0.116168 | 0.112954 | 0.222888 | 0.554998 |
| mmu-miR-3086-5p | 1 | 0 | 0 | 0 | 0 | 1 | 0.113812 | 0 | 0 | 0 | 0 | 0.111 |
| mmu-miR-3091-3p | 1 | 4 | 2 | 1 | 1 | 1 | 0.113812 | 0.500903 | 0.232336 | 0.112954 | 0.111444 | 0.111 |
| mmu-miR-3092-3p | 1 | 0 | 0 | 1 | 0 | 0 | 0.113812 | 0 | 0 | 0.112954 | 0 | 0 |
| mmu-miR-3094-3p | 0 | 1 | 0 | 0 | 0 | 0 | 0 | 0.125226 | 0 | 0 | 0 | 0 |
| mmu-miR-3095-3p | 6 | 1 | 6 | 8 | 12 | 3 | 0.68287 | 0.125226 | 0.697008 | 0.903632 | 1.33733 | 0.332999 |
| mmu-miR-3095-5p | 1 | 5 | 3 | 6 | 2 | 2 | 0.113812 | 0.626129 | 0.348504 | 0.677724 | 0.222888 | 0.221999 |
| mmu-miR-3098-3p | 7 | 4 | 1 | 0 | 2 | 4 | 0.796682 | 0.500903 | 0.116168 | 0 | 0.222888 | 0.443998 |
| mmu-miR-3098-5p | 5 | 4 | 1 | 0 | 0 | 3 | 0.569059 | 0.500903 | 0.116168 | 0 | 0 | 0.332999 |
| mmu-miR-30a-3p | 181 | 185 | 179 | 301 | 353 | 546 | 20.59992 | 23.16676 | 20.79407 | 33.99917 | 39.33978 | 60.60578 |
| mmu-miR-30a-5p | 22265 | 20378 | 19951 | 38546 | 37250 | 53098 | 2534.018 | 2551.85 | 2317.667 | 4353.927 | 4151.294 | 5893.857 |
| mmu-miR-30b-3p | 222 | 172 | 190 | 194 | 217 | 216 | 25.2662 | 21.53882 | 22.07191 | 21.91308 | 24.18338 | 23.97591 |
| mmu-miR-30b-5p | 1137 | 1103 | 1103 | 2254 | 1999 | 3820 | 129.4039 | 138.124 | 128.1333 | 254.5984 | 222.7768 | 424.0185 |
| mmu-miR-30c-1-3p | 95 | 101 | 97 | 72 | 96 | 91 | 10.81211 | 12.6478 | 11.26829 | 8.132691 | 10.69864 | 10.10096 |
| mmu-miR-30c-2-3p | 109 | 105 | 85 | 112 | 144 | 221 | 12.40548 | 13.1487 | 9.874277 | 12.65085 | 16.04795 | 24.53091 |
| mmu-miR-30c-5p | 5451 | 5182 | 5364 | 10260 | 9687 | 13733 | 620.3876 | 648.9197 | 623.125 | 1158.908 | 1079.559 | 1524.358 |
| mmu-miR-30d-3p | 81 | 80 | 114 | 97 | 119 | 127 | 9.218749 | 10.01806 | 13.24315 | 10.95654 | 13.26185 | 14.09695 |
| mmu-miR-30d-5p | 43579 | 40915 | 39828 | 60913 | 59906 | 52951 | 4959.801 | 5123.61 | 4626.738 | 6880.37 | 6676.172 | 5877.54 |
| mmu-miR-30e-3p | 641 | 631 | 622 | 729 | 724 | 691 | 72.95331 | 79.01743 | 72.25648 | 82.3435 | 80.68555 | 76.70073 |
| mmu-miR-30e-5p | 40460 | 37189 | 37270 | 51677 | 46465 | 44503 | 4604.822 | 4657.019 | 4329.58 | 5837.126 | 5178.252 | 4939.815 |
| mmu-miR-30f | 84 | 77 | 61 | 105 | 101 | 170 | 9.560184 | 9.64238 | 7.086246 | 11.86017 | 11.25586 | 18.86993 |
| mmu-miR-3101-5p | 2 | 0 | 0 | 0 | 0 | 0 | 0.227623 | 0 | 0 | 0 | 0 | 0 |
| mmu-miR-3102-3p | 0 | 0 | 0 | 2 | 2 | 9 | 0 | 0 | 0 | 0.225908 | 0.222888 | 0.998996 |
| mmu-miR-3102-5p.2-5p | 0 | 0 | 0 | 0 | 1 | 0 | 0 | 0 | 0 | 0 | 0.111444 | 0 |
| mmu-miR-3103-3p | 2 | 0 | 4 | 1 | 2 | 0 | 0.227623 | 0 | 0.464672 | 0.112954 | 0.222888 | 0 |
| mmu-miR-3105-3p | 16 | 13 | 23 | 25 | 25 | 20 | 1.820987 | 1.627934 | 2.671863 | 2.823851 | 2.786103 | 2.219992 |
| mmu-miR-3105-5p | 1 | 1 | 3 | 5 | 7 | 9 | 0.113812 | 0.125226 | 0.348504 | 0.56477 | 0.780109 | 0.998996 |
| mmu-miR-3109-3p | 5 | 9 | 5 | 8 | 5 | 3 | 0.569059 | 1.127031 | 0.58084 | 0.903632 | 0.557221 | 0.332999 |
| mmu-miR-3109-5p | 0 | 2 | 3 | 2 | 2 | 1 | 0 | 0.250451 | 0.348504 | 0.225908 | 0.222888 | 0.111 |
| mmu-miR-3110-5p | 1 | 2 | 1 | 3 | 2 | 4 | 0.113812 | 0.250451 | 0.116168 | 0.338862 | 0.222888 | 0.443998 |
| mmu-miR-3112-3p | 0 | 0 | 1 | 0 | 0 | 0 | 0 | 0 | 0.116168 | 0 | 0 | 0 |
| mmu-miR-3112-5p | 1 | 0 | 0 | 0 | 0 | 0 | 0.113812 | 0 | 0 | 0 | 0 | 0 |
| mmu-miR-31-3p | 144 | 170 | 141 | 252 | 186 | 309 | 16.38889 | 21.28837 | 16.37968 | 28.46442 | 20.72861 | 34.29888 |
| mmu-miR-31-5p | 1607 | 1644 | 1661 | 3050 | 2868 | 2309 | 182.8954 | 205.8711 | 192.955 | 344.5098 | 319.6218 | 256.2981 |
| mmu-miR-320-3p | 636 | 603 | 581 | 704 | 731 | 776 | 72.38425 | 75.51111 | 67.49359 | 79.51965 | 81.46566 | 86.13569 |
| mmu-miR-320-5p | 0 | 0 | 1 | 0 | 0 | 1 | 0 | 0 | 0.116168 | 0 | 0 | 0.111 |
| mmu-miR-322-3p | 4 | 5 | 3 | 38 | 61 | 223 | 0.455247 | 0.626129 | 0.348504 | 4.292254 | 6.798092 | 24.75291 |
| mmu-miR-322-5p | 0 | 2 | 7 | 25 | 25 | 116 | 0 | 0.250451 | 0.813176 | 2.823851 | 2.786103 | 12.87595 |
| mmu-miR-323-3p | 1 | 0 | 2 | 7 | 7 | 16 | 0.113812 | 0 | 0.232336 | 0.790678 | 0.780109 | 1.775994 |
| mmu-miR-32-3p | 40 | 36 | 47 | 32 | 51 | 42 | 4.552468 | 4.508126 | 5.459894 | 3.614529 | 5.683651 | 4.661983 |
| mmu-miR-324-3p | 4 | 0 | 2 | 1 | 1 | 5 | 0.455247 | 0 | 0.232336 | 0.112954 | 0.111444 | 0.554998 |
| mmu-miR-324-5p | 13 | 4 | 12 | 16 | 15 | 17 | 1.479552 | 0.500903 | 1.394016 | 1.807265 | 1.671662 | 1.886993 |
| mmu-miR-325-3p | 1 | 0 | 0 | 2 | 0 | 2 | 0.113812 | 0 | 0 | 0.225908 | 0 | 0.221999 |
| mmu-miR-325-5p | 0 | 0 | 0 | 0 | 0 | 2 | 0 | 0 | 0 | 0 | 0 | 0.221999 |
| mmu-miR-32-5p | 599 | 592 | 643 | 917 | 794 | 666 | 68.17322 | 74.13363 | 74.696 | 103.5789 | 88.48664 | 73.92574 |
| mmu-miR-326-3p | 14 | 6 | 11 | 21 | 32 | 57 | 1.593364 | 0.751354 | 1.277848 | 2.372035 | 3.566212 | 6.326978 |
| mmu-miR-328-3p | 110 | 81 | 119 | 118 | 124 | 291 | 12.51929 | 10.14328 | 13.82399 | 13.32858 | 13.81907 | 32.30089 |
| mmu-miR-328-5p | 0 | 0 | 0 | 0 | 0 | 1 | 0 | 0 | 0 | 0 | 0 | 0.111 |
| mmu-miR-329-3p | 0 | 0 | 1 | 4 | 0 | 13 | 0 | 0 | 0.116168 | 0.451816 | 0 | 1.442995 |
| mmu-miR-329-5p | 0 | 0 | 0 | 7 | 14 | 31 | 0 | 0 | 0 | 0.790678 | 1.560218 | 3.440988 |
| mmu-miR-330-3p | 11 | 18 | 12 | 17 | 18 | 41 | 1.251929 | 2.254063 | 1.394016 | 1.920219 | 2.005994 | 4.550984 |
| mmu-miR-330-5p | 45 | 50 | 43 | 60 | 38 | 83 | 5.121527 | 6.261286 | 4.995223 | 6.777243 | 4.234877 | 9.212967 |
| mmu-miR-331-3p | 3 | 9 | 3 | 13 | 14 | 30 | 0.341435 | 1.127031 | 0.348504 | 1.468403 | 1.560218 | 3.329988 |
| mmu-miR-331-5p | 14 | 8 | 4 | 8 | 9 | 16 | 1.593364 | 1.001806 | 0.464672 | 0.903632 | 1.002997 | 1.775994 |
| mmu-miR-33-3p | 5 | 4 | 8 | 4 | 6 | 14 | 0.569059 | 0.500903 | 0.929344 | 0.451816 | 0.668665 | 1.553994 |
| mmu-miR-335-3p | 4 | 2 | 1 | 18 | 8 | 65 | 0.455247 | 0.250451 | 0.116168 | 2.033173 | 0.891553 | 7.214974 |
| mmu-miR-335-5p | 2 | 3 | 0 | 15 | 17 | 45 | 0.227623 | 0.375677 | 0 | 1.694311 | 1.89455 | 4.994982 |
| mmu-miR-33-5p | 63 | 59 | 88 | 55 | 65 | 74 | 7.170138 | 7.388317 | 10.22278 | 6.212472 | 7.243869 | 8.213971 |
| mmu-miR-337-3p | 0 | 0 | 0 | 0 | 0 | 1 | 0 | 0 | 0 | 0 | 0 | 0.111 |
| mmu-miR-337-5p | 2 | 1 | 0 | 10 | 6 | 28 | 0.227623 | 0.125226 | 0 | 1.12954 | 0.668665 | 3.107989 |
| mmu-miR-338-3p | 0 | 0 | 0 | 5 | 8 | 30 | 0 | 0 | 0 | 0.56477 | 0.891553 | 3.329988 |
| mmu-miR-338-5p | 1 | 0 | 0 | 3 | 8 | 28 | 0.113812 | 0 | 0 | 0.338862 | 0.891553 | 3.107989 |
| mmu-miR-339-3p | 20 | 10 | 12 | 21 | 14 | 51 | 2.276234 | 1.252257 | 1.394016 | 2.372035 | 1.560218 | 5.66098 |
| mmu-miR-339-5p | 87 | 59 | 99 | 128 | 133 | 252 | 9.901619 | 7.388317 | 11.50063 | 14.45812 | 14.82207 | 27.9719 |
| mmu-miR-340-3p | 94 | 51 | 74 | 99 | 103 | 138 | 10.6983 | 6.386512 | 8.59643 | 11.18245 | 11.47875 | 15.31795 |
| mmu-miR-340-5p | 2910 | 2919 | 2976 | 4175 | 4476 | 7808 | 331.1921 | 365.5339 | 345.7159 | 471.5831 | 498.8239 | 866.6849 |
| mmu-miR-341-3p | 6 | 3 | 2 | 27 | 25 | 128 | 0.68287 | 0.375677 | 0.232336 | 3.049759 | 2.786103 | 14.20795 |
| mmu-miR-342-3p | 51 | 45 | 32 | 178 | 181 | 538 | 5.804397 | 5.635157 | 3.717375 | 20.10582 | 20.17139 | 59.71779 |
| mmu-miR-342-5p | 1 | 0 | 2 | 13 | 8 | 16 | 0.113812 | 0 | 0.232336 | 1.468403 | 0.891553 | 1.775994 |
| mmu-miR-344-3p | 3 | 5 | 0 | 17 | 11 | 31 | 0.341435 | 0.626129 | 0 | 1.920219 | 1.225885 | 3.440988 |
| mmu-miR-344b-3p | 0 | 0 | 1 | 0 | 0 | 0 | 0 | 0 | 0.116168 | 0 | 0 | 0 |
| mmu-miR-344d-3p | 0 | 0 | 0 | 0 | 2 | 4 | 0 | 0 | 0 | 0 | 0.222888 | 0.443998 |
| mmu-miR-344e-3p | 0 | 0 | 1 | 0 | 0 | 0 | 0 | 0 | 0.116168 | 0 | 0 | 0 |
| mmu-miR-345-3p | 15 | 11 | 19 | 14 | 16 | 36 | 1.707176 | 1.377483 | 2.207191 | 1.581357 | 1.783106 | 3.995986 |
| mmu-miR-345-5p | 27 | 17 | 24 | 51 | 48 | 89 | 3.072916 | 2.128837 | 2.788031 | 5.760656 | 5.349318 | 9.878965 |
| mmu-miR-3470a | 4 | 0 | 1 | 1 | 1 | 5 | 0.455247 | 0 | 0.116168 | 0.112954 | 0.111444 | 0.554998 |
| mmu-miR-3470b | 24 | 13 | 22 | 13 | 3 | 15 | 2.731481 | 1.627934 | 2.555695 | 1.468403 | 0.334332 | 1.664994 |
| mmu-miR-3473a | 0 | 0 | 1 | 0 | 0 | 0 | 0 | 0 | 0.116168 | 0 | 0 | 0 |
| mmu-miR-3473b | 4 | 7 | 4 | 1 | 1 | 3 | 0.455247 | 0.87658 | 0.464672 | 0.112954 | 0.111444 | 0.332999 |
| mmu-miR-3473d | 6 | 11 | 3 | 0 | 7 | 1 | 0.68287 | 1.377483 | 0.348504 | 0 | 0.780109 | 0.111 |
| mmu-miR-3473e | 2 | 0 | 2 | 0 | 0 | 1 | 0.227623 | 0 | 0.232336 | 0 | 0 | 0.111 |
| mmu-miR-3473g | 1 | 0 | 0 | 0 | 0 | 0 | 0.113812 | 0 | 0 | 0 | 0 | 0 |
| mmu-miR-3473h-5p | 13 | 17 | 15 | 4 | 6 | 6 | 1.479552 | 2.128837 | 1.74252 | 0.451816 | 0.668665 | 0.665998 |
| mmu-miR-3474 | 2 | 0 | 0 | 3 | 9 | 1 | 0.227623 | 0 | 0 | 0.338862 | 1.002997 | 0.111 |
| mmu-miR-34a-5p | 186 | 209 | 224 | 53 | 46 | 137 | 21.16898 | 26.17218 | 26.02162 | 5.986564 | 5.12643 | 15.20695 |
| mmu-miR-34b-3p | 4 | 2 | 2 | 0 | 0 | 5 | 0.455247 | 0.250451 | 0.232336 | 0 | 0 | 0.554998 |
| mmu-miR-34b-5p | 0 | 0 | 0 | 1 | 1 | 4 | 0 | 0 | 0 | 0.112954 | 0.111444 | 0.443998 |
| mmu-miR-34c-3p | 0 | 0 | 1 | 0 | 2 | 2 | 0 | 0 | 0.116168 | 0 | 0.222888 | 0.221999 |
| mmu-miR-34c-5p | 72 | 47 | 75 | 177 | 215 | 272 | 8.194443 | 5.885609 | 8.712598 | 19.99287 | 23.96049 | 30.19189 |
| mmu-miR-350-3p | 12 | 3 | 10 | 18 | 20 | 67 | 1.365741 | 0.375677 | 1.16168 | 2.033173 | 2.228883 | 7.436974 |
| mmu-miR-350-5p | 1 | 0 | 1 | 5 | 2 | 9 | 0.113812 | 0 | 0.116168 | 0.56477 | 0.222888 | 0.998996 |
| mmu-miR-351-3p | 0 | 0 | 0 | 0 | 1 | 1 | 0 | 0 | 0 | 0 | 0.111444 | 0.111 |
| mmu-miR-351-5p | 2 | 3 | 2 | 7 | 13 | 38 | 0.227623 | 0.375677 | 0.232336 | 0.790678 | 1.448774 | 4.217985 |
| mmu-miR-3535 | 245 | 317 | 262 | 350 | 285 | 419 | 27.88387 | 39.69655 | 30.43601 | 39.53392 | 31.76158 | 46.50883 |
| mmu-miR-3544-5p | 0 | 0 | 0 | 0 | 0 | 1 | 0 | 0 | 0 | 0 | 0 | 0.111 |
| mmu-miR-3572-3p | 0 | 0 | 0 | 1 | 0 | 3 | 0 | 0 | 0 | 0.112954 | 0 | 0.332999 |
| mmu-miR-361-3p | 1187 | 1108 | 1015 | 1192 | 1266 | 1158 | 135.0945 | 138.7501 | 117.9105 | 134.6412 | 141.0883 | 128.5375 |
| mmu-miR-361-5p | 113 | 103 | 135 | 176 | 159 | 213 | 12.86072 | 12.89825 | 15.68268 | 19.87991 | 17.71962 | 23.64292 |
| mmu-miR-362-3p | 53 | 41 | 58 | 98 | 83 | 194 | 6.032021 | 5.134254 | 6.737742 | 11.0695 | 9.249863 | 21.53392 |
| mmu-miR-362-5p | 186 | 202 | 173 | 161 | 158 | 182 | 21.16898 | 25.2956 | 20.09706 | 18.1856 | 17.60817 | 20.20193 |
| mmu-miR-363-3p | 105 | 64 | 118 | 165 | 208 | 141 | 11.95023 | 8.014446 | 13.70782 | 18.63742 | 23.18038 | 15.65094 |
| mmu-miR-363-5p | 12 | 14 | 7 | 15 | 39 | 16 | 1.365741 | 1.75316 | 0.813176 | 1.694311 | 4.346321 | 1.775994 |
| mmu-miR-365-1-5p | 0 | 0 | 0 | 1 | 1 | 2 | 0 | 0 | 0 | 0.112954 | 0.111444 | 0.221999 |
| mmu-miR-365-2-5p | 2 | 5 | 3 | 2 | 2 | 1 | 0.227623 | 0.626129 | 0.348504 | 0.225908 | 0.222888 | 0.111 |
| mmu-miR-365-3p | 12 | 3 | 8 | 48 | 29 | 159 | 1.365741 | 0.375677 | 0.929344 | 5.421794 | 3.23188 | 17.64894 |
| mmu-miR-369-3p | 1 | 0 | 0 | 8 | 4 | 45 | 0.113812 | 0 | 0 | 0.903632 | 0.445777 | 4.994982 |
| mmu-miR-369-5p | 2 | 0 | 0 | 13 | 8 | 26 | 0.227623 | 0 | 0 | 1.468403 | 0.891553 | 2.88599 |
| mmu-miR-370-3p | 4 | 3 | 3 | 14 | 24 | 61 | 0.455247 | 0.375677 | 0.348504 | 1.581357 | 2.674659 | 6.770976 |
| mmu-miR-370-5p | 0 | 0 | 0 | 0 | 1 | 0 | 0 | 0 | 0 | 0 | 0.111444 | 0 |
| mmu-miR-374b-5p | 1135 | 1045 | 1081 | 1544 | 1722 | 2223 | 129.1763 | 130.8609 | 125.5776 | 174.401 | 191.9068 | 246.7521 |
| mmu-miR-374c-3p | 1129 | 1045 | 1079 | 1541 | 1718 | 2220 | 128.4934 | 130.8609 | 125.3452 | 174.0622 | 191.461 | 246.4191 |
| mmu-miR-374c-5p | 1 | 1 | 0 | 0 | 1 | 1 | 0.113812 | 0.125226 | 0 | 0 | 0.111444 | 0.111 |
| mmu-miR-375-3p | 8771 | 8082 | 9452 | 16909 | 19881 | 14701 | 998.2425 | 1012.074 | 1098.02 | 1909.94 | 2215.621 | 1631.805 |
| mmu-miR-375-5p | 0 | 1 | 0 | 0 | 0 | 0 | 0 | 0.125226 | 0 | 0 | 0 | 0 |
| mmu-miR-376a-3p | 0 | 0 | 0 | 1 | 0 | 1 | 0 | 0 | 0 | 0.112954 | 0 | 0.111 |
| mmu-miR-376a-5p | 0 | 0 | 0 | 0 | 1 | 2 | 0 | 0 | 0 | 0 | 0.111444 | 0.221999 |
| mmu-miR-376b-3p | 0 | 0 | 0 | 1 | 0 | 9 | 0 | 0 | 0 | 0.112954 | 0 | 0.998996 |
| mmu-miR-376b-5p | 0 | 0 | 0 | 3 | 1 | 14 | 0 | 0 | 0 | 0.338862 | 0.111444 | 1.553994 |
| mmu-miR-376c-3p | 0 | 0 | 0 | 10 | 10 | 28 | 0 | 0 | 0 | 1.12954 | 1.114441 | 3.107989 |
| mmu-miR-377-3p | 0 | 0 | 0 | 1 | 0 | 6 | 0 | 0 | 0 | 0.112954 | 0 | 0.665998 |
| mmu-miR-377-5p | 0 | 0 | 0 | 0 | 0 | 1 | 0 | 0 | 0 | 0 | 0 | 0.111 |
| mmu-miR-378a-3p | 89276 | 74791 | 89979 | 71362 | 84806 | 80617 | 10160.65 | 9365.757 | 10452.68 | 8060.627 | 9451.131 | 8948.455 |
| mmu-miR-378a-5p | 952 | 857 | 915 | 828 | 825 | 899 | 108.3487 | 107.3184 | 106.2937 | 93.52595 | 91.94141 | 99.78865 |
| mmu-miR-378b | 39 | 30 | 65 | 34 | 36 | 84 | 4.438657 | 3.756772 | 7.550918 | 3.840438 | 4.011989 | 9.323967 |
| mmu-miR-378c | 3884 | 3075 | 4607 | 4003 | 4720 | 5642 | 442.0447 | 385.0691 | 535.1858 | 452.155 | 526.0163 | 626.2598 |
| mmu-miR-378d | 0 | 5 | 1 | 1 | 0 | 0 | 0 | 0.626129 | 0.116168 | 0.112954 | 0 | 0 |
| mmu-miR-379-3p | 0 | 0 | 0 | 0 | 4 | 9 | 0 | 0 | 0 | 0 | 0.445777 | 0.998996 |
| mmu-miR-379-5p | 35 | 26 | 24 | 199 | 246 | 892 | 3.98341 | 3.255869 | 2.788031 | 22.47785 | 27.41526 | 99.01165 |
| mmu-miR-380-3p | 0 | 0 | 0 | 0 | 1 | 5 | 0 | 0 | 0 | 0 | 0.111444 | 0.554998 |
| mmu-miR-380-5p | 0 | 0 | 0 | 8 | 4 | 11 | 0 | 0 | 0 | 0.903632 | 0.445777 | 1.220996 |
| mmu-miR-381-3p | 34 | 15 | 17 | 235 | 291 | 864 | 3.869598 | 1.878386 | 1.974855 | 26.5442 | 32.43024 | 95.90366 |
| mmu-miR-382-3p | 1 | 1 | 3 | 14 | 11 | 72 | 0.113812 | 0.125226 | 0.348504 | 1.581357 | 1.225885 | 7.991972 |
| mmu-miR-382-5p | 2 | 1 | 1 | 11 | 10 | 54 | 0.227623 | 0.125226 | 0.116168 | 1.242494 | 1.114441 | 5.993979 |
| mmu-miR-383-5p | 0 | 0 | 0 | 0 | 0 | 1 | 0 | 0 | 0 | 0 | 0 | 0.111 |
| mmu-miR-384-3p | 3 | 0 | 0 | 3 | 4 | 9 | 0.341435 | 0 | 0 | 0.338862 | 0.445777 | 0.998996 |
| mmu-miR-384-5p | 0 | 0 | 1 | 2 | 1 | 12 | 0 | 0 | 0.116168 | 0.225908 | 0.111444 | 1.331995 |
| mmu-miR-3968 | 0 | 0 | 0 | 1 | 0 | 0 | 0 | 0 | 0 | 0.112954 | 0 | 0 |
| mmu-miR-409-3p | 9 | 2 | 5 | 21 | 28 | 65 | 1.024305 | 0.250451 | 0.58084 | 2.372035 | 3.120436 | 7.214974 |
| mmu-miR-409-5p | 2 | 0 | 1 | 7 | 12 | 36 | 0.227623 | 0 | 0.116168 | 0.790678 | 1.33733 | 3.995986 |
| mmu-miR-410-3p | 0 | 0 | 0 | 8 | 14 | 37 | 0 | 0 | 0 | 0.903632 | 1.560218 | 4.106985 |
| mmu-miR-410-5p | 0 | 0 | 0 | 0 | 0 | 2 | 0 | 0 | 0 | 0 | 0 | 0.221999 |
| mmu-miR-411-3p | 2 | 0 | 3 | 8 | 11 | 28 | 0.227623 | 0 | 0.348504 | 0.903632 | 1.225885 | 3.107989 |
| mmu-miR-411-5p | 29 | 20 | 23 | 298 | 352 | 1132 | 3.30054 | 2.504514 | 2.671863 | 33.66031 | 39.22833 | 125.6516 |
| mmu-miR-412-5p | 0 | 1 | 1 | 1 | 0 | 8 | 0 | 0.125226 | 0.116168 | 0.112954 | 0 | 0.887997 |
| mmu-miR-421-3p | 33 | 37 | 34 | 31 | 49 | 42 | 3.755786 | 4.633352 | 3.949711 | 3.501575 | 5.460762 | 4.661983 |
| mmu-miR-423-3p | 2628 | 2400 | 2502 | 1763 | 1974 | 1766 | 299.0972 | 300.5417 | 290.6523 | 199.138 | 219.9907 | 196.0253 |
| mmu-miR-423-5p | 1035 | 936 | 948 | 929 | 1036 | 1118 | 117.7951 | 117.2113 | 110.1272 | 104.9343 | 115.4561 | 124.0976 |
| mmu-miR-425-3p | 192 | 168 | 197 | 260 | 349 | 342 | 21.85185 | 21.03792 | 22.88509 | 29.36805 | 38.894 | 37.96187 |
| mmu-miR-425-5p | 3202 | 2867 | 2973 | 3903 | 3516 | 3017 | 364.4251 | 359.0221 | 345.3674 | 440.8596 | 391.8376 | 334.8858 |
| mmu-miR-429-3p | 13059 | 11899 | 12690 | 14595 | 14974 | 12041 | 1486.267 | 1490.061 | 1474.172 | 1648.564 | 1668.764 | 1336.546 |
| mmu-miR-429-5p | 0 | 0 | 0 | 2 | 0 | 0 | 0 | 0 | 0 | 0.225908 | 0 | 0 |
| mmu-miR-431-3p | 0 | 0 | 0 | 0 | 1 | 8 | 0 | 0 | 0 | 0 | 0.111444 | 0.887997 |
| mmu-miR-431-5p | 0 | 0 | 0 | 0 | 2 | 7 | 0 | 0 | 0 | 0 | 0.222888 | 0.776997 |
| mmu-miR-433-3p | 3 | 0 | 0 | 14 | 10 | 27 | 0.341435 | 0 | 0 | 1.581357 | 1.114441 | 2.996989 |
| mmu-miR-433-5p | 0 | 0 | 0 | 0 | 0 | 1 | 0 | 0 | 0 | 0 | 0 | 0.111 |
| mmu-miR-434-3p | 7 | 4 | 3 | 21 | 47 | 174 | 0.796682 | 0.500903 | 0.348504 | 2.372035 | 5.237874 | 19.31393 |
| mmu-miR-434-5p | 19 | 7 | 17 | 216 | 226 | 594 | 2.162423 | 0.87658 | 1.974855 | 24.39807 | 25.18637 | 65.93377 |
| mmu-miR-448-3p | 0 | 0 | 0 | 0 | 0 | 1 | 0 | 0 | 0 | 0 | 0 | 0.111 |
| mmu-miR-449a-3p | 1 | 0 | 0 | 0 | 0 | 1 | 0.113812 | 0 | 0 | 0 | 0 | 0.111 |
| mmu-miR-449a-5p | 29 | 24 | 28 | 7 | 8 | 12 | 3.30054 | 3.005417 | 3.252703 | 0.790678 | 0.891553 | 1.331995 |
| mmu-miR-449c-5p | 1 | 3 | 3 | 0 | 0 | 0 | 0.113812 | 0.375677 | 0.348504 | 0 | 0 | 0 |
| mmu-miR-450a-1-3p | 0 | 0 | 0 | 0 | 0 | 1 | 0 | 0 | 0 | 0 | 0 | 0.111 |
| mmu-miR-450a-5p | 6 | 3 | 13 | 97 | 109 | 468 | 0.68287 | 0.375677 | 1.510184 | 10.95654 | 12.14741 | 51.94782 |
| mmu-miR-450b-3p | 0 | 0 | 2 | 0 | 1 | 7 | 0 | 0 | 0.232336 | 0 | 0.111444 | 0.776997 |
| mmu-miR-450b-5p | 1 | 0 | 0 | 13 | 18 | 33 | 0.113812 | 0 | 0 | 1.468403 | 2.005994 | 3.662987 |
| mmu-miR-451a | 475 | 97 | 116 | 269 | 297 | 910 | 54.06056 | 12.14689 | 13.47548 | 30.38464 | 33.09891 | 101.0096 |
| mmu-miR-452-3p | 0 | 0 | 0 | 0 | 2 | 1 | 0 | 0 | 0 | 0 | 0.222888 | 0.111 |
| mmu-miR-452-5p | 3 | 1 | 1 | 1 | 4 | 2 | 0.341435 | 0.125226 | 0.116168 | 0.112954 | 0.445777 | 0.221999 |
| mmu-miR-455-3p | 7 | 5 | 5 | 40 | 26 | 152 | 0.796682 | 0.626129 | 0.58084 | 4.518162 | 2.897547 | 16.87194 |
| mmu-miR-455-5p | 116 | 91 | 81 | 588 | 555 | 1830 | 13.20216 | 11.39554 | 9.409605 | 66.41698 | 61.85149 | 203.1293 |
| mmu-miR-465a-3p | 0 | 1 | 0 | 0 | 0 | 0 | 0 | 0.125226 | 0 | 0 | 0 | 0 |
| mmu-miR-466a-3p | 0 | 1 | 2 | 8 | 6 | 6 | 0 | 0.125226 | 0.232336 | 0.903632 | 0.668665 | 0.665998 |
| mmu-miR-466a-5p | 0 | 0 | 0 | 0 | 0 | 4 | 0 | 0 | 0 | 0 | 0 | 0.443998 |
| mmu-miR-466b-3p | 0 | 1 | 2 | 8 | 6 | 6 | 0 | 0.125226 | 0.232336 | 0.903632 | 0.668665 | 0.665998 |
| mmu-miR-466b-5p | 4 | 1 | 1 | 3 | 7 | 12 | 0.455247 | 0.125226 | 0.116168 | 0.338862 | 0.780109 | 1.331995 |
| mmu-miR-466c-5p | 1 | 1 | 1 | 12 | 11 | 17 | 0.113812 | 0.125226 | 0.116168 | 1.355449 | 1.225885 | 1.886993 |
| mmu-miR-466d-3p | 2 | 0 | 3 | 4 | 2 | 6 | 0.227623 | 0 | 0.348504 | 0.451816 | 0.222888 | 0.665998 |
| mmu-miR-466d-5p | 0 | 0 | 0 | 1 | 2 | 0 | 0 | 0 | 0 | 0.112954 | 0.222888 | 0 |
| mmu-miR-466e-5p | 1 | 0 | 0 | 1 | 2 | 8 | 0.113812 | 0 | 0 | 0.112954 | 0.222888 | 0.887997 |
| mmu-miR-466f | 0 | 0 | 1 | 1 | 0 | 0 | 0 | 0 | 0.116168 | 0.112954 | 0 | 0 |
| mmu-miR-466f-3p | 0 | 0 | 0 | 0 | 1 | 2 | 0 | 0 | 0 | 0 | 0.111444 | 0.221999 |
| mmu-miR-466f-5p | 0 | 0 | 1 | 1 | 0 | 0 | 0 | 0 | 0.116168 | 0.112954 | 0 | 0 |
| mmu-miR-466h-3p | 0 | 1 | 0 | 0 | 1 | 2 | 0 | 0.125226 | 0 | 0 | 0.111444 | 0.221999 |
| mmu-miR-466h-5p | 0 | 0 | 0 | 1 | 1 | 5 | 0 | 0 | 0 | 0.112954 | 0.111444 | 0.554998 |
| mmu-miR-466i-3p | 0 | 0 | 0 | 0 | 1 | 1 | 0 | 0 | 0 | 0 | 0.111444 | 0.111 |
| mmu-miR-466i-5p | 5 | 4 | 8 | 4 | 5 | 8 | 0.569059 | 0.500903 | 0.929344 | 0.451816 | 0.557221 | 0.887997 |
| mmu-miR-466k | 3 | 1 | 0 | 2 | 2 | 2 | 0.341435 | 0.125226 | 0 | 0.225908 | 0.222888 | 0.221999 |
| mmu-miR-466n-5p | 0 | 0 | 0 | 1 | 2 | 0 | 0 | 0 | 0 | 0.112954 | 0.222888 | 0 |
| mmu-miR-466p-5p | 3 | 1 | 0 | 2 | 4 | 7 | 0.341435 | 0.125226 | 0 | 0.225908 | 0.445777 | 0.776997 |
| mmu-miR-466q | 2 | 5 | 6 | 1 | 1 | 1 | 0.227623 | 0.626129 | 0.697008 | 0.112954 | 0.111444 | 0.111 |
| mmu-miR-467a-3p | 0 | 2 | 2 | 4 | 7 | 12 | 0 | 0.250451 | 0.232336 | 0.451816 | 0.780109 | 1.331995 |
| mmu-miR-467a-5p | 41 | 34 | 31 | 160 | 132 | 223 | 4.66628 | 4.257674 | 3.601207 | 18.07265 | 14.71063 | 24.75291 |
| mmu-miR-467b-5p | 41 | 34 | 31 | 160 | 132 | 223 | 4.66628 | 4.257674 | 3.601207 | 18.07265 | 14.71063 | 24.75291 |
| mmu-miR-467c-3p | 0 | 0 | 0 | 0 | 0 | 1 | 0 | 0 | 0 | 0 | 0 | 0.111 |
| mmu-miR-467c-5p | 0 | 3 | 5 | 7 | 11 | 10 | 0 | 0.375677 | 0.58084 | 0.790678 | 1.225885 | 1.109996 |
| mmu-miR-467d-3p | 0 | 2 | 2 | 4 | 7 | 12 | 0 | 0.250451 | 0.232336 | 0.451816 | 0.780109 | 1.331995 |
| mmu-miR-467d-5p | 0 | 0 | 0 | 5 | 5 | 3 | 0 | 0 | 0 | 0.56477 | 0.557221 | 0.332999 |
| mmu-miR-467e-3p | 0 | 0 | 0 | 0 | 0 | 1 | 0 | 0 | 0 | 0 | 0 | 0.111 |
| mmu-miR-467e-5p | 1 | 4 | 1 | 28 | 15 | 37 | 0.113812 | 0.500903 | 0.116168 | 3.162713 | 1.671662 | 4.106985 |
| mmu-miR-470-5p | 1 | 0 | 0 | 0 | 0 | 0 | 0.113812 | 0 | 0 | 0 | 0 | 0 |
| mmu-miR-483-3p | 2 | 0 | 0 | 1 | 1 | 2 | 0.227623 | 0 | 0 | 0.112954 | 0.111444 | 0.221999 |
| mmu-miR-483-5p | 2 | 0 | 1 | 1 | 0 | 0 | 0.227623 | 0 | 0.116168 | 0.112954 | 0 | 0 |
| mmu-miR-484 | 450 | 443 | 457 | 417 | 448 | 478 | 51.21527 | 55.47499 | 53.08876 | 47.10184 | 49.92697 | 53.05781 |
| mmu-miR-485-3p | 0 | 1 | 1 | 1 | 2 | 17 | 0 | 0.125226 | 0.116168 | 0.112954 | 0.222888 | 1.886993 |
| mmu-miR-485-5p | 1 | 0 | 1 | 0 | 3 | 17 | 0.113812 | 0 | 0.116168 | 0 | 0.334332 | 1.886993 |
| mmu-miR-486a-3p | 25 | 18 | 30 | 34 | 50 | 159 | 2.845293 | 2.254063 | 3.485039 | 3.840438 | 5.572207 | 17.64894 |
| mmu-miR-486a-5p | 24 | 18 | 28 | 31 | 43 | 150 | 2.731481 | 2.254063 | 3.252703 | 3.501575 | 4.792098 | 16.64994 |
| mmu-miR-486b-3p | 24 | 18 | 28 | 30 | 42 | 150 | 2.731481 | 2.254063 | 3.252703 | 3.388621 | 4.680653 | 16.64994 |
| mmu-miR-487b-3p | 0 | 0 | 0 | 1 | 2 | 7 | 0 | 0 | 0 | 0.112954 | 0.222888 | 0.776997 |
| mmu-miR-488-3p | 2 | 0 | 2 | 2 | 4 | 18 | 0.227623 | 0 | 0.232336 | 0.225908 | 0.445777 | 1.997993 |
| mmu-miR-490-3p | 8 | 9 | 8 | 171 | 135 | 593 | 0.910494 | 1.127031 | 0.929344 | 19.31514 | 15.04496 | 65.82277 |
| mmu-miR-490-5p | 0 | 0 | 1 | 3 | 0 | 11 | 0 | 0 | 0.116168 | 0.338862 | 0 | 1.220996 |
| mmu-miR-491-5p | 0 | 0 | 0 | 0 | 0 | 2 | 0 | 0 | 0 | 0 | 0 | 0.221999 |
| mmu-miR-493-3p | 0 | 0 | 0 | 3 | 0 | 0 | 0 | 0 | 0 | 0.338862 | 0 | 0 |
| mmu-miR-493-5p | 2 | 1 | 1 | 2 | 0 | 3 | 0.227623 | 0.125226 | 0.116168 | 0.225908 | 0 | 0.332999 |
| mmu-miR-494-3p | 0 | 0 | 1 | 4 | 7 | 8 | 0 | 0 | 0.116168 | 0.451816 | 0.780109 | 0.887997 |
| mmu-miR-495-3p | 0 | 4 | 2 | 18 | 14 | 48 | 0 | 0.500903 | 0.232336 | 2.033173 | 1.560218 | 5.327981 |
| mmu-miR-496a-3p | 1 | 0 | 0 | 0 | 1 | 1 | 0.113812 | 0 | 0 | 0 | 0.111444 | 0.111 |
| mmu-miR-497a-3p | 0 | 0 | 0 | 0 | 1 | 1 | 0 | 0 | 0 | 0 | 0.111444 | 0.111 |
| mmu-miR-497a-5p | 5 | 11 | 14 | 48 | 41 | 226 | 0.569059 | 1.377483 | 1.626352 | 5.421794 | 4.569209 | 25.08591 |
| mmu-miR-497b | 4 | 4 | 5 | 3 | 3 | 8 | 0.455247 | 0.500903 | 0.58084 | 0.338862 | 0.334332 | 0.887997 |
| mmu-miR-499-5p | 4 | 6 | 0 | 0 | 1 | 3 | 0.455247 | 0.751354 | 0 | 0 | 0.111444 | 0.332999 |
| mmu-miR-500-3p | 12 | 15 | 17 | 18 | 18 | 46 | 1.365741 | 1.878386 | 1.974855 | 2.033173 | 2.005994 | 5.105982 |
| mmu-miR-500-5p | 1 | 0 | 3 | 1 | 1 | 0 | 0.113812 | 0 | 0.348504 | 0.112954 | 0.111444 | 0 |
| mmu-miR-501-3p | 199 | 181 | 200 | 117 | 137 | 171 | 22.64853 | 22.66586 | 23.23359 | 13.21562 | 15.26785 | 18.98093 |
| mmu-miR-501-5p | 4 | 5 | 7 | 1 | 2 | 9 | 0.455247 | 0.626129 | 0.813176 | 0.112954 | 0.222888 | 0.998996 |
| mmu-miR-503-3p | 0 | 0 | 0 | 3 | 3 | 7 | 0 | 0 | 0 | 0.338862 | 0.334332 | 0.776997 |
| mmu-miR-503-5p | 0 | 0 | 2 | 7 | 9 | 18 | 0 | 0 | 0.232336 | 0.790678 | 1.002997 | 1.997993 |
| mmu-miR-504-5p | 0 | 0 | 0 | 3 | 3 | 10 | 0 | 0 | 0 | 0.338862 | 0.334332 | 1.109996 |
| mmu-miR-505-3p | 4 | 1 | 3 | 4 | 1 | 7 | 0.455247 | 0.125226 | 0.348504 | 0.451816 | 0.111444 | 0.776997 |
| mmu-miR-505-5p | 0 | 4 | 1 | 2 | 0 | 2 | 0 | 0.500903 | 0.116168 | 0.225908 | 0 | 0.221999 |
| mmu-miR-5099 | 56 | 37 | 54 | 21 | 20 | 10 | 6.373456 | 4.633352 | 6.27307 | 2.372035 | 2.228883 | 1.109996 |
| mmu-miR-5101 | 0 | 2 | 0 | 0 | 0 | 0 | 0 | 0.250451 | 0 | 0 | 0 | 0 |
| mmu-miR-5103 | 2 | 1 | 3 | 0 | 0 | 3 | 0.227623 | 0.125226 | 0.348504 | 0 | 0 | 0.332999 |
| mmu-miR-5104 | 6 | 2 | 1 | 6 | 5 | 5 | 0.68287 | 0.250451 | 0.116168 | 0.677724 | 0.557221 | 0.554998 |
| mmu-miR-5107-3p | 4 | 0 | 1 | 1 | 1 | 5 | 0.455247 | 0 | 0.116168 | 0.112954 | 0.111444 | 0.554998 |
| mmu-miR-5107-5p | 5 | 3 | 6 | 0 | 4 | 6 | 0.569059 | 0.375677 | 0.697008 | 0 | 0.445777 | 0.665998 |
| mmu-miR-5113 | 7 | 6 | 9 | 5 | 7 | 6 | 0.796682 | 0.751354 | 1.045512 | 0.56477 | 0.780109 | 0.665998 |
| mmu-miR-511-3p | 0 | 1 | 0 | 5 | 2 | 17 | 0 | 0.125226 | 0 | 0.56477 | 0.222888 | 1.886993 |
| mmu-miR-5114 | 16 | 20 | 22 | 6 | 7 | 12 | 1.820987 | 2.504514 | 2.555695 | 0.677724 | 0.780109 | 1.331995 |
| mmu-miR-511-5p | 0 | 0 | 0 | 0 | 0 | 5 | 0 | 0 | 0 | 0 | 0 | 0.554998 |
| mmu-miR-5116 | 2 | 0 | 3 | 0 | 0 | 2 | 0.227623 | 0 | 0.348504 | 0 | 0 | 0.221999 |
| mmu-miR-5121 | 7 | 9 | 10 | 5 | 12 | 21 | 0.796682 | 1.127031 | 1.16168 | 0.56477 | 1.33733 | 2.330992 |
| mmu-miR-5122 | 0 | 0 | 0 | 1 | 1 | 6 | 0 | 0 | 0 | 0.112954 | 0.111444 | 0.665998 |
| mmu-miR-5123 | 3 | 7 | 4 | 6 | 1 | 2 | 0.341435 | 0.87658 | 0.464672 | 0.677724 | 0.111444 | 0.221999 |
| mmu-miR-5125 | 3 | 1 | 2 | 1 | 0 | 2 | 0.341435 | 0.125226 | 0.232336 | 0.112954 | 0 | 0.221999 |
| mmu-miR-5126 | 0 | 0 | 0 | 1 | 1 | 2 | 0 | 0 | 0 | 0.112954 | 0.111444 | 0.221999 |
| mmu-miR-5128 | 3 | 3 | 2 | 1 | 0 | 0 | 0.341435 | 0.375677 | 0.232336 | 0.112954 | 0 | 0 |
| mmu-miR-5129-3p | 2 | 2 | 0 | 1 | 0 | 5 | 0.227623 | 0.250451 | 0 | 0.112954 | 0 | 0.554998 |
| mmu-miR-5130 | 0 | 0 | 0 | 0 | 0 | 1 | 0 | 0 | 0 | 0 | 0 | 0.111 |
| mmu-miR-5132-3p | 0 | 0 | 1 | 0 | 0 | 0 | 0 | 0 | 0.116168 | 0 | 0 | 0 |
| mmu-miR-5132-5p | 2 | 1 | 1 | 2 | 2 | 0 | 0.227623 | 0.125226 | 0.116168 | 0.225908 | 0.222888 | 0 |
| mmu-miR-5135 | 0 | 0 | 0 | 1 | 0 | 0 | 0 | 0 | 0 | 0.112954 | 0 | 0 |
| mmu-miR-532-3p | 16 | 20 | 19 | 16 | 19 | 26 | 1.820987 | 2.504514 | 2.207191 | 1.807265 | 2.117438 | 2.88599 |
| mmu-miR-532-5p | 3272 | 2678 | 3262 | 2553 | 2815 | 3514 | 372.3919 | 335.3545 | 378.9399 | 288.3717 | 313.7152 | 390.0526 |
| mmu-miR-539-5p | 0 | 0 | 0 | 1 | 0 | 4 | 0 | 0 | 0 | 0.112954 | 0 | 0.443998 |
| mmu-miR-540-3p | 1 | 0 | 1 | 1 | 1 | 11 | 0.113812 | 0 | 0.116168 | 0.112954 | 0.111444 | 1.220996 |
| mmu-miR-540-5p | 0 | 0 | 0 | 2 | 0 | 2 | 0 | 0 | 0 | 0.225908 | 0 | 0.221999 |
| mmu-miR-541-5p | 4 | 13 | 12 | 82 | 100 | 249 | 0.455247 | 1.627934 | 1.394016 | 9.262232 | 11.14441 | 27.6389 |
| mmu-miR-542-3p | 9 | 5 | 5 | 39 | 42 | 139 | 1.024305 | 0.626129 | 0.58084 | 4.405208 | 4.680653 | 15.42895 |
| mmu-miR-543-3p | 1 | 3 | 1 | 3 | 3 | 8 | 0.113812 | 0.375677 | 0.116168 | 0.338862 | 0.334332 | 0.887997 |
| mmu-miR-543-5p | 0 | 0 | 0 | 3 | 1 | 0 | 0 | 0 | 0 | 0.338862 | 0.111444 | 0 |
| mmu-miR-5615-3p | 2 | 5 | 9 | 2 | 2 | 4 | 0.227623 | 0.626129 | 1.045512 | 0.225908 | 0.222888 | 0.443998 |
| mmu-miR-5615-5p | 3 | 6 | 8 | 2 | 2 | 4 | 0.341435 | 0.751354 | 0.929344 | 0.225908 | 0.222888 | 0.443998 |
| mmu-miR-5616-3p | 0 | 0 | 0 | 0 | 3 | 0 | 0 | 0 | 0 | 0 | 0.334332 | 0 |
| mmu-miR-5616-5p | 1 | 0 | 0 | 1 | 0 | 1 | 0.113812 | 0 | 0 | 0.112954 | 0 | 0.111 |
| mmu-miR-5620-3p | 0 | 0 | 2 | 0 | 1 | 0 | 0 | 0 | 0.232336 | 0 | 0.111444 | 0 |
| mmu-miR-5620-5p | 5 | 2 | 4 | 2 | 1 | 0 | 0.569059 | 0.250451 | 0.464672 | 0.225908 | 0.111444 | 0 |
| mmu-miR-5621-3p | 0 | 0 | 2 | 0 | 1 | 0 | 0 | 0 | 0.232336 | 0 | 0.111444 | 0 |
| mmu-miR-5624-3p | 0 | 2 | 1 | 2 | 2 | 1 | 0 | 0.250451 | 0.116168 | 0.225908 | 0.222888 | 0.111 |
| mmu-miR-5624-5p | 0 | 2 | 1 | 2 | 2 | 1 | 0 | 0.250451 | 0.116168 | 0.225908 | 0.222888 | 0.111 |
| mmu-miR-5625-3p | 0 | 0 | 0 | 0 | 0 | 1 | 0 | 0 | 0 | 0 | 0 | 0.111 |
| mmu-miR-5709-5p | 1 | 0 | 0 | 0 | 0 | 0 | 0.113812 | 0 | 0 | 0 | 0 | 0 |
| mmu-miR-574-3p | 73 | 65 | 78 | 95 | 88 | 193 | 8.308255 | 8.139672 | 9.061101 | 10.73063 | 9.807084 | 21.42292 |
| mmu-miR-574-5p | 138 | 129 | 101 | 107 | 104 | 145 | 15.70602 | 16.15412 | 11.73296 | 12.08608 | 11.59019 | 16.09494 |
| mmu-miR-582-3p | 517 | 441 | 484 | 372 | 384 | 397 | 58.84065 | 55.22454 | 56.2253 | 42.0189 | 42.79455 | 44.06684 |
| mmu-miR-582-5p | 89 | 63 | 77 | 77 | 69 | 87 | 10.12924 | 7.88922 | 8.944933 | 8.697461 | 7.689645 | 9.656966 |
| mmu-miR-592-3p | 0 | 0 | 0 | 1 | 0 | 0 | 0 | 0 | 0 | 0.112954 | 0 | 0 |
| mmu-miR-592-5p | 10 | 11 | 7 | 52 | 61 | 288 | 1.138117 | 1.377483 | 0.813176 | 5.87361 | 6.798092 | 31.96789 |
| mmu-miR-598-3p | 13 | 17 | 10 | 39 | 39 | 108 | 1.479552 | 2.128837 | 1.16168 | 4.405208 | 4.346321 | 11.98796 |
| mmu-miR-615-3p | 14 | 20 | 14 | 45 | 53 | 142 | 1.593364 | 2.504514 | 1.626352 | 5.082932 | 5.906539 | 15.76194 |
| mmu-miR-6399 | 9 | 7 | 18 | 1 | 1 | 9 | 1.024305 | 0.87658 | 2.091023 | 0.112954 | 0.111444 | 0.998996 |
| mmu-miR-6418-3p | 0 | 0 | 0 | 0 | 0 | 1 | 0 | 0 | 0 | 0 | 0 | 0.111 |
| mmu-miR-6516-3p | 2 | 0 | 0 | 0 | 0 | 0 | 0.227623 | 0 | 0 | 0 | 0 | 0 |
| mmu-miR-6516-5p | 0 | 3 | 2 | 1 | 2 | 5 | 0 | 0.375677 | 0.232336 | 0.112954 | 0.222888 | 0.554998 |
| mmu-miR-652-3p | 265 | 242 | 235 | 249 | 276 | 461 | 30.1601 | 30.30462 | 27.29947 | 28.12556 | 30.75858 | 51.17082 |
| mmu-miR-652-5p | 7 | 6 | 12 | 18 | 14 | 21 | 0.796682 | 0.751354 | 1.394016 | 2.033173 | 1.560218 | 2.330992 |
| mmu-miR-653-5p | 0 | 0 | 0 | 0 | 0 | 1 | 0 | 0 | 0 | 0 | 0 | 0.111 |
| mmu-miR-6537-3p | 0 | 0 | 1 | 0 | 0 | 1 | 0 | 0 | 0.116168 | 0 | 0 | 0.111 |
| mmu-miR-6538 | 1 | 0 | 2 | 0 | 0 | 1 | 0.113812 | 0 | 0.232336 | 0 | 0 | 0.111 |
| mmu-miR-6539 | 137 | 131 | 156 | 36 | 46 | 56 | 15.5922 | 16.40457 | 18.1222 | 4.066346 | 5.12643 | 6.215978 |
| mmu-miR-6540-5p | 0 | 0 | 1 | 1 | 3 | 2 | 0 | 0 | 0.116168 | 0.112954 | 0.334332 | 0.221999 |
| mmu-miR-654-3p | 0 | 0 | 0 | 1 | 0 | 0 | 0 | 0 | 0 | 0.112954 | 0 | 0 |
| mmu-miR-6546-3p | 0 | 0 | 1 | 0 | 0 | 0 | 0 | 0 | 0.116168 | 0 | 0 | 0 |
| mmu-miR-6546-5p | 2 | 3 | 3 | 0 | 1 | 0 | 0.227623 | 0.375677 | 0.348504 | 0 | 0.111444 | 0 |
| mmu-miR-664-3p | 5 | 4 | 6 | 7 | 22 | 35 | 0.569059 | 0.500903 | 0.697008 | 0.790678 | 2.451771 | 3.884986 |
| mmu-miR-664-5p | 15 | 15 | 6 | 8 | 10 | 5 | 1.707176 | 1.878386 | 0.697008 | 0.903632 | 1.114441 | 0.554998 |
| mmu-miR-665-3p | 0 | 0 | 0 | 0 | 0 | 1 | 0 | 0 | 0 | 0 | 0 | 0.111 |
| mmu-miR-666-5p | 0 | 0 | 0 | 4 | 0 | 4 | 0 | 0 | 0 | 0.451816 | 0 | 0.443998 |
| mmu-miR-667-3p | 0 | 0 | 0 | 0 | 2 | 1 | 0 | 0 | 0 | 0 | 0.222888 | 0.111 |
| mmu-miR-667-5p | 0 | 0 | 0 | 0 | 0 | 2 | 0 | 0 | 0 | 0 | 0 | 0.221999 |
| mmu-miR-668-3p | 0 | 0 | 0 | 1 | 1 | 4 | 0 | 0 | 0 | 0.112954 | 0.111444 | 0.443998 |
| mmu-miR-669a-3p | 5 | 4 | 2 | 14 | 11 | 27 | 0.569059 | 0.500903 | 0.232336 | 1.581357 | 1.225885 | 2.996989 |
| mmu-miR-669a-5p | 10 | 2 | 3 | 12 | 17 | 35 | 1.138117 | 0.250451 | 0.348504 | 1.355449 | 1.89455 | 3.884986 |
| mmu-miR-669b-3p | 0 | 0 | 0 | 1 | 0 | 0 | 0 | 0 | 0 | 0.112954 | 0 | 0 |
| mmu-miR-669b-5p | 0 | 0 | 0 | 5 | 0 | 4 | 0 | 0 | 0 | 0.56477 | 0 | 0.443998 |
| mmu-miR-669c-3p | 0 | 1 | 0 | 0 | 0 | 0 | 0 | 0.125226 | 0 | 0 | 0 | 0 |
| mmu-miR-669c-5p | 2 | 6 | 3 | 16 | 18 | 21 | 0.227623 | 0.751354 | 0.348504 | 1.807265 | 2.005994 | 2.330992 |
| mmu-miR-669d-5p | 0 | 0 | 2 | 2 | 4 | 8 | 0 | 0 | 0.232336 | 0.225908 | 0.445777 | 0.887997 |
| mmu-miR-669e-5p | 0 | 0 | 0 | 0 | 0 | 3 | 0 | 0 | 0 | 0 | 0 | 0.332999 |
| mmu-miR-669f-3p | 1 | 0 | 0 | 0 | 2 | 1 | 0.113812 | 0 | 0 | 0 | 0.222888 | 0.111 |
| mmu-miR-669f-5p | 0 | 2 | 0 | 2 | 2 | 5 | 0 | 0.250451 | 0 | 0.225908 | 0.222888 | 0.554998 |
| mmu-miR-669l-5p | 1 | 1 | 2 | 3 | 5 | 3 | 0.113812 | 0.125226 | 0.232336 | 0.338862 | 0.557221 | 0.332999 |
| mmu-miR-669m-5p | 0 | 0 | 0 | 2 | 1 | 2 | 0 | 0 | 0 | 0.225908 | 0.111444 | 0.221999 |
| mmu-miR-669o-5p | 3 | 1 | 3 | 3 | 7 | 15 | 0.341435 | 0.125226 | 0.348504 | 0.338862 | 0.780109 | 1.664994 |
| mmu-miR-671-3p | 20 | 21 | 36 | 30 | 18 | 38 | 2.276234 | 2.62974 | 4.182047 | 3.388621 | 2.005994 | 4.217985 |
| mmu-miR-671-5p | 12 | 17 | 14 | 27 | 16 | 29 | 1.365741 | 2.128837 | 1.626352 | 3.049759 | 1.783106 | 3.218989 |
| mmu-miR-672-5p | 2 | 10 | 4 | 5 | 3 | 4 | 0.227623 | 1.252257 | 0.464672 | 0.56477 | 0.334332 | 0.443998 |
| mmu-miR-673-3p | 0 | 1 | 0 | 1 | 0 | 0 | 0 | 0.125226 | 0 | 0.112954 | 0 | 0 |
| mmu-miR-673-5p | 0 | 0 | 0 | 0 | 0 | 1 | 0 | 0 | 0 | 0 | 0 | 0.111 |
| mmu-miR-674-3p | 358 | 287 | 360 | 269 | 309 | 400 | 40.74459 | 35.93978 | 41.82047 | 30.38464 | 34.43624 | 44.39984 |
| mmu-miR-674-5p | 32 | 23 | 18 | 28 | 33 | 48 | 3.641975 | 2.880192 | 2.091023 | 3.162713 | 3.677656 | 5.327981 |
| mmu-miR-676-3p | 5 | 4 | 0 | 12 | 24 | 46 | 0.569059 | 0.500903 | 0 | 1.355449 | 2.674659 | 5.105982 |
| mmu-miR-676-5p | 7 | 3 | 4 | 21 | 25 | 50 | 0.796682 | 0.375677 | 0.464672 | 2.372035 | 2.786103 | 5.54998 |
| mmu-miR-677-3p | 5 | 3 | 2 | 6 | 3 | 2 | 0.569059 | 0.375677 | 0.232336 | 0.677724 | 0.334332 | 0.221999 |
| mmu-miR-677-5p | 215 | 245 | 220 | 231 | 197 | 117 | 24.46952 | 30.6803 | 25.55695 | 26.09238 | 21.95449 | 12.98695 |
| mmu-miR-679-5p | 0 | 0 | 0 | 0 | 0 | 4 | 0 | 0 | 0 | 0 | 0 | 0.443998 |
| mmu-miR-6896-5p | 0 | 0 | 0 | 0 | 0 | 1 | 0 | 0 | 0 | 0 | 0 | 0.111 |
| mmu-miR-6898-5p | 1 | 0 | 0 | 0 | 0 | 0 | 0.113812 | 0 | 0 | 0 | 0 | 0 |
| mmu-miR-6899-3p | 0 | 2 | 0 | 0 | 0 | 4 | 0 | 0.250451 | 0 | 0 | 0 | 0.443998 |
| mmu-miR-690 | 16 | 24 | 26 | 23 | 12 | 36 | 1.820987 | 3.005417 | 3.020367 | 2.597943 | 1.33733 | 3.995986 |
| mmu-miR-6900-3p | 1 | 0 | 0 | 2 | 0 | 1 | 0.113812 | 0 | 0 | 0.225908 | 0 | 0.111 |
| mmu-miR-6900-5p | 0 | 0 | 2 | 0 | 0 | 0 | 0 | 0 | 0.232336 | 0 | 0 | 0 |
| mmu-miR-6902-5p | 0 | 1 | 0 | 0 | 0 | 1 | 0 | 0.125226 | 0 | 0 | 0 | 0.111 |
| mmu-miR-6909-3p | 0 | 2 | 1 | 0 | 0 | 0 | 0 | 0.250451 | 0.116168 | 0 | 0 | 0 |
| mmu-miR-6909-5p | 1 | 2 | 0 | 0 | 2 | 1 | 0.113812 | 0.250451 | 0 | 0 | 0.222888 | 0.111 |
| mmu-miR-6910-3p | 0 | 1 | 2 | 0 | 0 | 0 | 0 | 0.125226 | 0.232336 | 0 | 0 | 0 |
| mmu-miR-6911-3p | 1 | 6 | 0 | 0 | 0 | 3 | 0.113812 | 0.751354 | 0 | 0 | 0 | 0.332999 |
| mmu-miR-6912-3p | 0 | 1 | 0 | 0 | 0 | 0 | 0 | 0.125226 | 0 | 0 | 0 | 0 |
| mmu-miR-6912-5p | 0 | 0 | 1 | 0 | 0 | 0 | 0 | 0 | 0.116168 | 0 | 0 | 0 |
| mmu-miR-6913-3p | 0 | 0 | 0 | 0 | 3 | 0 | 0 | 0 | 0 | 0 | 0.334332 | 0 |
| mmu-miR-6916-5p | 0 | 0 | 0 | 0 | 0 | 1 | 0 | 0 | 0 | 0 | 0 | 0.111 |
| mmu-miR-6917-3p | 0 | 0 | 0 | 0 | 2 | 0 | 0 | 0 | 0 | 0 | 0.222888 | 0 |
| mmu-miR-6918-5p | 0 | 0 | 0 | 3 | 0 | 1 | 0 | 0 | 0 | 0.338862 | 0 | 0.111 |
| mmu-miR-6919-5p | 2 | 1 | 0 | 0 | 1 | 1 | 0.227623 | 0.125226 | 0 | 0 | 0.111444 | 0.111 |
| mmu-miR-6922-5p | 0 | 0 | 1 | 0 | 0 | 0 | 0 | 0 | 0.116168 | 0 | 0 | 0 |
| mmu-miR-6929-3p | 0 | 0 | 0 | 0 | 0 | 2 | 0 | 0 | 0 | 0 | 0 | 0.221999 |
| mmu-miR-6932-5p | 3 | 0 | 1 | 1 | 0 | 1 | 0.341435 | 0 | 0.116168 | 0.112954 | 0 | 0.111 |
| mmu-miR-6933-5p | 0 | 2 | 3 | 0 | 1 | 6 | 0 | 0.250451 | 0.348504 | 0 | 0.111444 | 0.665998 |
| mmu-miR-693-3p | 3 | 1 | 0 | 0 | 0 | 1 | 0.341435 | 0.125226 | 0 | 0 | 0 | 0.111 |
| mmu-miR-6936-3p | 0 | 1 | 0 | 0 | 0 | 0 | 0 | 0.125226 | 0 | 0 | 0 | 0 |
| mmu-miR-6937-3p | 0 | 0 | 0 | 2 | 0 | 0 | 0 | 0 | 0 | 0.225908 | 0 | 0 |
| mmu-miR-6938-3p | 2 | 0 | 0 | 0 | 0 | 0 | 0.227623 | 0 | 0 | 0 | 0 | 0 |
| mmu-miR-6940-3p | 2 | 0 | 0 | 0 | 1 | 0 | 0.227623 | 0 | 0 | 0 | 0.111444 | 0 |
| mmu-miR-6944-3p | 0 | 0 | 0 | 0 | 0 | 1 | 0 | 0 | 0 | 0 | 0 | 0.111 |
| mmu-miR-6946-5p | 1 | 0 | 0 | 0 | 0 | 0 | 0.113812 | 0 | 0 | 0 | 0 | 0 |
| mmu-miR-6948-3p | 0 | 1 | 1 | 0 | 1 | 4 | 0 | 0.125226 | 0.116168 | 0 | 0.111444 | 0.443998 |
| mmu-miR-6948-5p | 0 | 1 | 2 | 1 | 0 | 2 | 0 | 0.125226 | 0.232336 | 0.112954 | 0 | 0.221999 |
| mmu-miR-6951-5p | 0 | 0 | 0 | 1 | 0 | 0 | 0 | 0 | 0 | 0.112954 | 0 | 0 |
| mmu-miR-6952-3p | 0 | 0 | 2 | 3 | 0 | 2 | 0 | 0 | 0.232336 | 0.338862 | 0 | 0.221999 |
| mmu-miR-6955-5p | 4 | 1 | 2 | 0 | 2 | 1 | 0.455247 | 0.125226 | 0.232336 | 0 | 0.222888 | 0.111 |
| mmu-miR-6956-5p | 0 | 0 | 0 | 0 | 3 | 0 | 0 | 0 | 0 | 0 | 0.334332 | 0 |
| mmu-miR-6957-3p | 0 | 0 | 2 | 0 | 0 | 0 | 0 | 0 | 0.232336 | 0 | 0 | 0 |
| mmu-miR-6958-3p | 1 | 0 | 0 | 0 | 0 | 0 | 0.113812 | 0 | 0 | 0 | 0 | 0 |
| mmu-miR-6960-5p | 2 | 3 | 2 | 2 | 1 | 0 | 0.227623 | 0.375677 | 0.232336 | 0.225908 | 0.111444 | 0 |
| mmu-miR-6962-3p | 0 | 0 | 0 | 0 | 0 | 1 | 0 | 0 | 0 | 0 | 0 | 0.111 |
| mmu-miR-6962-5p | 2 | 0 | 0 | 0 | 0 | 0 | 0.227623 | 0 | 0 | 0 | 0 | 0 |
| mmu-miR-6965-3p | 0 | 0 | 1 | 0 | 0 | 2 | 0 | 0 | 0.116168 | 0 | 0 | 0.221999 |
| mmu-miR-6966-3p | 2 | 2 | 0 | 1 | 0 | 0 | 0.227623 | 0.250451 | 0 | 0.112954 | 0 | 0 |
| mmu-miR-6967-3p | 1 | 0 | 0 | 0 | 0 | 0 | 0.113812 | 0 | 0 | 0 | 0 | 0 |
| mmu-miR-6967-5p | 0 | 1 | 0 | 1 | 0 | 0 | 0 | 0.125226 | 0 | 0.112954 | 0 | 0 |
| mmu-miR-6969-3p | 0 | 0 | 0 | 2 | 3 | 0 | 0 | 0 | 0 | 0.225908 | 0.334332 | 0 |
| mmu-miR-6970-3p | 0 | 0 | 1 | 0 | 0 | 1 | 0 | 0 | 0.116168 | 0 | 0 | 0.111 |
| mmu-miR-6970-5p | 2 | 1 | 0 | 0 | 0 | 0 | 0.227623 | 0.125226 | 0 | 0 | 0 | 0 |
| mmu-miR-6972-3p | 0 | 0 | 0 | 1 | 1 | 0 | 0 | 0 | 0 | 0.112954 | 0.111444 | 0 |
| mmu-miR-6975-3p | 0 | 0 | 0 | 0 | 0 | 1 | 0 | 0 | 0 | 0 | 0 | 0.111 |
| mmu-miR-6975-5p | 1 | 0 | 0 | 0 | 0 | 0 | 0.113812 | 0 | 0 | 0 | 0 | 0 |
| mmu-miR-6978-3p | 0 | 0 | 0 | 0 | 0 | 1 | 0 | 0 | 0 | 0 | 0 | 0.111 |
| mmu-miR-6984-3p | 3 | 0 | 0 | 0 | 0 | 1 | 0.341435 | 0 | 0 | 0 | 0 | 0.111 |
| mmu-miR-6984-5p | 2 | 2 | 0 | 0 | 0 | 0 | 0.227623 | 0.250451 | 0 | 0 | 0 | 0 |
| mmu-miR-6986-5p | 0 | 0 | 0 | 0 | 0 | 1 | 0 | 0 | 0 | 0 | 0 | 0.111 |
| mmu-miR-6988-3p | 0 | 0 | 2 | 0 | 0 | 2 | 0 | 0 | 0.232336 | 0 | 0 | 0.221999 |
| mmu-miR-6989-3p | 0 | 0 | 0 | 1 | 0 | 0 | 0 | 0 | 0 | 0.112954 | 0 | 0 |
| mmu-miR-6990-5p | 4 | 2 | 3 | 4 | 1 | 0 | 0.455247 | 0.250451 | 0.348504 | 0.451816 | 0.111444 | 0 |
| mmu-miR-6992-3p | 0 | 1 | 0 | 0 | 0 | 0 | 0 | 0.125226 | 0 | 0 | 0 | 0 |
| mmu-miR-6992-5p | 3 | 3 | 1 | 0 | 1 | 0 | 0.341435 | 0.375677 | 0.116168 | 0 | 0.111444 | 0 |
| mmu-miR-6994-3p | 4 | 3 | 0 | 0 | 1 | 0 | 0.455247 | 0.375677 | 0 | 0 | 0.111444 | 0 |
| mmu-miR-6994-5p | 0 | 0 | 1 | 0 | 1 | 0 | 0 | 0 | 0.116168 | 0 | 0.111444 | 0 |
| mmu-miR-6997-5p | 2 | 0 | 0 | 0 | 0 | 0 | 0.227623 | 0 | 0 | 0 | 0 | 0 |
| mmu-miR-6998-3p | 2 | 0 | 2 | 0 | 0 | 0 | 0.227623 | 0 | 0.232336 | 0 | 0 | 0 |
| mmu-miR-7001-3p | 0 | 0 | 0 | 1 | 0 | 0 | 0 | 0 | 0 | 0.112954 | 0 | 0 |
| mmu-miR-7002-5p | 1 | 2 | 0 | 0 | 0 | 0 | 0.113812 | 0.250451 | 0 | 0 | 0 | 0 |
| mmu-miR-700-3p | 7 | 16 | 16 | 15 | 19 | 31 | 0.796682 | 2.003612 | 1.858687 | 1.694311 | 2.117438 | 3.440988 |
| mmu-miR-7004-5p | 1 | 1 | 1 | 0 | 0 | 0 | 0.113812 | 0.125226 | 0.116168 | 0 | 0 | 0 |
| mmu-miR-700-5p | 21 | 11 | 14 | 13 | 33 | 46 | 2.390046 | 1.377483 | 1.626352 | 1.468403 | 3.677656 | 5.105982 |
| mmu-miR-7006-3p | 0 | 1 | 0 | 1 | 0 | 0 | 0 | 0.125226 | 0 | 0.112954 | 0 | 0 |
| mmu-miR-7008-3p | 0 | 0 | 1 | 0 | 0 | 0 | 0 | 0 | 0.116168 | 0 | 0 | 0 |
| mmu-miR-7010-5p | 0 | 0 | 0 | 0 | 0 | 1 | 0 | 0 | 0 | 0 | 0 | 0.111 |
| mmu-miR-7013-3p | 1 | 0 | 0 | 0 | 0 | 0 | 0.113812 | 0 | 0 | 0 | 0 | 0 |
| mmu-miR-7015-3p | 1 | 0 | 1 | 4 | 0 | 1 | 0.113812 | 0 | 0.116168 | 0.451816 | 0 | 0.111 |
| mmu-miR-701-5p | 0 | 0 | 0 | 0 | 0 | 1 | 0 | 0 | 0 | 0 | 0 | 0.111 |
| mmu-miR-7017-5p | 4 | 4 | 0 | 0 | 1 | 1 | 0.455247 | 0.500903 | 0 | 0 | 0.111444 | 0.111 |
| mmu-miR-7018-3p | 0 | 0 | 0 | 0 | 1 | 0 | 0 | 0 | 0 | 0 | 0.111444 | 0 |
| mmu-miR-7019-3p | 1 | 0 | 0 | 1 | 0 | 0 | 0.113812 | 0 | 0 | 0.112954 | 0 | 0 |
| mmu-miR-7019-5p | 0 | 1 | 0 | 0 | 0 | 0 | 0 | 0.125226 | 0 | 0 | 0 | 0 |
| mmu-miR-7020-5p | 1 | 0 | 0 | 0 | 0 | 0 | 0.113812 | 0 | 0 | 0 | 0 | 0 |
| mmu-miR-7021-5p | 4 | 1 | 1 | 0 | 2 | 0 | 0.455247 | 0.125226 | 0.116168 | 0 | 0.222888 | 0 |
| mmu-miR-7022-5p | 0 | 0 | 0 | 0 | 1 | 0 | 0 | 0 | 0 | 0 | 0.111444 | 0 |
| mmu-miR-702-3p | 0 | 0 | 0 | 2 | 0 | 0 | 0 | 0 | 0 | 0.225908 | 0 | 0 |
| mmu-miR-7025-3p | 0 | 2 | 0 | 0 | 0 | 0 | 0 | 0.250451 | 0 | 0 | 0 | 0 |
| mmu-miR-702-5p | 1 | 2 | 0 | 0 | 0 | 0 | 0.113812 | 0.250451 | 0 | 0 | 0 | 0 |
| mmu-miR-7026-5p | 2 | 1 | 1 | 0 | 1 | 1 | 0.227623 | 0.125226 | 0.116168 | 0 | 0.111444 | 0.111 |
| mmu-miR-7027-3p | 0 | 0 | 0 | 0 | 1 | 0 | 0 | 0 | 0 | 0 | 0.111444 | 0 |
| mmu-miR-7027-5p | 0 | 0 | 0 | 0 | 0 | 1 | 0 | 0 | 0 | 0 | 0 | 0.111 |
| mmu-miR-7029-3p | 0 | 0 | 0 | 0 | 1 | 1 | 0 | 0 | 0 | 0 | 0.111444 | 0.111 |
| mmu-miR-7030-3p | 0 | 1 | 0 | 0 | 0 | 4 | 0 | 0.125226 | 0 | 0 | 0 | 0.443998 |
| mmu-miR-7031-5p | 0 | 0 | 0 | 0 | 0 | 1 | 0 | 0 | 0 | 0 | 0 | 0.111 |
| mmu-miR-7032-3p | 1 | 2 | 1 | 0 | 0 | 0 | 0.113812 | 0.250451 | 0.116168 | 0 | 0 | 0 |
| mmu-miR-7033-5p | 0 | 1 | 0 | 0 | 0 | 0 | 0 | 0.125226 | 0 | 0 | 0 | 0 |
| mmu-miR-7036b-5p | 4 | 4 | 0 | 1 | 2 | 2 | 0.455247 | 0.500903 | 0 | 0.112954 | 0.222888 | 0.221999 |
| mmu-miR-7039-5p | 1 | 0 | 0 | 0 | 0 | 0 | 0.113812 | 0 | 0 | 0 | 0 | 0 |
| mmu-miR-704 | 1 | 3 | 0 | 3 | 1 | 3 | 0.113812 | 0.375677 | 0 | 0.338862 | 0.111444 | 0.332999 |
| mmu-miR-7040-5p | 0 | 0 | 0 | 0 | 0 | 1 | 0 | 0 | 0 | 0 | 0 | 0.111 |
| mmu-miR-7041-5p | 0 | 0 | 0 | 0 | 0 | 1 | 0 | 0 | 0 | 0 | 0 | 0.111 |
| mmu-miR-7043-3p | 12 | 7 | 10 | 23 | 29 | 12 | 1.365741 | 0.87658 | 1.16168 | 2.597943 | 3.23188 | 1.331995 |
| mmu-miR-7043-5p | 0 | 0 | 0 | 0 | 1 | 0 | 0 | 0 | 0 | 0 | 0.111444 | 0 |
| mmu-miR-7046-5p | 1 | 0 | 0 | 0 | 0 | 0 | 0.113812 | 0 | 0 | 0 | 0 | 0 |
| mmu-miR-7049-3p | 0 | 0 | 0 | 0 | 0 | 2 | 0 | 0 | 0 | 0 | 0 | 0.221999 |
| mmu-miR-7049-5p | 0 | 0 | 2 | 0 | 0 | 0 | 0 | 0 | 0.232336 | 0 | 0 | 0 |
| mmu-miR-7054-5p | 0 | 0 | 0 | 1 | 0 | 0 | 0 | 0 | 0 | 0.112954 | 0 | 0 |
| mmu-miR-7057-3p | 0 | 0 | 0 | 0 | 0 | 2 | 0 | 0 | 0 | 0 | 0 | 0.221999 |
| mmu-miR-7059-5p | 0 | 0 | 0 | 0 | 0 | 2 | 0 | 0 | 0 | 0 | 0 | 0.221999 |
| mmu-miR-7064-5p | 1 | 0 | 1 | 0 | 0 | 0 | 0.113812 | 0 | 0.116168 | 0 | 0 | 0 |
| mmu-miR-7073-5p | 6 | 2 | 0 | 1 | 0 | 4 | 0.68287 | 0.250451 | 0 | 0.112954 | 0 | 0.443998 |
| mmu-miR-7075-3p | 0 | 0 | 0 | 0 | 0 | 1 | 0 | 0 | 0 | 0 | 0 | 0.111 |
| mmu-miR-7079-3p | 1 | 0 | 0 | 0 | 0 | 0 | 0.113812 | 0 | 0 | 0 | 0 | 0 |
| mmu-miR-7081-3p | 0 | 0 | 0 | 1 | 0 | 0 | 0 | 0 | 0 | 0.112954 | 0 | 0 |
| mmu-miR-7083-5p | 10 | 5 | 2 | 3 | 5 | 0 | 1.138117 | 0.626129 | 0.232336 | 0.338862 | 0.557221 | 0 |
| mmu-miR-708-3p | 13 | 11 | 14 | 65 | 83 | 271 | 1.479552 | 1.377483 | 1.626352 | 7.342013 | 9.249863 | 30.08089 |
| mmu-miR-7084-5p | 2 | 0 | 0 | 0 | 1 | 0 | 0.227623 | 0 | 0 | 0 | 0.111444 | 0 |
| mmu-miR-7085-3p | 0 | 0 | 1 | 0 | 0 | 0 | 0 | 0 | 0.116168 | 0 | 0 | 0 |
| mmu-miR-7085-5p | 0 | 0 | 1 | 0 | 0 | 0 | 0 | 0 | 0.116168 | 0 | 0 | 0 |
| mmu-miR-708-5p | 6 | 11 | 2 | 29 | 18 | 125 | 0.68287 | 1.377483 | 0.232336 | 3.275667 | 2.005994 | 13.87495 |
| mmu-miR-7087-5p | 1 | 1 | 1 | 0 | 0 | 0 | 0.113812 | 0.125226 | 0.116168 | 0 | 0 | 0 |
| mmu-miR-7089-3p | 0 | 0 | 0 | 0 | 2 | 0 | 0 | 0 | 0 | 0 | 0.222888 | 0 |
| mmu-miR-7089-5p | 0 | 1 | 0 | 0 | 0 | 0 | 0 | 0.125226 | 0 | 0 | 0 | 0 |
| mmu-miR-709 | 0 | 0 | 0 | 0 | 0 | 2 | 0 | 0 | 0 | 0 | 0 | 0.221999 |
| mmu-miR-7090-3p | 0 | 0 | 0 | 3 | 0 | 0 | 0 | 0 | 0 | 0.338862 | 0 | 0 |
| mmu-miR-7091-3p | 1 | 0 | 0 | 0 | 0 | 0 | 0.113812 | 0 | 0 | 0 | 0 | 0 |
| mmu-miR-7091-5p | 0 | 0 | 0 | 0 | 0 | 2 | 0 | 0 | 0 | 0 | 0 | 0.221999 |
| mmu-miR-7092-3p | 0 | 0 | 0 | 0 | 1 | 0 | 0 | 0 | 0 | 0 | 0.111444 | 0 |
| mmu-miR-7092-5p | 1 | 0 | 0 | 0 | 1 | 0 | 0.113812 | 0 | 0 | 0 | 0.111444 | 0 |
| mmu-miR-7093-3p | 1 | 0 | 0 | 0 | 0 | 0 | 0.113812 | 0 | 0 | 0 | 0 | 0 |
| mmu-miR-7094-3p | 0 | 0 | 0 | 2 | 0 | 1 | 0 | 0 | 0 | 0.225908 | 0 | 0.111 |
| mmu-miR-7094b-2-5p | 0 | 0 | 0 | 0 | 2 | 0 | 0 | 0 | 0 | 0 | 0.222888 | 0 |
| mmu-miR-7115-5p | 0 | 0 | 0 | 0 | 0 | 1 | 0 | 0 | 0 | 0 | 0 | 0.111 |
| mmu-miR-7116-5p | 1 | 0 | 0 | 0 | 0 | 0 | 0.113812 | 0 | 0 | 0 | 0 | 0 |
| mmu-miR-7118-3p | 1 | 1 | 2 | 0 | 0 | 3 | 0.113812 | 0.125226 | 0.232336 | 0 | 0 | 0.332999 |
| mmu-miR-7118-5p | 0 | 0 | 0 | 0 | 0 | 1 | 0 | 0 | 0 | 0 | 0 | 0.111 |
| mmu-miR-7219-3p | 11 | 8 | 5 | 13 | 12 | 14 | 1.251929 | 1.001806 | 0.58084 | 1.468403 | 1.33733 | 1.553994 |
| mmu-miR-7219-5p | 1 | 0 | 0 | 0 | 0 | 0 | 0.113812 | 0 | 0 | 0 | 0 | 0 |
| mmu-miR-7226-3p | 1 | 3 | 2 | 4 | 2 | 6 | 0.113812 | 0.375677 | 0.232336 | 0.451816 | 0.222888 | 0.665998 |
| mmu-miR-7226-5p | 0 | 0 | 0 | 0 | 1 | 0 | 0 | 0 | 0 | 0 | 0.111444 | 0 |
| mmu-miR-7228-5p | 0 | 1 | 0 | 0 | 0 | 0 | 0 | 0.125226 | 0 | 0 | 0 | 0 |
| mmu-miR-7236-3p | 0 | 0 | 1 | 0 | 1 | 0 | 0 | 0 | 0.116168 | 0 | 0.111444 | 0 |
| mmu-miR-7236-5p | 0 | 0 | 1 | 0 | 1 | 0 | 0 | 0 | 0.116168 | 0 | 0.111444 | 0 |
| mmu-miR-7237-5p | 0 | 1 | 0 | 0 | 0 | 0 | 0 | 0.125226 | 0 | 0 | 0 | 0 |
| mmu-miR-7240-5p | 1 | 0 | 0 | 0 | 0 | 0 | 0.113812 | 0 | 0 | 0 | 0 | 0 |
| mmu-miR-741-3p | 0 | 1 | 1 | 0 | 0 | 0 | 0 | 0.125226 | 0.116168 | 0 | 0 | 0 |
| mmu-miR-744-3p | 12 | 21 | 26 | 10 | 22 | 22 | 1.365741 | 2.62974 | 3.020367 | 1.12954 | 2.451771 | 2.441991 |
| mmu-miR-744-5p | 2171 | 1818 | 2059 | 957 | 1199 | 1112 | 247.0852 | 227.6604 | 239.1898 | 108.097 | 133.6215 | 123.4316 |
| mmu-miR-758-3p | 0 | 0 | 0 | 0 | 2 | 0 | 0 | 0 | 0 | 0 | 0.222888 | 0 |
| mmu-miR-7646-5p | 2 | 0 | 2 | 0 | 0 | 0 | 0.227623 | 0 | 0.232336 | 0 | 0 | 0 |
| mmu-miR-7647-5p | 1 | 0 | 0 | 0 | 0 | 0 | 0.113812 | 0 | 0 | 0 | 0 | 0 |
| mmu-miR-7649-3p | 0 | 1 | 0 | 1 | 0 | 1 | 0 | 0.125226 | 0 | 0.112954 | 0 | 0.111 |
| mmu-miR-7649-5p | 0 | 0 | 2 | 2 | 0 | 7 | 0 | 0 | 0.232336 | 0.225908 | 0 | 0.776997 |
| mmu-miR-7651-5p | 1 | 1 | 0 | 0 | 0 | 1 | 0.113812 | 0.125226 | 0 | 0 | 0 | 0.111 |
| mmu-miR-7652-3p | 0 | 0 | 2 | 0 | 1 | 0 | 0 | 0 | 0.232336 | 0 | 0.111444 | 0 |
| mmu-miR-7655-3p | 0 | 0 | 0 | 0 | 1 | 0 | 0 | 0 | 0 | 0 | 0.111444 | 0 |
| mmu-miR-7656-5p | 0 | 0 | 0 | 0 | 1 | 3 | 0 | 0 | 0 | 0 | 0.111444 | 0.332999 |
| mmu-miR-7657-3p | 2 | 0 | 1 | 1 | 0 | 0 | 0.227623 | 0 | 0.116168 | 0.112954 | 0 | 0 |
| mmu-miR-7657-5p | 2 | 1 | 2 | 0 | 0 | 0 | 0.227623 | 0.125226 | 0.232336 | 0 | 0 | 0 |
| mmu-miR-7658-5p | 2 | 0 | 0 | 0 | 0 | 0 | 0.227623 | 0 | 0 | 0 | 0 | 0 |
| mmu-miR-7662-3p | 0 | 1 | 0 | 0 | 0 | 0 | 0 | 0.125226 | 0 | 0 | 0 | 0 |
| mmu-miR-7667-3p | 1 | 2 | 0 | 0 | 0 | 4 | 0.113812 | 0.250451 | 0 | 0 | 0 | 0.443998 |
| mmu-miR-7667-5p | 1 | 0 | 1 | 0 | 0 | 0 | 0.113812 | 0 | 0.116168 | 0 | 0 | 0 |
| mmu-miR-7669-3p | 0 | 0 | 0 | 1 | 0 | 0 | 0 | 0 | 0 | 0.112954 | 0 | 0 |
| mmu-miR-7669-5p | 1 | 1 | 0 | 0 | 1 | 0 | 0.113812 | 0.125226 | 0 | 0 | 0.111444 | 0 |
| mmu-miR-7670-3p | 2 | 3 | 3 | 3 | 3 | 0 | 0.227623 | 0.375677 | 0.348504 | 0.338862 | 0.334332 | 0 |
| mmu-miR-7670-5p | 1 | 0 | 0 | 0 | 0 | 1 | 0.113812 | 0 | 0 | 0 | 0 | 0.111 |
| mmu-miR-7671-3p | 0 | 0 | 1 | 0 | 0 | 0 | 0 | 0 | 0.116168 | 0 | 0 | 0 |
| mmu-miR-7672-3p | 0 | 0 | 1 | 0 | 0 | 0 | 0 | 0 | 0.116168 | 0 | 0 | 0 |
| mmu-miR-7673-5p | 1 | 1 | 2 | 1 | 0 | 1 | 0.113812 | 0.125226 | 0.232336 | 0.112954 | 0 | 0.111 |
| mmu-miR-7675-3p | 0 | 0 | 1 | 0 | 0 | 0 | 0 | 0 | 0.116168 | 0 | 0 | 0 |
| mmu-miR-7675-5p | 0 | 0 | 1 | 0 | 0 | 0 | 0 | 0 | 0.116168 | 0 | 0 | 0 |
| mmu-miR-7676-3p | 0 | 1 | 0 | 0 | 0 | 8 | 0 | 0.125226 | 0 | 0 | 0 | 0.887997 |
| mmu-miR-7677-3p | 1 | 0 | 0 | 0 | 0 | 1 | 0.113812 | 0 | 0 | 0 | 0 | 0.111 |
| mmu-miR-7679-3p | 0 | 0 | 0 | 0 | 0 | 1 | 0 | 0 | 0 | 0 | 0 | 0.111 |
| mmu-miR-7679-5p | 0 | 2 | 1 | 0 | 0 | 0 | 0 | 0.250451 | 0.116168 | 0 | 0 | 0 |
| mmu-miR-7683-5p | 0 | 1 | 0 | 0 | 0 | 1 | 0 | 0.125226 | 0 | 0 | 0 | 0.111 |
| mmu-miR-7685-5p | 0 | 0 | 0 | 2 | 0 | 0 | 0 | 0 | 0 | 0.225908 | 0 | 0 |
| mmu-miR-7688-5p | 16 | 10 | 9 | 2 | 2 | 2 | 1.820987 | 1.252257 | 1.045512 | 0.225908 | 0.222888 | 0.221999 |
| mmu-miR-770-3p | 1 | 0 | 0 | 6 | 3 | 2 | 0.113812 | 0 | 0 | 0.677724 | 0.334332 | 0.221999 |
| mmu-miR-7a-1-3p | 79 | 75 | 52 | 96 | 105 | 123 | 8.991125 | 9.391929 | 6.040734 | 10.84359 | 11.70163 | 13.65295 |
| mmu-miR-7a-2-3p | 0 | 2 | 4 | 2 | 5 | 7 | 0 | 0.250451 | 0.464672 | 0.225908 | 0.557221 | 0.776997 |
| mmu-miR-7a-5p | 89970 | 84966 | 80784 | 51038 | 54655 | 54684 | 10239.64 | 10639.93 | 9384.513 | 5764.949 | 6090.979 | 6069.902 |
| mmu-miR-7b-5p | 484 | 531 | 475 | 965 | 1069 | 1391 | 55.08487 | 66.49486 | 55.17978 | 109.0007 | 119.1338 | 154.4005 |
| mmu-miR-802-3p | 96 | 90 | 96 | 59 | 62 | 63 | 10.92592 | 11.27031 | 11.15212 | 6.664289 | 6.909536 | 6.992975 |
| mmu-miR-802-5p | 407 | 344 | 400 | 585 | 517 | 517 | 46.32137 | 43.07765 | 46.46719 | 66.07812 | 57.61662 | 57.3868 |
| mmu-miR-8091 | 2 | 0 | 0 | 0 | 0 | 0 | 0.227623 | 0 | 0 | 0 | 0 | 0 |
| mmu-miR-8094 | 2 | 0 | 0 | 2 | 0 | 1 | 0.227623 | 0 | 0 | 0.225908 | 0 | 0.111 |
| mmu-miR-8097 | 4 | 5 | 7 | 5 | 4 | 1 | 0.455247 | 0.626129 | 0.813176 | 0.56477 | 0.445777 | 0.111 |
| mmu-miR-8103 | 1 | 4 | 10 | 2 | 3 | 17 | 0.113812 | 0.500903 | 1.16168 | 0.225908 | 0.334332 | 1.886993 |
| mmu-miR-8105 | 2 | 0 | 0 | 0 | 0 | 0 | 0.227623 | 0 | 0 | 0 | 0 | 0 |
| mmu-miR-8106 | 0 | 1 | 0 | 0 | 0 | 0 | 0 | 0.125226 | 0 | 0 | 0 | 0 |
| mmu-miR-8107 | 0 | 0 | 0 | 0 | 0 | 1 | 0 | 0 | 0 | 0 | 0 | 0.111 |
| mmu-miR-8111 | 0 | 1 | 0 | 0 | 2 | 1 | 0 | 0.125226 | 0 | 0 | 0.222888 | 0.111 |
| mmu-miR-8112 | 5 | 3 | 0 | 0 | 3 | 2 | 0.569059 | 0.375677 | 0 | 0 | 0.334332 | 0.221999 |
| mmu-miR-8114 | 1 | 0 | 2 | 0 | 1 | 10 | 0.113812 | 0 | 0.232336 | 0 | 0.111444 | 1.109996 |
| mmu-miR-8116 | 0 | 1 | 0 | 0 | 0 | 0 | 0 | 0.125226 | 0 | 0 | 0 | 0 |
| mmu-miR-8118 | 4 | 0 | 2 | 0 | 0 | 1 | 0.455247 | 0 | 0.232336 | 0 | 0 | 0.111 |
| mmu-miR-8120 | 2 | 3 | 4 | 2 | 2 | 2 | 0.227623 | 0.375677 | 0.464672 | 0.225908 | 0.222888 | 0.221999 |
| mmu-miR-871-5p | 0 | 0 | 1 | 0 | 0 | 0 | 0 | 0 | 0.116168 | 0 | 0 | 0 |
| mmu-miR-872-3p | 43 | 47 | 47 | 61 | 56 | 89 | 4.893904 | 5.885609 | 5.459894 | 6.890197 | 6.240871 | 9.878965 |
| mmu-miR-872-5p | 1065 | 925 | 1089 | 1175 | 1243 | 1776 | 121.2095 | 115.8338 | 126.5069 | 132.721 | 138.5251 | 197.1353 |
| mmu-miR-873a-5p | 0 | 0 | 0 | 0 | 5 | 11 | 0 | 0 | 0 | 0 | 0.557221 | 1.220996 |
| mmu-miR-874-3p | 3 | 6 | 6 | 7 | 5 | 8 | 0.341435 | 0.751354 | 0.697008 | 0.790678 | 0.557221 | 0.887997 |
| mmu-miR-875-5p | 0 | 0 | 0 | 0 | 1 | 0 | 0 | 0 | 0 | 0 | 0.111444 | 0 |
| mmu-miR-877-3p | 10 | 7 | 6 | 3 | 4 | 7 | 1.138117 | 0.87658 | 0.697008 | 0.338862 | 0.445777 | 0.776997 |
| mmu-miR-877-5p | 24 | 22 | 14 | 4 | 16 | 12 | 2.731481 | 2.754966 | 1.626352 | 0.451816 | 1.783106 | 1.331995 |
| mmu-miR-878-3p | 0 | 1 | 0 | 0 | 0 | 0 | 0 | 0.125226 | 0 | 0 | 0 | 0 |
| mmu-miR-878-5p | 0 | 0 | 0 | 0 | 2 | 0 | 0 | 0 | 0 | 0 | 0.222888 | 0 |
| mmu-miR-879-5p | 0 | 0 | 0 | 1 | 0 | 8 | 0 | 0 | 0 | 0.112954 | 0 | 0.887997 |
| mmu-miR-881-3p | 0 | 2 | 1 | 2 | 2 | 4 | 0 | 0.250451 | 0.116168 | 0.225908 | 0.222888 | 0.443998 |
| mmu-miR-92a-1-5p | 34 | 30 | 32 | 15 | 15 | 15 | 3.869598 | 3.756772 | 3.717375 | 1.694311 | 1.671662 | 1.664994 |
| mmu-miR-92a-2-5p | 0 | 0 | 0 | 3 | 1 | 1 | 0 | 0 | 0 | 0.338862 | 0.111444 | 0.111 |
| mmu-miR-92a-3p | 5046 | 4451 | 5033 | 4177 | 4997 | 6145 | 574.2939 | 557.3797 | 584.6734 | 471.809 | 556.8863 | 682.0926 |
| mmu-miR-92b-3p | 6 | 7 | 4 | 9 | 5 | 19 | 0.68287 | 0.87658 | 0.464672 | 1.016586 | 0.557221 | 2.108993 |
| mmu-miR-92b-5p | 0 | 0 | 0 | 0 | 0 | 2 | 0 | 0 | 0 | 0 | 0 | 0.221999 |
| mmu-miR-93-3p | 10 | 18 | 13 | 18 | 22 | 24 | 1.138117 | 2.254063 | 1.510184 | 2.033173 | 2.451771 | 2.663991 |
| mmu-miR-935 | 1 | 0 | 0 | 0 | 1 | 0 | 0.113812 | 0 | 0 | 0 | 0.111444 | 0 |
| mmu-miR-93-5p | 4334 | 4016 | 4235 | 5802 | 5620 | 5691 | 493.26 | 502.9065 | 491.9713 | 655.3594 | 626.316 | 631.6988 |
| mmu-miR-9-3p | 3 | 2 | 4 | 10 | 14 | 29 | 0.341435 | 0.250451 | 0.464672 | 1.12954 | 1.560218 | 3.218989 |
| mmu-miR-9-5p | 366 | 273 | 238 | 701 | 903 | 2269 | 41.65509 | 34.18662 | 27.64798 | 79.18078 | 100.6341 | 251.8581 |
| mmu-miR-96-3p | 1 | 1 | 3 | 2 | 1 | 0 | 0.113812 | 0.125226 | 0.348504 | 0.225908 | 0.111444 | 0 |
| mmu-miR-96-5p | 1185 | 942 | 998 | 1864 | 1444 | 1171 | 134.8669 | 117.9626 | 115.9356 | 210.5463 | 160.9253 | 129.9805 |
| mmu-miR-9769-3p | 3 | 0 | 2 | 1 | 0 | 1 | 0.341435 | 0 | 0.232336 | 0.112954 | 0 | 0.111 |
| mmu-miR-98-3p | 7 | 14 | 6 | 9 | 14 | 28 | 0.796682 | 1.75316 | 0.697008 | 1.016586 | 1.560218 | 3.107989 |
| mmu-miR-98-5p | 771 | 742 | 764 | 775 | 880 | 993 | 87.74883 | 92.91748 | 88.75233 | 87.53938 | 98.07084 | 110.2226 |
| mmu-miR-99a-3p | 0 | 0 | 0 | 0 | 1 | 1 | 0 | 0 | 0 | 0 | 0.111444 | 0.111 |
| mmu-miR-99a-5p | 452 | 176 | 209 | 1326 | 1314 | 4030 | 51.44289 | 22.03973 | 24.27911 | 149.7771 | 146.4376 | 447.3284 |
| mmu-miR-99b-3p | 11 | 6 | 13 | 31 | 41 | 127 | 1.251929 | 0.751354 | 1.510184 | 3.501575 | 4.569209 | 14.09695 |
| mmu-miR-99b-5p | 539 | 370 | 443 | 1523 | 1715 | 5882 | 61.34451 | 46.33352 | 51.46241 | 172.029 | 191.1267 | 652.8997 |
| mmu-miR-9b-3p | 366 | 272 | 237 | 698 | 898 | 2266 | 41.65509 | 34.0614 | 27.53181 | 78.84192 | 100.0768 | 251.5251 |
| novel_151 | 2 | 0 | 0 | 0 | 0 | 0 | 0.227623 | 0 | 0 | 0 | 0 | 0 |
| novel_165 | 0 | 2 | 0 | 0 | 0 | 0 | 0 | 0.250451 | 0 | 0 | 0 | 0 |
| novel_169 | 4 | 3 | 3 | 1 | 2 | 6 | 0.455247 | 0.375677 | 0.348504 | 0.112954 | 0.222888 | 0.665998 |
| novel_182 | 1 | 0 | 1 | 1 | 0 | 3 | 0.113812 | 0 | 0.116168 | 0.112954 | 0 | 0.332999 |
| novel_195 | 0 | 1 | 6 | 3 | 6 | 5 | 0 | 0.125226 | 0.697008 | 0.338862 | 0.668665 | 0.554998 |
| novel_205 | 1 | 0 | 0 | 2 | 0 | 1 | 0.113812 | 0 | 0 | 0.225908 | 0 | 0.111 |
| novel_229 | 5 | 11 | 5 | 2 | 7 | 12 | 0.569059 | 1.377483 | 0.58084 | 0.225908 | 0.780109 | 1.331995 |
| novel_246 | 0 | 0 | 0 | 0 | 2 | 3 | 0 | 0 | 0 | 0 | 0.222888 | 0.332999 |
| novel_272 | 0 | 0 | 0 | 5 | 3 | 18 | 0 | 0 | 0 | 0.56477 | 0.334332 | 1.997993 |
| novel_281 | 0 | 1 | 2 | 2 | 0 | 0 | 0 | 0.125226 | 0.232336 | 0.225908 | 0 | 0 |
